# Azo group(s) in selected macrocyclic compounds

**DOI:** 10.1007/s10847-017-0779-4

**Published:** 2018-01-08

**Authors:** Ewa Wagner-Wysiecka, Natalia Łukasik, Jan F. Biernat, Elżbieta Luboch

**Affiliations:** Department of Chemistry and Technology of Functional Materials, Faculty of Chemistry, Gdańsk University of Technology, Narutowicza Street 11/12, 80-233 Gdańsk, Poland

**Keywords:** Macrocyclic compounds, Azo group, *Trans–cis* isomerization, Host–guest interactions, Molecular switches

## Abstract

Azobenzene derivatives due to their photo- and electroactive properties are an important group of compounds finding applications in diverse fields. Due to the possibility of controlling the *trans–cis* isomerization, azo-bearing structures are ideal building blocks for development of e.g. nanomaterials, smart polymers, molecular containers, photoswitches, and sensors. Important role play also macrocyclic compounds well known for their interesting binding properties. In this article selected macrocyclic compounds bearing azo group(s) are comprehensively described. Here, the relationship between compounds’ structure and their properties (as e.g. ability to guest complexation, supramolecular structure formation, switching and motion) is reviewed.

## Introduction

The year 2017 appears to be a very special for supramolecular chemistry. 50 years ago Charles Pedersen [1] published papers describing the syntheses and completely untypical and unknown until that time intriguing complexing properties of macrocyclic polyethers, i.e. crown ethers [2, 3]. The discovery turned out to be a milestone in chemistry that changed the whole chemical world, gave new fascination and opened up new perespectives for science and technology. Crown ethers are excellent example of unexpected discovery that gained worldwide fame. Since discovery of crown ethers, many their applications have been developed, for example in chromatography [4, 5], sample preparations [6], catalysis [7–9], and chemical sensing [10].

Macrocyclic compounds had entered the laboratories all over the world, in particular after the discovery of macrocycles containing oxygen and nitrogen electron donors, being the base for three dimensional cryptands, synthesized and studied by Lehn [11, 12] and spherands, obtained and investigated by Cram [13–16]. All these discoveries initiated host–guest [17, 18] and supramolecular chemistry [19–22]. For their achievements Pedersen [23], Cram [18] and Lehn [20] were honored in 1987 with a Nobel Prize. The Nobel Prize in Chemistry 2016 was awarded to: Jean-Pierre Sauvage, Sir J. Fraser Stoddart and Ben L. Feringa “for the design and synthesis of molecular machines” [24–26], which have close relationship with the above mentioned branches of chemistry.

A year after Pedersen’s publication on crown ethers and their unique metal cation binding abilities, Park and Simmons published work on macrobicyclic amines i.e. catapinands, the first anion receptors [27–29]. Since that time supramolecular chemistry of anions for many years seemed to be almost forgotten, but last two decades were a renaissance of anion recognition studies [30–33].

Within the last 50-years a lot of macrocyclic compounds of sophisticated structures have been synthesized and investigated [34]. The skeleton of the vast majority of macrocyles can be more or less easily modified by introducing functional groups, which bring about additional chemical or physical features in comparison to the parent compounds as well as to the respective supramolecular species. Functionalized supramolecular systems can be applied in many branches of science and life [35], including e.g. the development of new analytical [36–41] and therapeutic [42–44] systems, modern, intelligent (nano)materials [45–50] and molecular devices and machines [51–57].

One of the most convenient and useful functionalization of macrocyclic compounds is the introduction of azo group(s): incorported in the ring or on its periphery. Azo moiety due to its ability to alter the geometry upon photochemical or thermal *trans–cis* isomerization can be utilized as a light triggered switch in vast variety of functional materials such as for example molecular containers, polymers, supramolecular protein channels, and sensors. As upon photoisomerization process of azo bearing molecules electromagnetic radiation is converted to mechanical work, those compounds can be used in light-driven molecular machines. Here, we present an extensive review of selected azomacrocyclic compounds with the special focus on supramolecular interactions (host–guest complex formation, self-assembly) and *trans–cis* isomerization of azo group.

## Azobenzene and its derivatives

The properties and functions of the supramolecular systems can be controlled by external stimuli such as changing of pH, temperature, irradiation with the selected wavelength, action with electric or magnetic field. For specific goals, moieties sensitive to one or more of the above factors must be present or introduced to macrocycle structure upon its functionalization. The synthetic routes leading to macrocyclic compounds are often laborious, hence additional functionalization preferably needs relatively simple procedures. A nice example of relatively easy-to-implement functional unit with photo- and redox active properties is the azo group –$\bar {\text{N}}{=}\bar {\text{N}}$–, which is also pH sensitive.

**Fig. 1:**
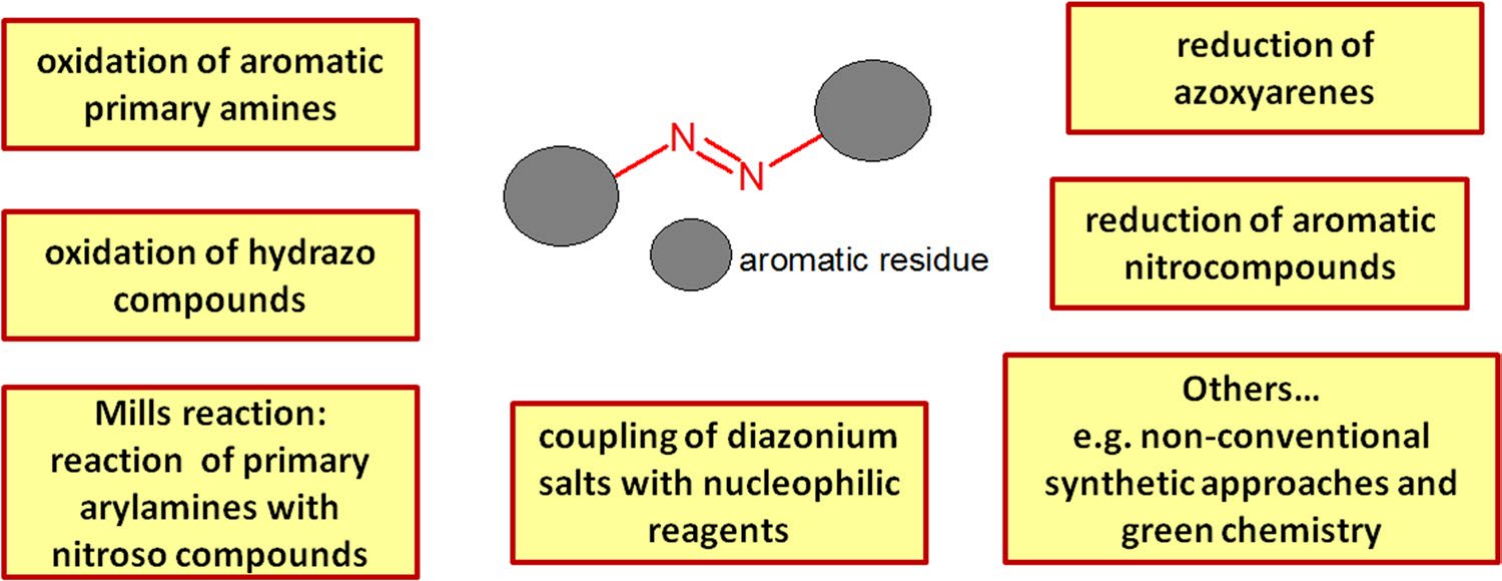
Schematically: the main methods for the synthesis of azo compounds

Azo compounds are one of the oldest synthesized organic compounds, being produced till now on a large scale in dye industry [58]. The main synthetic approach is based on diazotization reaction discovered by Peter Griess in nineteenth century. The most common methods of azo group incorporation are schematically shown in Fig. 1. Nowadays, diverse modifications of the orginal process of diazocoupling are available; also new, synthetic procedures are proposed for preparation of azo compounds for varied purposes [59–66], including methods identified as environmentally friendly [67–69].

**Fig. 2:**
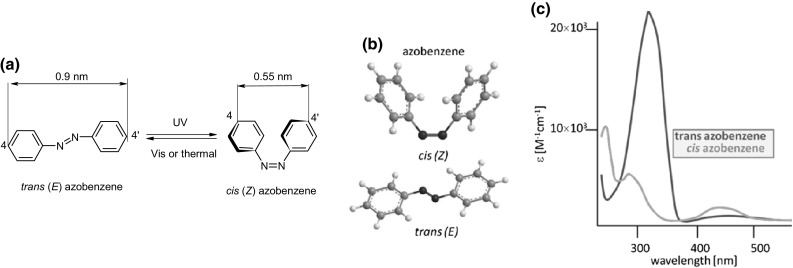
**a** *Trans* and *cis* isomers of azobenzene and reversible photoisomerization process, **b** models of *cis* and *trans* isomers of azobenzene, **c** schematic UV–Vis spectra of *trans* and *cis* isomers of azobenzene

Colored azobenzenes and their more sophisticated derivatives, among others, can undergo light-driven reversible *trans*–*cis* (*E*⇄*Z*) isomerization. The reversible *E*⇄*Z* photoisomerization of azobenzene presents well-understood process widely used for construction of light-driven functional molecules for energy storage or conversion of light energy into mechanical motion, exemplified by molecular devices and machines [70]. *Cis* isomer of azobenzene was discovered in 1937 by Hartley [71]. *Trans* (*E*) and *cis* (*Z*) azobenzene isomers are shown in Fig. 2 that also illustrates the reversible isomerization.

*Trans* isomer of azobenzene is thermodynamically more stable than the *cis* isomer. In most cases *trans*→*cis* isomerization occurs upon irradiation with UV light (Fig. 2a). However, azobenzene derivatives undergoing reversible *trans*⇄*cis* isomerization upon visible light illumination have been also reported [72–74]. Such molecular switches are more applicable and safer for biological uses where harmful ultraviolet light should be avoided.

The *cis*⇄*trans* isomerization may occur by spontaneous thermal back reaction or reverse photoisomerization cycle.

The light-driven reversible *E*⇄*Z* isomerization of azobenzene is associated with substantial changes of structure, size, geometry and physical properties. Structural changes of azobenzene moiety inbuilt into a larger or more complicated compound affect also the behavior and properties of the azo-functionalized molecular systems like it is for example in photoswitches.

Dipole moment of *trans* isomer of azobenzene is near zero. *Cis* isomer of azobenzene has dipole moment 3.1 D, what determines hydrophobic/hydrophilic character of isomers. *Trans* (*E*) azobenzene is almost planar, opposite to *cis* (*Z*) isomer. In solid state in *cis* azobenzene the parallel phenyl rings are twisted 56° out of the plane of the azo group (Fig. 2). The different geometry of *trans* and *cis* isomers of azobenzene affects their UV–Vis spectra. The spectra (Fig. 2c) of *trans* and *cis* isomers are overlapping, but differ significantly. Band at ~ 440 nm originating from n→π* transition is more distinct for *cis* isomer. Strong absorption band at ~ 320 nm for *trans* isomer can be attributed to π→π* transition. In a spectrum of *cis*-azobenzene less intensive π→π* transition bands are observed at lower wavelength. The spectral differences cause different colors of both isomers, what makes the observation of isomerization process possible also in non-instrumental manner (by naked eye). Spectral properties of azobenzene derivatives are strongly dependent on the substituents in phenyl rings.

Azobenzene can also act as an important functional unit if incorporated into electrochromic materials (ECMs), which properties can be stimulated by applied potential. Such substances are outstanding candidates for materials used for production of electronic paper [75–78] or dual-stimuli-responsive systems [79, 80].

**Scheme 1:**
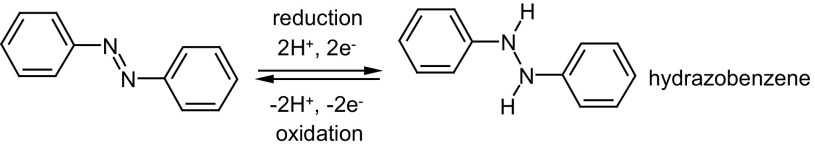
The electrochemical reduction–oxidation of azobenzene

The electrochemistry of azobenzene and its derivatives in different solvents was studied exhaustively in details for both *trans* and *cis* isomers [81–85]. It was found that the electrochemical reduction of azobenzene is strongly dependent on conditions, such as type of the solvent, pH or reagent concentrations. However, in general it can be summarized that the reduction of azobenzene occurs in a single two electrons, two protons process with a final formation of hydrazobenzene. The simplified way of the electrochemical reduction of azobenzene is shown in Scheme 1.

The properties of self-assembled monolayers of azobenzene derivatives—also macrocyclic—on different surfaces [86–92] showed, that such materials are promising candidates for molecular devices for energy storage and conversion.

## Cyclic and macrocyclic derivatives of azobenzene(s)

### Small rings

**Scheme 2:**
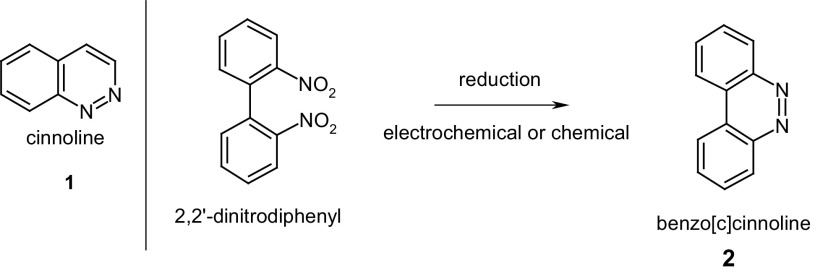
Cinnoline and reductive cyclization of 2,2′-dinitrobiphenyl as preparation method of benzo[*c*]cinnoline

Derivatives of cinnoline **1**, e.g. benzo[*c*]cinnoline **2** (Scheme 2) can be considered as structural, cyclic analogs of azobenzene. These compounds are used in manufacturing of dyes, electrochromic polymers, coloured polyamide fibers and have microbial and herbicidal activities [93, 94]. Cinnolines were also studied as potential anticancer agents [95, 96]. The reduction of 2,2′-dinitrobiphenyl to 3,4-benzocinnoline (benzo[*c*]cinnoline) **2** (Scheme 2) was first described by Wohlfart [97] and later by other groups [98–107].

The crystal structure of benzo[*c*]cinnoline complex with ytterbium Yb(BC)_3_(thf)_2_ (BC = benzo[*c*]cinnoline) was described [108]. Fe_2_(BC)(CO)_6_ complex was examined as a candidate for a new structural and functional model for [FeFe]-hydrogenases [109, 110].

Modified with benzo[*c*]cinnoline or its derivatives surfaces of e.g. glassy carbon [111, 112], gold [113] or platinum [114, 115] are often used in organic, inorganic, and biochemical catalytic transformations.

Öztürk et al. [116] reported an amperometric lactate biosensor based on a carbon paste electrode modified with benzo[c]cinnoline and multiwalled carbon nanotubes. Its characteristics showed, that it can be used for determination of lactate in human serum. Incorporation of benzo[*c*]cinnoline moieties into poly[2-methoxy-5-(2-ethylhexyloxy)-1,4-phenylenevinylene] (MEH-PPV) indicated that p-type semiconductors based on the above polymer can be transformed into n-type materials [117].

**Fig. 3:**
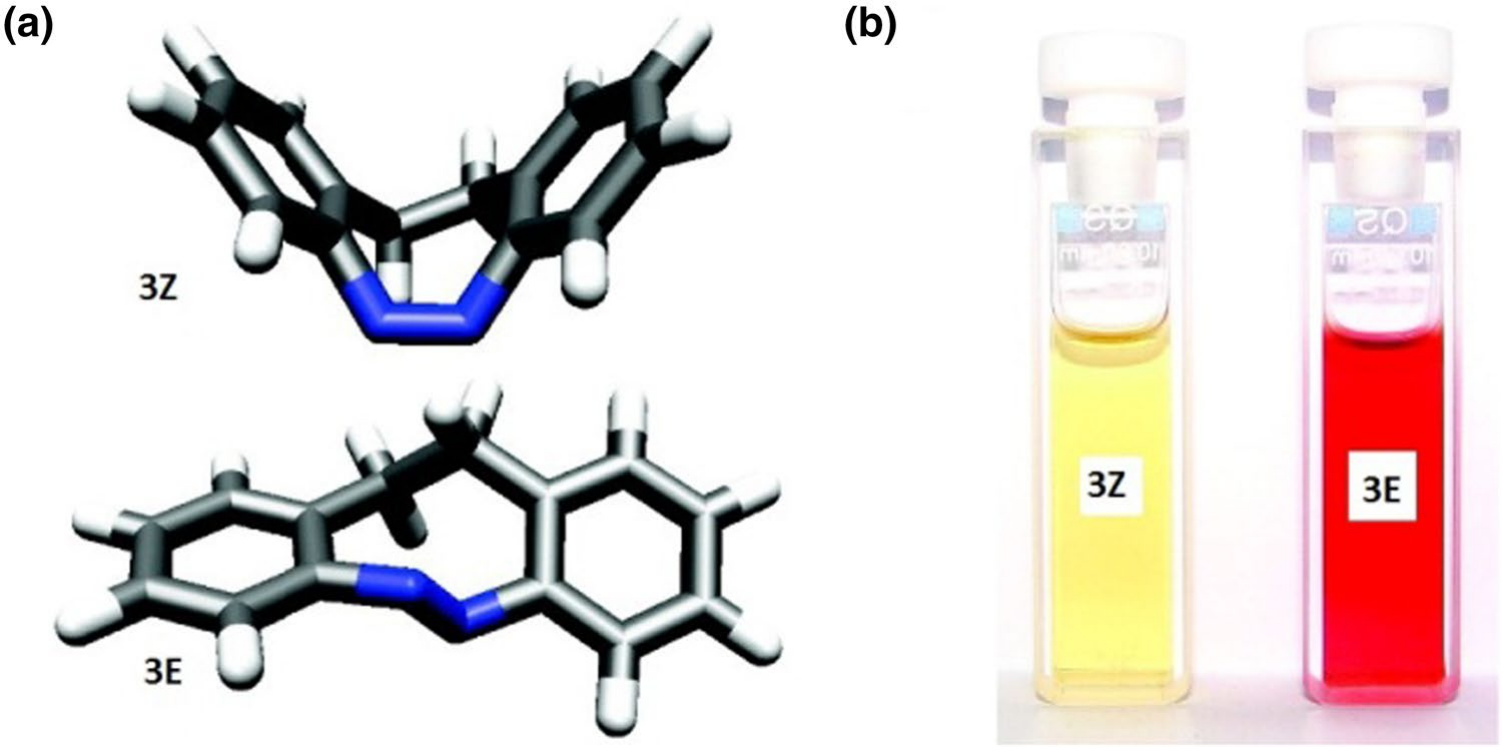
**a** 5,6-dihydrodibenzo[c,g][1,2]diazocine (**3**): equilibrium structures of **3Z** and **3E** in the electronic ground states from quantum chemical calculations at the B3LYP/def2-TZVP level of theory using the TURBOMOLE program and **b** colors of **3***Z* before irradiation and color **3***E* upon irradiation in *n*-hexane. Adapted with permission from [119]. Copyright 2009 American Chemical Society

Larger analog of cinnoline,  (5,6-dihydrodibenzo[*c*,*g*][1,2]diazocine) (Fig. 3, compound **3**) comprising azobenzene moiety joined by ethylene bridge at 2,2′-positions was identified as a molecular switch with interesting photochemical characteristics [118–120].

Interestingly, in this case *cis* isomer is thermodynamically more stable than *trans* isomer. Remarkably, both isomers of **3** have well pronounced n→π* bands in UV–Vis absorption spectra. Reversible *trans* to *cis* photoisomerization occurs with efficiency close to 100% under the illumination with visible light of 480–550 nm. The back process of rapid kinetics can be achieved at near ultraviolet at 380–400 nm.

### Cyclic oligomers of azobenzene (oligoazobenzenophanes)

**Fig. 4:**
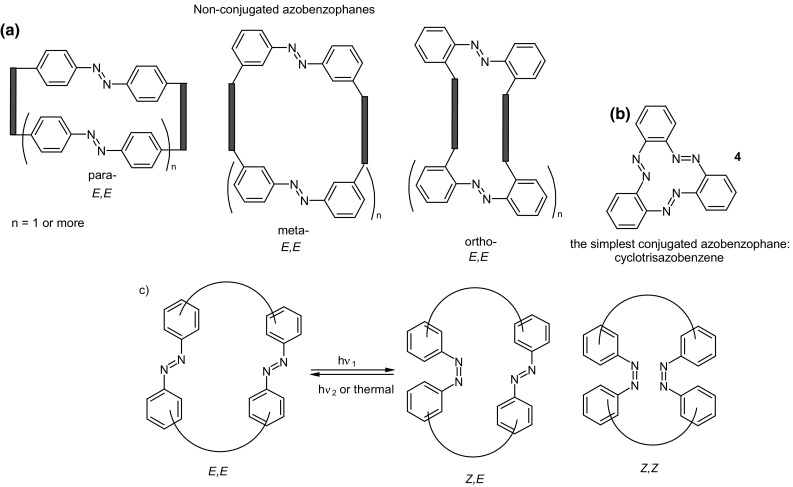
Azobenzophanes, schematic view: **a** joined in *para*-, *meta-* and *ortho*- positions (*E,E* isomers), **b** the simplest conjugated azobenzophane: cyclotrisazobenzene, **c** reversible isomerization of azobenzophanes

Oligoazobenzenophanes are compounds consisting of at least two or more azobenzene units forming macrocycles. Azobenzene moieties can be joined in *para-, meta-* or *ortho*- positions by sp^3^ hybridized spacers with or without heteroatoms forming relatively flexible, non-conjugated azobenzophanes (Fig. 4a). More rigid, conjugated azobenzophanes are obtained by joining azobenzene units without sp^3^ tether. As an example of conjugated azobenzophane the simplest cyclotrisazobenzene is shown in Fig. 4b.

Synthetic procedures leading to azobenzophanes involve also approaches typical for macrocyclization, e.g. high dilution technique or template synthesis. The formation of azo group can be the final step of ring closure or can be achieved from substrate(s) bearing functional group(s) by substitution or condensation reactions. An exhaustive review on synthetic protocols was published by Reuter and Wegner [121] that shows preparation of vast varieties of azobenzophane skeletons by cyclizations based on nucleophilic reactions, Schiff bases condensations, reductive or oxidative azocouplings, palladium catalyzed *N*-arylations, and electrophilic aromatic substitutions of diazonium salts.

The utility of azobenzophanes lies in reversible photoisomerization. Opposite to azobenzene for which only two possible states *Z* or *E* can be achieved by photoisomerization, macrocyclic azobenzophanes offer multiple molecular states, depending on the number of azo units. For example, for azobenzophane composed of two azobenzene fragments three states can be considered: *E,E, E,Z* and *Z,Z* (Fig. 4c) with the ratio of the isomers depending on e.g. the structure of macrocycle and photoisomerization conditions. The simplest conjugated azobenzophane cyclotrisazobenzene **4** (Fig. 4b) exits only in all*-E* form and has no tendency to be converted into *Z* form under illumination [121]. Unusual behavior of cyclotrisazobenzene was exhaustively investigated by Dreuw and Wachtveitl [122]. According to experimental and theoretical studies on ultrafast dynamics of this macrocycle the authors stated that the structural constrains prevent isomerization of azo units. The azo bonds respond elastically to the motion along the isomerization coordinates leading to complete and ultrafast dissipation of the UV excitation as heat. It was proposed that the molecules of this type can be used as UV absorbers e.g. in sunscreens.

**Fig. 5:**
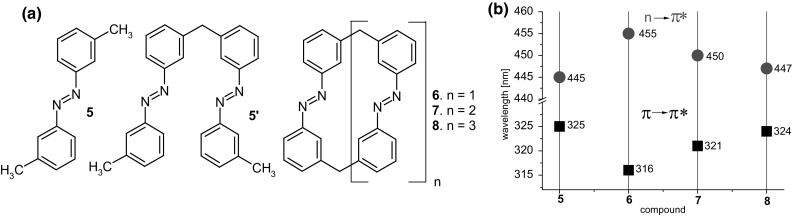
**a** Azobenzophanes **6**–**8** and acyclic compounds **5** and **5**′ obtained by Tamaoki and co-workers [123, 124], **b** the position of π→π* and n→π* absorption bands in UV–Vis spectra (benzene) of macrocyclic compounds **6**–**8** and acyclic analogs **5** [123]

Azobenzophanes of various structures are studied inter alia as metal cation complexing reagents. Tamaoki and co-workers [123] have obtained a series of azobenzophanes **6**–**8** (Fig. 5) by reductive macrocyclization of bis(3-nitrophenyl)methane under high dilution conditions. Macrocycles constructed of two, three or four azobenzene units with methylene linkers were isolated as all-*E* isomers. For comparative studies *trans-*3,3′-dimethylazobenzene **5** was prepared (Fig. 5).

The position of UV–Vis absorption maxima for compounds **6**–**8** and **5** registered in benzene are ring size dependent. The shift of the main band (π→π*) towards longer wavelength can be ordered as follows: **8** > **7** > **6** and reverse order for n→π* (cf. Fig. 5b) and can be associated with steric distortion of the azobenzene moieties. The position of the main absorption band of the largest compound **8** is comparable to a spectrum of open chain analog *trans-*3,3′-dimethylazobenzene.

**Fig. 6:**
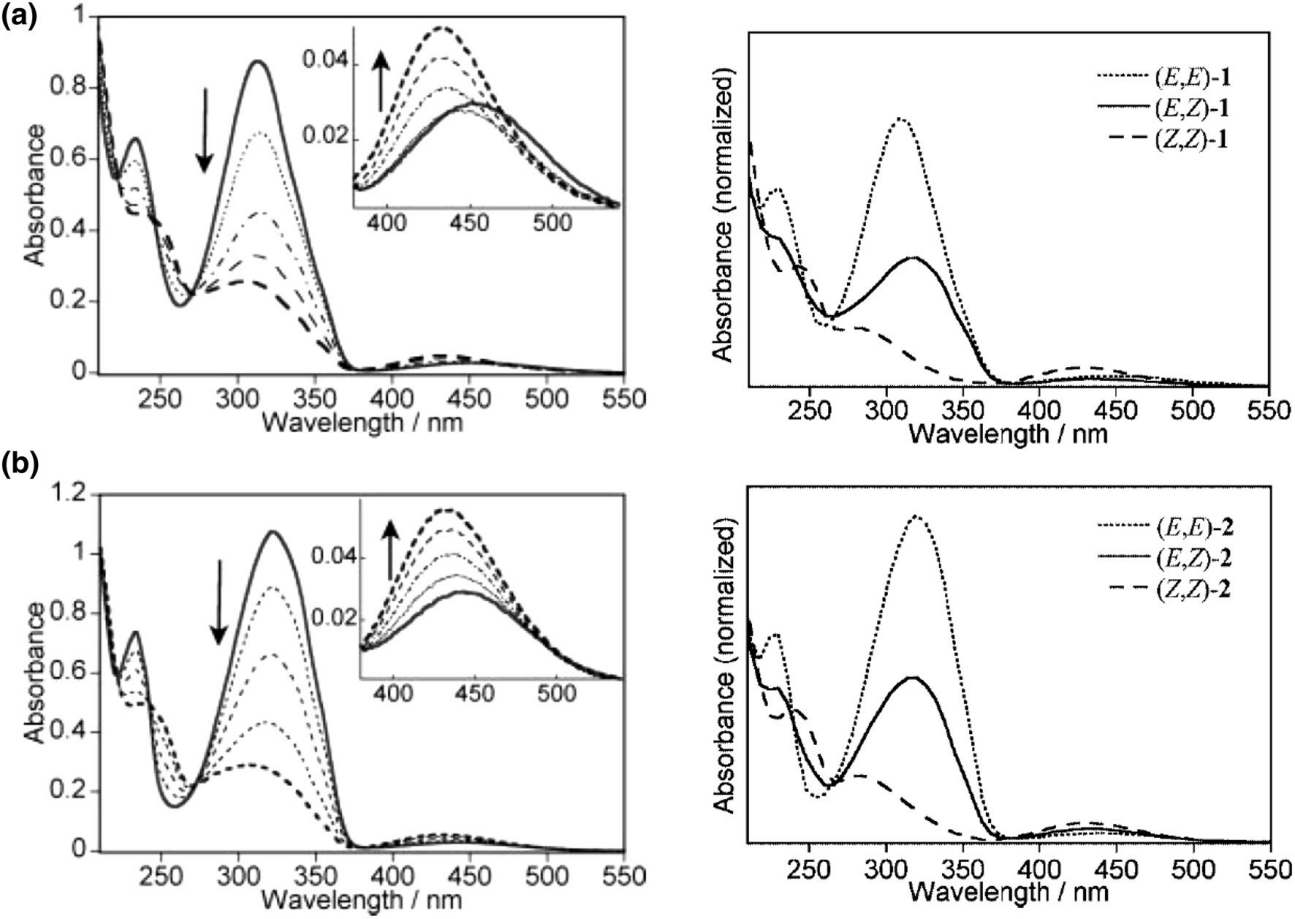
Left: changes in the absorption spectra of **a 6** and **b 5′** (Fig. 5) in acetonitrile upon irradiation at 313 nm. The insets show the n,π* band, spectral range (370–550 nm). Bold lines are the initial (solid) and final (dash) traces. Right: absorption spectra of each isomer of **a 6** and **b 5′** measured by use of a photodiode array detector attached to an HPLC system. Spectra are normalized at the isosbestic points (269 and 272 nm for **6** and **5′**, respectively). Numbers for compounds in reproduced material correspond to following numbers of compounds in this work: 1 = **6**, 2 = **5′**. Reprinted from [124]. Copyright 2006 with permission from John Wiley and Sons

The UV–Vis spectra of all-*trans* **6** macrocycle and all-*trans* isomer of acyclic dimer **5′** (Fig. 5a) in acetonitrile are compared in Fig. 6 [124]. The same Figure shows also changes upon irradiation with 313 nm wavelength light.

**Fig. 7:**
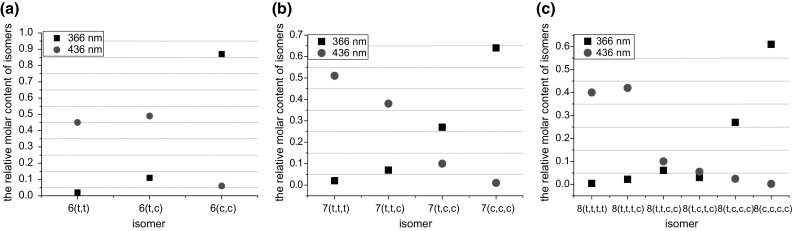
The ratios of isomers at the photostationary state (PPS) at various wavelength irradiation for **a 6** in acetonitrile, for **b 7** in chloroform and **c 8** in chloroform [123]

Photoisomerization of macrocycles **6**–**8** (Fig. 5) and acyclic compound **5′** from all-*trans*(*E*) to all-*cis*(*Z*) isomers proceeds gradually *via* respective *trans(E)*/*cis(Z)* isomers (depending on the number of azo groups). Comparison of UV–Vis spectra of macrocycle **6** *trans*/*trans*, and its acyclic analog **5′** is shown in Fig. 6 (left). Photoisomerization studies of all-*trans* isomers of compounds **6**–**8** showed that the ratio of all-*cis* isomers is irradiation wavelength dependent. The increase in quantity of *cis* azobenzene units upon irradiation at 366 nm and decrease at 436 nm was observed for **7** and **8** in chloroform. Similar behaviour was found for photoisomerization of **6** in acetonitrile. The ratios of isomers at the photostationary state for compounds **6**–**8** are schematically shown in Fig. 7a–c [123].

It was found that macrocyclic compounds **6**–**8** form complexes with alkali metal cations in methanol (determined by mass spectrometry, MS ESI). For all-*trans* isomers the highest peak in mass spectra was observed for cesium complexes; peak intensities for particular macrocycles can be ordered as: **6** > **7** > **8**. The observed trend was disturbed upon irradiation when *cis* isomers also participate in complex formation. It was explained by the softer character of *trans* isomers. However, the clear relationship: the intensity of peaks versus ion diameter in correlation with the size of macrocyle ring was not defined. It was concluded that other factors than only host–guest geometrical complementarity affect the binding strength of metal cations by azobenzophanes **6**–**8** [123].

**Fig. 8:**
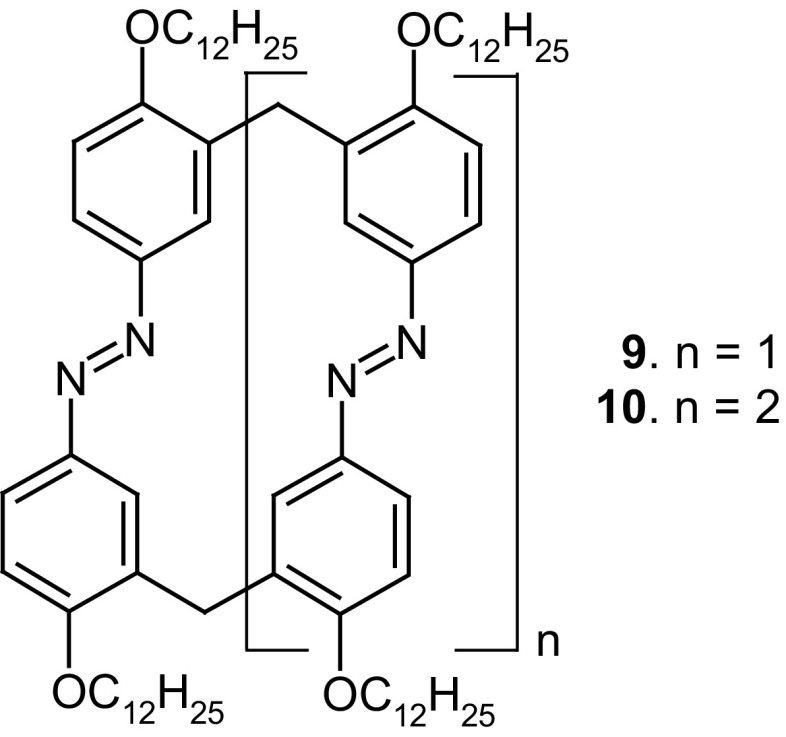
Azobenzophanes obtained by Norikane et al. [125]

Norikane et al. [125] obtained azobenzophanes **9** and **10** (Fig. 8), having structures similar to **6**–**8** (Fig. 5). The modification of the macrocyclic skeleton by attaching long alkoxyl chains resulted in photoresponsive liquid crystallinity.

**Fig. 9:**
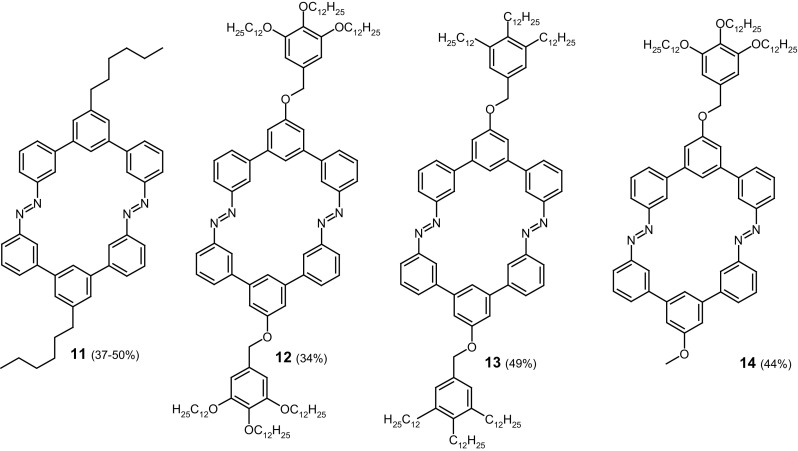
Bulky azobenzophanes synthetized by Mayor and co-workers [126]

The effect of different bulky substituents on the properties of azobenzophanes having the same macroring size was investigated by Mayor and co-workers [126]. Four *m*-terphenyl compounds **11**–**14** (Fig. 9) comprising different peripheral substituents were synthesized by multistep reactions, and different strategies, with the final step of reductive macrocyclization (LiAlH_4_, THF, r.t.) of the respective nitro compounds. Compounds **11**–**13** are symmetric opposite to derivative **14** with two different substituents at peripheral positions.

**Fig. 10:**
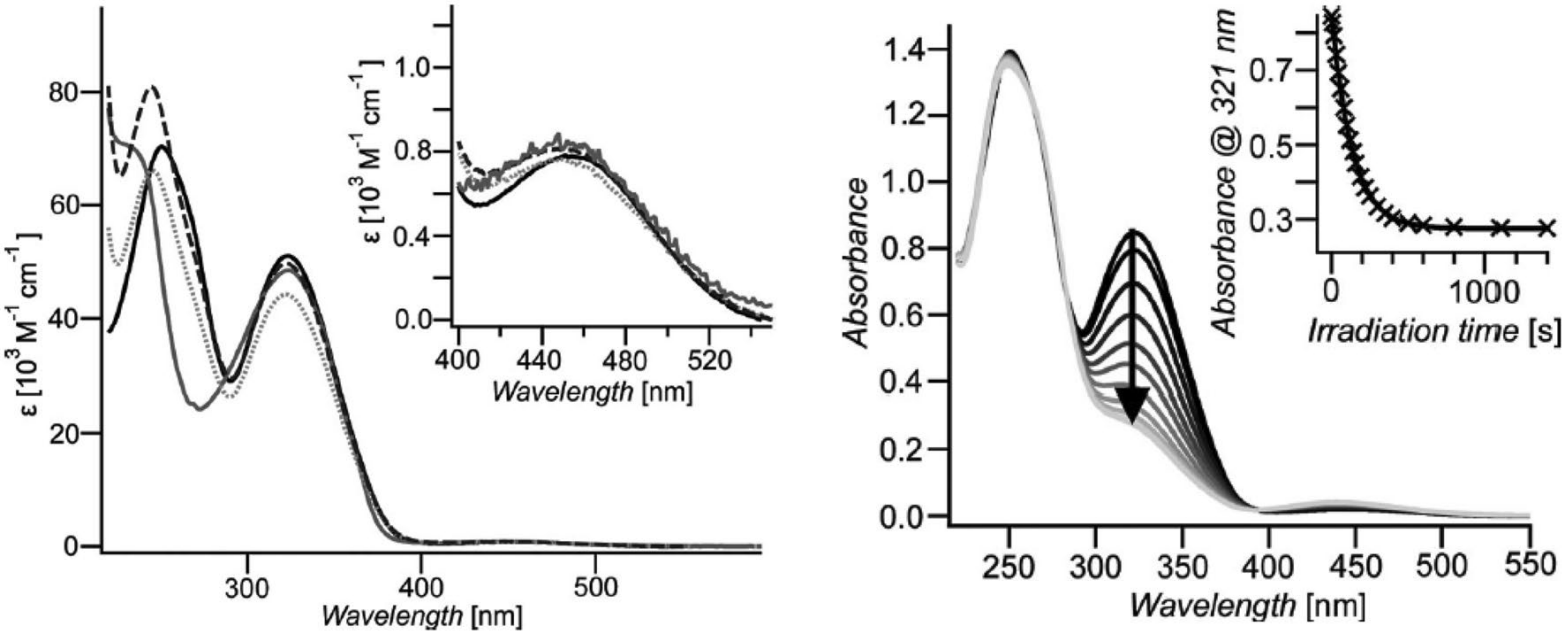
Left: absorption spectra of azobenzophanes **11**–**14: 11**-solid black line, **12**-dashed line, **13**-solid grey line, **14**-dotted line, in THF. Right: changes in absorption spectra of macrocycle **11** in THF upon irradiation at 313 nm. Inset: absorbance change versus irradiation time. Reprinted from [126]. Copyright 2009 with permission from John Wiley and Sons

The structures of obtained macrocycles were confirmed spectroscopically and the molecular weights of oligomers were determined by vapor pressure osmometry. UV–Vis spectra registered for macrocycles **11**–**14** in THF are shown in Fig. 10 (left) [126].

**Fig. 11:**
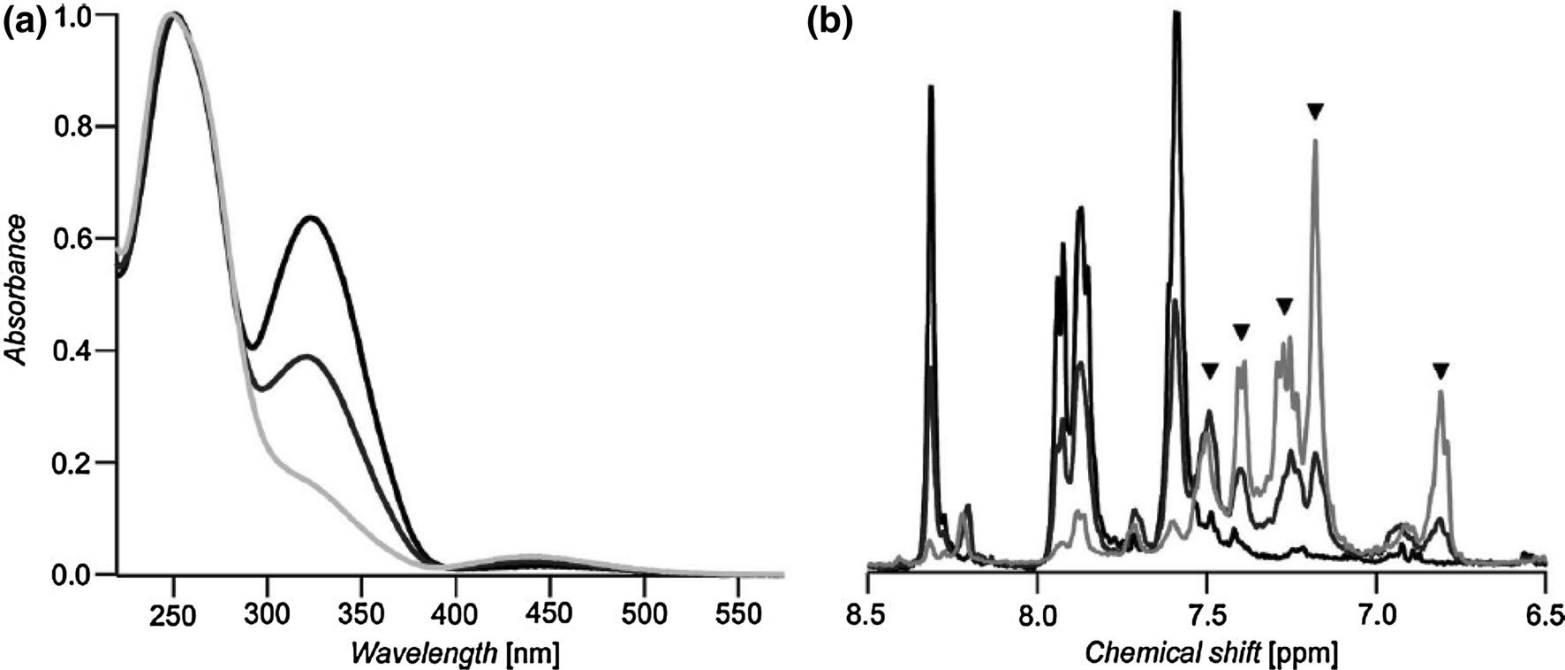
**a** UV-Vis spectra of macrocycle **11** showing the corresponding *E*/*Z* ratio (black: thermally stable state; dark grey: 50% isomerized; light grey: photostationary state). **b** Corresponding ^1^H NMR spectra (markers indicate the peaks corresponding to the *Z* isomer). Reprinted from [126]. Copyright 2009 with permission from John Wiley and Sons

Similar shape of spectra, i.e. π→π* ~350 nm and n→π* ~450 nm was found for all compounds, although below 300 nm in UV–Vis spectra of macrocycles **11**–**14** the differences are more pronounced. The blue shift of absorption bands for compounds **12** and **13** can be attributed to the effect of substituents on the central phenyl rings. UV–Vis spectra of **11**–**14** undergo changes upon illumination (313 nm, in THF). For all compounds comparable changes were observed, what is exemplified for **11** in Fig. 10 (right). The photostationary state was reached within 8 min. Photoisomerization is observable in UV–Vis spectra by the decrease of the π→π* and the increase in the n→π* absorption bands upon irradiation over time. These changes are associated with the formation of *Z* isomer. Photoisomerization was monitored by ^1^H NMR measurements along with UV–Vis experiments (Fig. 11). By integration of the corresponding ^1^H NMR signals the amounts of *E* isomer at the photostationary state was determined to be 15%. In all cases no intermediate *E,Z* isomer was observed as it was in the case of similar systems studied by Tamaoki [123]. This property can be attributed to the extremely rigid structure of macrocycles **11**–**14**. The back *Z*→*E* isomerization proceeds upon illumination or thermally. The *Z* isomers of the macrocycles **11**–**14** are stable pointing to very slow thermal back-reaction (the rate constant 1.15 × 10^−6^ s^−1^). The reversibility of the photoisomerization was investigated under illumination (450 nm). Contrary to the thermal-back process, under which macrocyles were fully converted back to *E* isomers, upon light stimuli ~ 15% remain in their *Z* form. However, this process seems to be reversible what was confirmed by experiments performed in several cycles.

**Scheme 3:**
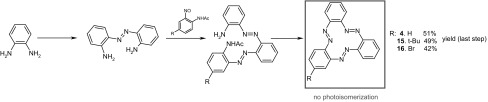
The general synthetic route for preparation of cyclotrisazobenzenes **4, 15** and **16** reported by Wegner and co-workers [127]

The effect of the strain in azobenzophanes on the photoisomerization of azobenzene unit is well seen in cyclotriazobenzenes, a special class of azobenzenophanes, in which all azobenzene units are conjugated. The simplest compound of this class has been already shown in Fig. 4b. Wegner and co-workers [127] prepared bromo- and *t*-butyl derivatives of the simplest cyclotrisazobenzene **4** using *o*-phenylenediamine as a substrate (Scheme 3).

Irradiation of **4, 15** and **16** (Scheme 3) showed no isomerization under various conditions. The unfavorable change of geometry upon possible photoisomerization should result in extreme strain in the macrocyclic skeleton, thus **4, 15** and **16** exist only as all-*E* isomers [cf.^122^]

**Fig. 12:**
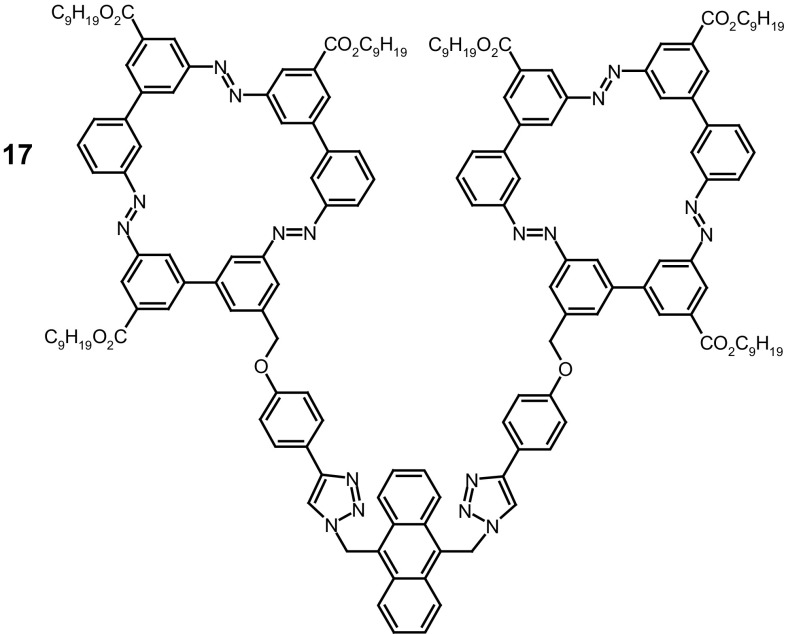
Macrocycle **17** described by Reuter and Wegner [128]

Light controlled sol–gel transition of azobenzene bismacrocycle **17** (Fig. 12) was described by Reuter and Wegner [128].

Due to significant π–π-stacking interactions macrocycle **17** forms 3D networks. Its gelation was observed in aromatic solvents, attributable to the incorporation of the solvent molecule inside the 3D π-stacking network. After UV irradiation at 365 nm the gel in *o*-xylene slowly liquefies as a result of dissociation of 3D network. The gel–liquid conversion of **17** upon irradiation till now is the first example of switchable 3D system which was controlled by two factors: incorporation of azobenzene units and non-covalent interaction, namely π-stacking of the azobenzene macrocyles. The proposed system can be potentially used in process where small molecules are released from the 3D network upon light stimulation.

**Fig. 13:**
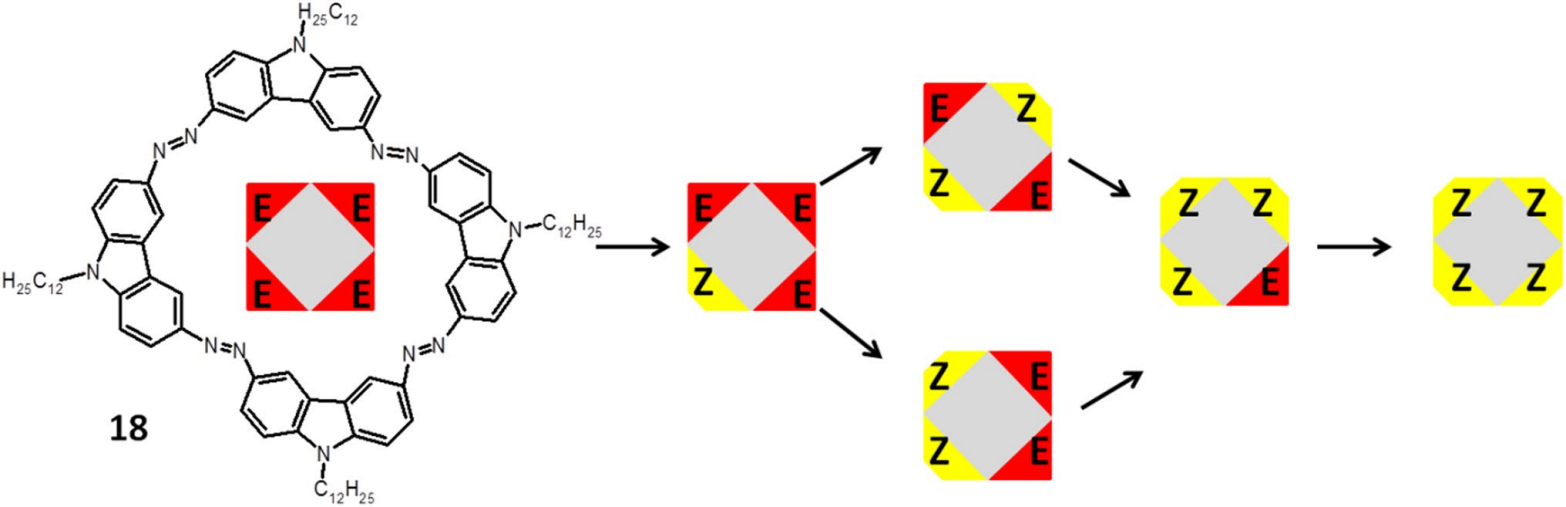
Macrocycle **18** with four azo moieties studied by Wegner and co-workers and combination of its possible *E*-*Z* isomers [129]. (Color figure online)

In photoswitchable cyclic azobenzenes several factors such as ring strain and the number of azo units are crucial for photochemical properties. These features depend also on rigidity and the position of linkers connecting the azobenzene units, the symmetry of the macrocycle and the degree of bonds conjugation. If at the beginning, i.e. before the illumination, a compound with several azo units is fully symmetrical in all-*E* configuration, the change of one of the azo groups into *Z* isomer affects the geometry of the macrocycle. The more azo units in macrocyle the more configuration variations (number of isomers) and geometrical changes can be expected. Wegner and co-workers [129] investigated the effect of symmetry changes on the photostationary state upon *E*→*Z* isomerization stimulated by both light and temperature. For this purpose they used macrocyle **18** with four azo moieties shown in Fig. 13. The isomerization of **18** was monitored by UV–Vis measurements and ^1^H NMR spectroscopy with in situ light irradiation. **18** in THF exists in the form of all-*E* isomer. Upon irradiation of this solution (125 μM) at 424 nm for 73 min. a mixture of five among six possible isomers was detected: the starting all-*E* (21%), *E,E,E,Z* (49%), *E,E,Z,Z* (19%), *E,Z,E,Z* (7%), and *E,Z,Z,Z* (4%). Under elevated (50 °C) temperature, at photostationary state, much higher ratio of the all-*E* isomer (55%) was detected, but lower quantities of *E,E,E,Z* (32%), *E,E,Z,Z* (4%) and *E,Z,Z,Z* (1.7%) isomers and almost unchanged amount of *E,Z,E,Z* (6.8%) isomer. It was concluded that at photostationary state the *E,E,Z,Z* isomer is favored over the *E,Z,E,Z* isomer. Comparison of the rates of thermal back isomerization reveals that the *E,Z,E,Z* isomer has the highest and the *E,E,Z,Z* isomer the lowest thermal stability. This can be ascribed to the ring strain of the particular forms. Different states can be achieved by the arrangement of the azo groups in macroring reflecting the overall symmetry of the molecule without introduction of additional substituents or applying different wavelength of the light used for illumination.

**Fig. 14:**
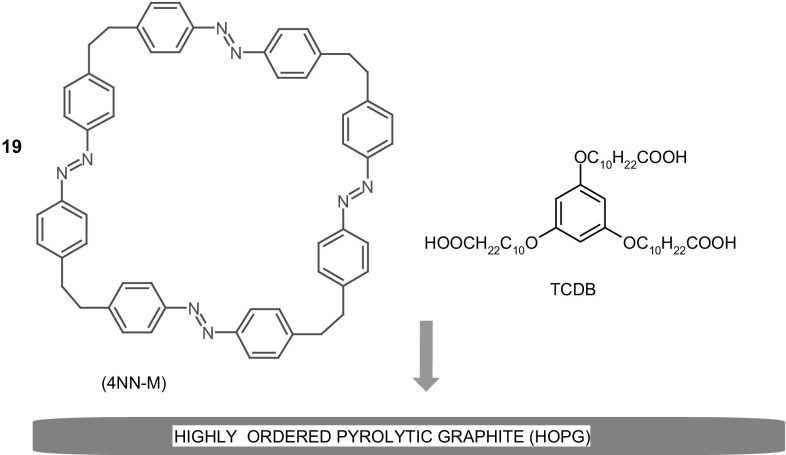
Photoresponsive system based on macrocycle **19** (4NN-M) and TCDB on HOPG surface [130]

Interesting, well organized system utilizing highly ordered pyrolytic graphite (HOPG) based on the photosensitive macrocycle bearing four azobenzene units **19** (4NN-M, Fig. 14) immobilized in the TCDB network was obtained and investigated by Wang and co-workers [130]. Upon UV illumination of the prepared material *E,E,E,Z* and *E,Z,E,Z* isomers are present at photostationary state. The proposed methodology was found to be useful for fabrication of nanostructures and can be valuable for production of photosensitive nanodevices.

**Fig. 15:**
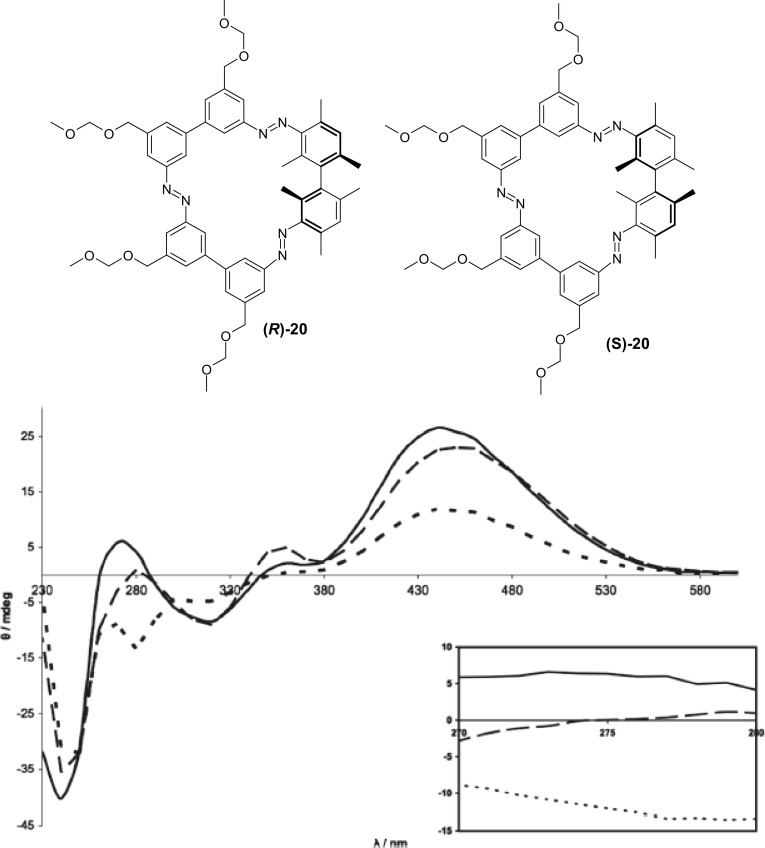
Top: (*R*)-**20** and (*S*)-**20** enantiomer of bismesitylcyclotrisbiphenyl macrocyle obtained by Reuter and Wegner [131]. Bottom: CD spectra at different photostationary states at 302 nm (solid line), visible light (dashed line), and 365 nm (dotted line) (5.9 × 10^−5^ M), with an enlarged graph for the region 270–280 nm (inset). Reprinted with the permission from [131]. Copyright 2011 American Chemical Society

A ternary switch utilizing chiral macrocycle was presented by Reuter and Wegner [131]. They obtained both enantiomers *R* and *S* and racemic form of chiral bismesitylcyclotrisazobiphenyl compound **20** (Fig. 15) in about 40% yield.

Three different photostationary states were gained by irradiation of **20** with different UV (302 and 365 nm) and visible light. The photoisomerization was investigated by CD spectroscopy. A large increase in the optical rotation angle for (*S*)-**20**: [α]^20^_D_ = 2128° and for (*R*)-**20**: [α]^20^_D_ = − 2077° in comparison with acyclic 3,3′-diaminobismesityls comes from the helical shape of macrocyclic compounds. All *E*-isomer was obtained by heating samples of (*S*)-**20** and (*R*)-**20** at 45 °C overnight. CD spectra of two all-*E* enantiomers are mirror images with four different absorption maxima. Upon irradiation of all *E*-isomer of (*S*)-**20** with three different wavelength the photostationary state was reached after ~ 15 min. For (*S*)-**20** seven different isomers were detected by ^1^H NMR measurements: six species being different *E*/*Z* isomers (one (*E,E,E*), two (*E,E,Z*), two (*E,Z,Z*), and one (*Z,Z,Z*)). The seventh one was described as a stable conformer of the (*E,E,Z*)-isomer with azo bond next to the bimesityl unit in *Z*-form. The different ratio of these isomers at particular photostationary state is manifested in CD spectra that varied mostly in intensities, but with preserved similar overall shape. However, a difference can be observed at 275 nm, when irradiating sample with mentioned above three different wavelengths: at 302 nm—positive value, visible light—no dichroism, at  365 nm negative value what is promising for ternary switch with +, − and 0 output (Fig. 15, bottom).

## Crown ethers with azobenzene moiety(-ies)

**Fig. 16:**
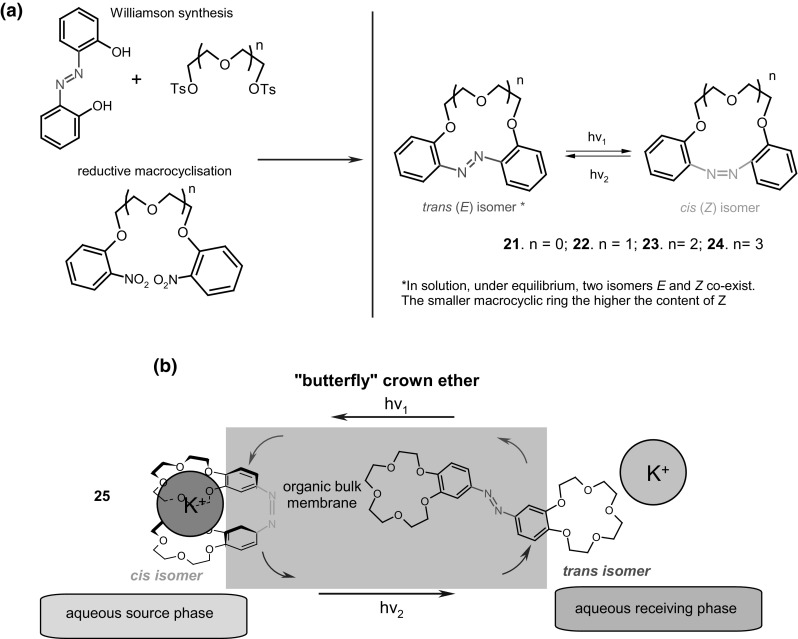
**a** Crown ethers **21**–**24** with inherent azo group (Takagi’s and Biernat’s group) [132–140] (b) **25 -** azobis(benzo-15-crown-5) an example of butterfly crown ethers obtained and studied by Shinkai [141, 142]

Azobenzene unit incorporated into crown ethers skeleton was first reported by Takagi and co-workers almost 40 years ago [132, 133]. Azo bearing crowns **21**–**24** (Fig. 16a) were obtained by Williamson reaction from dihydroxyazobenzene and alkylating agents. The synthesis of this type of compounds (**21**–**23**, Fig. 16a) was also elaborated in details by Biernat and co-workers [134–140]. Reductive macrocyclization of dinitropodands allowed the preparation of vast number of macrocyclic compounds showing diverse properties. By this method azoxycompounds are formed next to azocompounds. They were studied e.g. as ionophores in ion-selective membrane electrodes and chromogenic agents for metal cation complexation. At first glance—these simple compounds bring a great potential in supramolecular chemistry not only as metal cation complexing properties, but also due to photosensitivity. There are also known crown ethers with azo group located at the periphery of the molecule with brilliant example of so called “butterfly crown ethers” obtained and investigated by Shinkai et al. [141, 142]. These photo-switchable compounds were used for light-driven transport of potassium and sodium. Figure 16b shows the scheme of light-driven transport of potassium cations across organic bulk membrane with the use of photoresponsive azobis(benzo-15-crown-5) **25**.

These early works on azo group bearing crown ethers inspired further development of synthetic methods, challenging functionalization, and studies (both experimental and theoretical) of properties and finally applications of macrocyclic polyethers.

### Crown ethers with inherent azobenzene group(s)

**Fig. 17:**
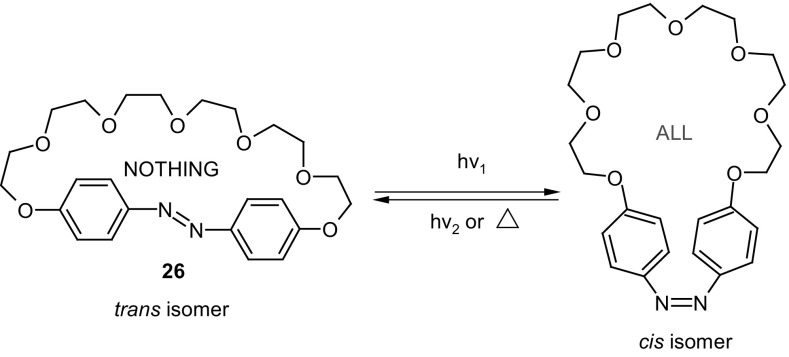
Example of “all-or nothing” photoswitchable crown ether **26** [141, 143]

Among the first synthesized crown ethers with azo unit incorporated into the macrocycle were so called “all or nothing” crown ethers exemplified by **26** (Fig. 17) obtained by Shinkai et al. [141, 143]. These photoswitchable compounds form complexes with metal cations with affinity that depends on the geometry of azo group. The *cis* isomer obtained by illumination binds cations, whereas in the dark the cation is released due to decreasing the cavity size being a consequence of isomerization to *trans* form. The spectral behavior of “all or nothing” crowns of different size of the macrocycle and their ability to form complexes with alkali metal cations was later studied theoretically using density functional theory (DFT) [144]. The results showed good agreement between experimental and computational attempts.

**Fig. 18:**
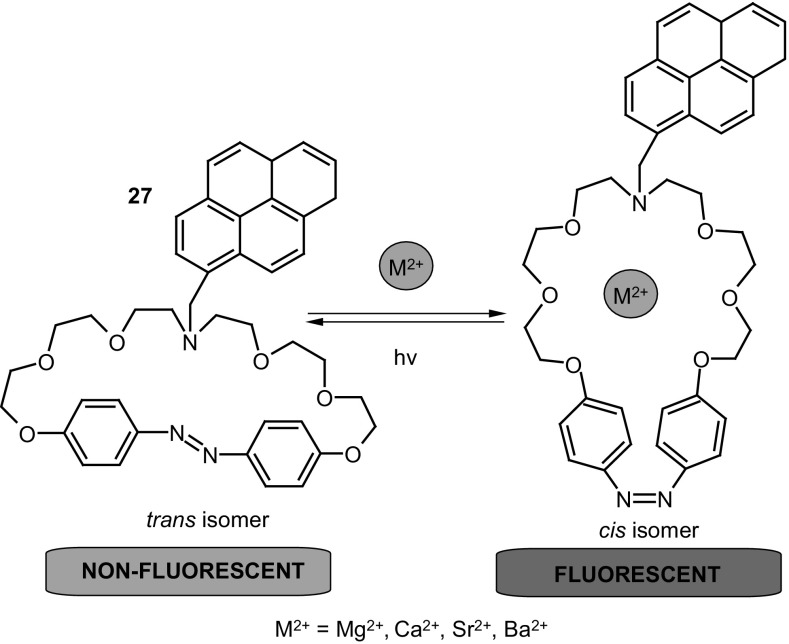
Azobenzene embedded *N*-(11-pyrenyl methyl)aza-21-crown-7, **27** studied by DFT by Wang and co-workers [145]

Computational methods were also used by Wang and co-workers [145] to study *trans*-azobenzene embedded *N*-(11-pyrenyl methyl)aza-21-crown-7 **27** (Fig. 18) as a fluorogenic receptor for alkaline-earth metal cations.

According to density functional theory using B3LYP/6-31G(d) it was determined that the ether chain of *trans* isomer of the compound becomes almost a straight line forming a strip crown ring. Calculated structure of *cis* isomer shows cavity enables coordination of metal cation inside the macrocycle. The optimized structures of complexes of the host molecule and alkaline earth metal cations (Mg^2+^, Ca^2+^, Sr^2+^, and Ba^2+^) indicate that the ligand binds calcium cations the strongest due to the best match of ion radius to the cavity size. These results showed, that proposed system can act as molecular device of double function.

**Fig. 19:**
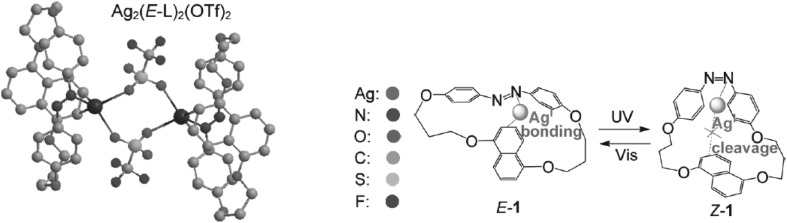
Left: crystal structure of dimeric Ag^I^ complex *E*-**28**. Right: schematic illustration of photoresponsive cleavage/binding of cation-π bond. Numbering of compound in the reproduced material corresponds to number of compound in this work: 1 = **28**. Adapted with permission form [146]. Copyright 2010 American Chemical Society. (Color figure online)

Tamaoki and co-workers [146] studied the effect of *trans*–*cis* isomerization of [5.5](4,4′)azobenzeno(1,5)naphthalenophane **28** (Fig. 19) on silver(I) complexation. The resolved crystal structure of 1:1 complex showed that two silver cations are complexed to form dimeric structure with azobenzenonaphthalenophane in *trans* form (Fig. 19 left).

^1^H NMR studies showed that complexation of silver cation is controlled by reversible *trans*–*cis* isomerisation of azo moiety; photoisomerization of *trans* to *cis* isomer causes the cleavage of the π–cation interaction. The opposite change was found under reverse isomeriation (Fig. 19, right).

**Fig. 20:**
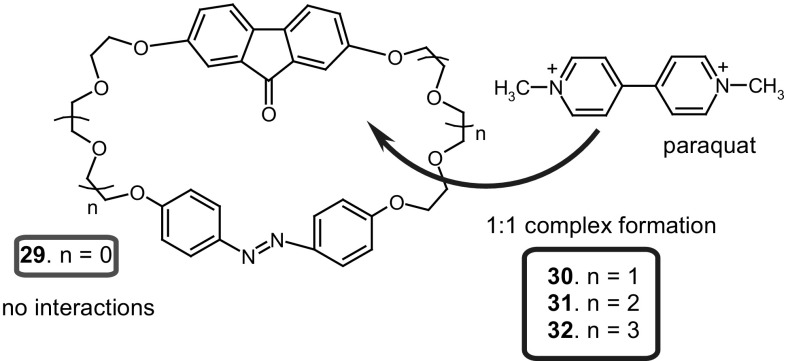
Crownophanes **29**–**32** bearing 2,7-dioxyfluorenone and 4,4′-azobiphenoxy groups synthesized and studied by Kirichenko and co-workers [147] showing binding ability of dimethylbipyridinium (paraquat) bis(hexafluorophosphate)

Kirichenko and co-workers [147] described synthesis and complexing properties of four crownophanes **29**–**32** (Fig. 20) containing 2,7-dioxyfluorenone and 4,4′-azobiphenoxy groups joined with di-, tri-, tetra-, and pentaethylene glycol moieties. Based on NMR, UV–Vis, and X-ray data it was concluded that all macrocycles exist in solution and in solid state in *trans*-configuration of azobenzene unit. The *trans* to *cis* isomerization of **30** can be achieved by UV-light (365 nm) irradiation. Macrocycles **30**–**32** bind 4,4′-dimethylbipyridinium (paraquat) bis(hexafluorophosphate), an electron-deficient model compound. The derivatives of this compound are used in synthetic procedures leading to interpenetrating complexes (pseudorotaxanes). Complex formation of paraquat with macrocyles is based on π–π interactions between π-donor aromatic moieties of cyclophanes and π-acceptor dipyridinium core of the guest. ^1^H NMR and MS measurements showed the formation of 1:1 inclusion complexes of pseudorotaxane type. The stability of complexes changes in the order: **31** > > **30** > **32**. The smallest macrocycle **29** does not complex the guest due to lack of complementarity between size of the guest and cavity of the host.

Described by Takagi’s and Biernat’s groups 13- and 16-membered crown ethers **22** and **23**, as it was stated earlier, form complexes with metal cations. The X-ray structure of complexes of 13-membered crown with lithium bromide [148] and sodium iodide [149] were described. Metal cation complexes of larger, 16-membered crowns were also obtained. In solid state 16-membered crown forms sodium complex of 1:1 stoichiometry [150] while with potassium salt sandwich type 2:1 (crown:ion) complex [151] is created. In all cases the azo group is in *trans* configuration. It was also shown that the analysis of crystal structures of complexes of azobenzocrown ethers with alkali metal cations can be helpful in interpretation of the selectivity of ion-selective electrodes doped with particular macrocyle [152].

**Fig. 21:**
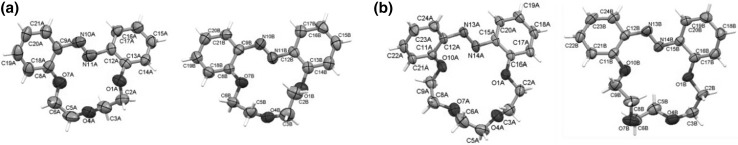
ORTEP view of **a** crown **22** (molecules **22**A and **22**B), **b** crown 23 (molecules **23**A and **23**B). In both cases the thermal ellipsoids are drawn at the 50% probability level. Reprinted from [153]. Copyright 2008 with permission from Elsevier. (Color figure online)

The X-ray structures of uncomplexed *trans* isomers of crown **22** and **23** were also investigated [153]. In the unit cells there are two independent molecules **22**A and **22**B or **23**A and **23**B (Fig. 21).

The kinetics of the buildup and decay of photoinduced birefringence of crown ethers with inherent azo groups **21**–**24** (Fig. 16a) of different size of the macrocyle was investigated in poly(methyl methacrylate) matrix [154]. For all cases it was found that the kinetics of the buildup of the birefringence was suitably described by a sum of two exponential functions, the time constants (being function of the pumping light characteristic) and sample thickness. The dark decays were described the best by the stretched exponential function, with the characteristic parameters (time constant and stretch coefficient) being practically independent of the type of crown ether. The time constants of the signal decay were orders of magnitude shorter than the respective constants of the dark isomerization of the azo crown ethers. Thus it indicates that the process controlling the decay was a relaxation of the polymer matrix and/or a rearrangement of the flexible parts of the crowns.

**Fig. 22:**
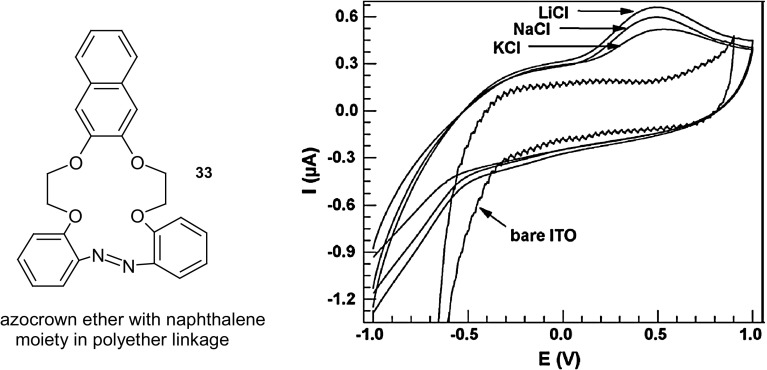
16-membered crown **33** and voltammograms for 22-monolayers LB films on ITO, based on this macrocyclic compound, electrode in solutions of KCl, NaCl and LiCl (0.1 M) registered with a sweeping rate of 50 mVs^−1^. Reprinted from [155]. Copyright 2009 with permission from Elsevier

The introduction of the azo group into compounds results not only in photoresponsive but also redox active properties. An example can serve 16-memebered crown **33** (Fig. 22) [155] with naphthalene joined by two oxyethylene chains. This macrocycle was used for the preparation of Langmuir–Blodgett (LB) film deposited onto solid ITO substrate. The complexation of metal cations on these electrodes can be successfully observed by cyclic voltammetry (CV). Figure 22 shows CV obtained for a 22-monolayers LB film on an ITO electrode in solutions of KCl, NaCl and LiCl (0.1 M). Bare ITO shows no redox peaks in the presence of K^+^, Na^+^ or Li^+^ ions. For the LB film based on crown **33** film, an electrochemical response in the presence of metal salts was observed. The change of observed signal was attributed to the specific interactions between the film and the metal ions. The peaks in voltammograms can be ascribed to the electro-reduction of the azo moiety to the hydrazo group, which consumes two electrons and two protons according to the overall reaction. The strongest effect was observed in the presence of lithium cation, showing the possibilities of its electrochemical sensing.

**Fig. 23:**
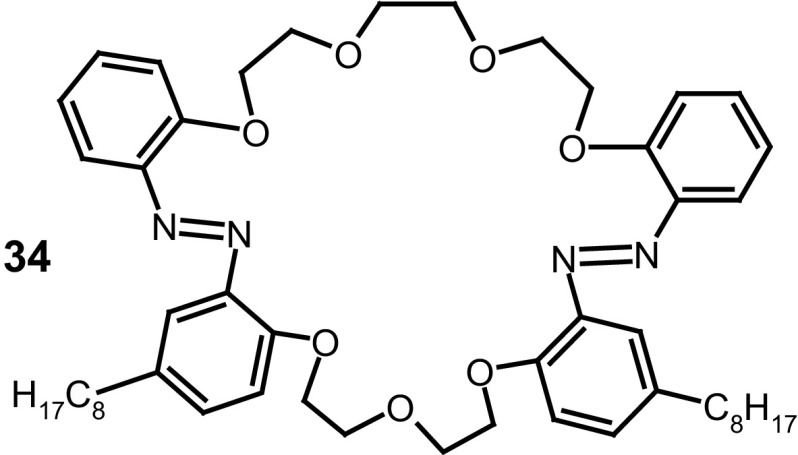
29-Membered diazocrown **34** showing electrochemical response towards potassium cations [156]

Similar experiments were performed for a number of macrocyclic compounds, e.g. larger 29-membered macrocyle **34** (Fig. 23), bearing two *n*-octyl substitutents in benzene rings and two azo groups as a part of macrocycle [156]. Langmuir–Blodgett (LB) and physical vapor deposition (PVD) films on ITO showed electrochemical response towards metal cations. Cyclovoltamperometric curves registered for LB films of 29-membered compound **34** point out that among alkali metal cations Li^+^, Na^+^ K^+^, potassium ion was preferentially complexed under applied conditions suggesting the best host and guest size complementarity.

**Fig. 24:**
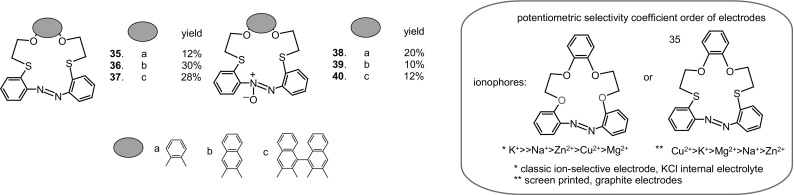
Left: Thiaazo- (**35**–**37**) and thiaazoxy (**38**–**40**) crown ethers obtained by Kertmen and Szczygelska-Tao. Right: comparison of the trend of potentiometric selectivity coefficients of electrodes with crown **36** and its oxygen analog shown is in a frame [157]

The selectivity of crown ethers and other host molecules towards metal cation can be controlled also by changing the type of donor atoms. 16- and 18-membered azo- and azoxythiacrown (forming next to azo compounds) ethers **35–40** (Fig. 24, right) were obtained in satisfactory yields by Kertmen and Szczygelska-Tao [157] using reductive macrocyclization procedure. Thiacrowns were tested as ionophores in ion-selective, graphite screen printed electrodes. Opposite to their oxygen analogs, sulfur containing compounds preferentially supposed to form complexes with softer metal cations. All electrodes doped both with azo- and azoxythiacrowns **35–40** (Fig. 24) showed high sensitivity towards heavy metal cations. The effect of softer sulfur donor atom in the skeleton of macrocycles on the response of ISE with membrane doped with **35–40** can be visualized by comparison of the order of potentiometric selectivity of thia-crown and its oxaanalog [137], shown in Fig. 24 (right, in a frame).

**Scheme 4:**
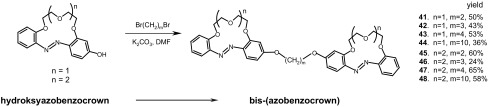
Synthetic route for preparation of bis-(azobenzocrown)s **41**–**48** from hydroxyazobenzocrowns as substrates [162]

Potassium selectivity of electrodes based on derivatives of 16-membered crown ether **23** was well-proved over years of working with ISEs. 13-membered azobenzocrowns, derivatives of compound **22** (Fig. 16a) are sodium ionophores [134, 136–138, 158–160]. To improve the characteristic of the sodium and potassium sensors, important for clinical analyses, new derivatives of both 13- and 16-membered crowns were prepared and at the same time new technical solutions, including miniaturization of the sensors, were applied. Recently, a series of bis-(azobenzocrown)s (compounds **41**–**48**, Scheme 4) based on the skeleton of parent 13- and 16-membered crowns **22** and **23** (Fig. 16a) linked by α,ω-dioxaalkane chains between two macrocycles have been obtained [162]. Bis-crowns were synthesized from the respective hydroxyazobenzocrowns obtained in reaction analogous to Wallach rearrangement elaborated by Luboch [161].

**Fig. 25:**
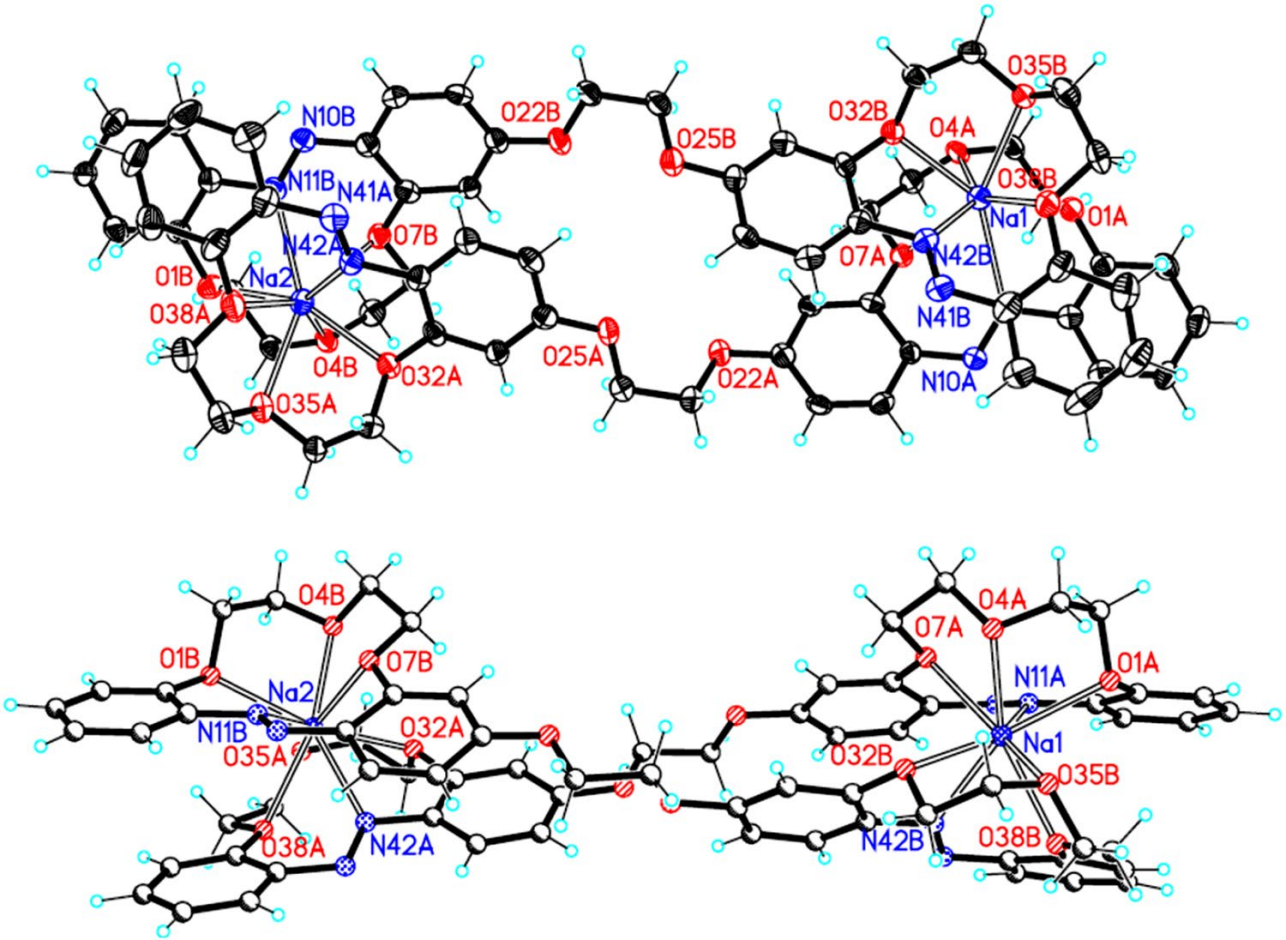
Two projections of macrocyclic cation [Na_2_(trans-**41**)_2_]^2+^ in **41**-NaI complex with a partial labeling scheme. Reprinted from [162]. Copyright 2012 with permission from Elsevier

The unique structure of intermolecular of 2:2 stoichiometry sandwich-type complex of bis-(azobenzocrown) **41** with sodium iodide was obtained [162]. It is presented in Fig. 25.

Bis-(azobenzocrown)s **41**–**48** were used as ionophores both in classic and miniature, all-solid state, screen-printed, graphite ion-selective electrodes. New sodium and potassium sensors feature by short response times, stable potential and high selectivity, in particular high K/Na selectivity.

Bis-(azobenzocrown)s **41**–**48** form complexes with metal cations also in acetonitrile. The increase of stability constant values comparing analogous monocrown bearing alkoxy substituent proves beneficial effect of the presence of two binding sites in one molecule.

**Scheme 5:**
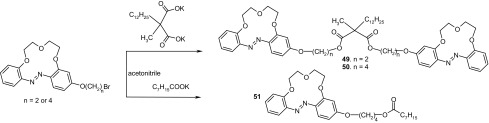
Synthesis of bis-(azobenzocrown)s **49** and **50**, diesters of dodecylmethylmalonic acid and monoazobenzocrown **51** [163]

Another example of biscrowns are diester derivatives of dodecylmethylmalonic acid joining two 13-membered azobenzocrown moieties obtained in Luboch group [163] (compounds **49** and **50**, Scheme 5). Biscrowns were obtained using bromoalkoxy derivatives of azobenzocrowns [164] and potassium salt of dodecylmethylmalonic acid in ~ 40% yield. For comparative studies monoester derivative **51** was synthesized.

**Fig. 26:**
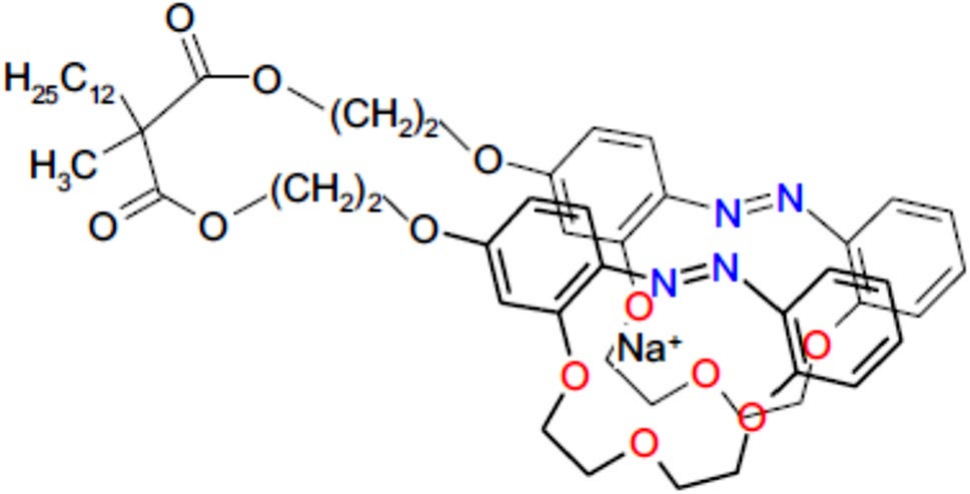
Proposed organization of biscrown **49** sodium cation complex. Reprinted without changes from [163]. Copyright 2016 with permission from Springer Publishing Company (http://creativecommons.org/licenses/by/4.0/)

For biscrowns **49** and **50** three isomers *trans*–*trans, trans*–*cis* and *cis*–*cis* can be considered. From ^1^H NMR spectra registered in d-acetone it was found that in solutions of **49** and **50** *trans*–*trans* and *trans*–*cis* isomers dominate representing altogether ~ 90% of the total amount of compounds. The presence of *cis*–*cis* isomer of **49** was observed upon irradiation with UV light. For monoester derivative **51** the ratio of *trans* to *cis* isomer was evaluated as 6:4. *Trans*–*trans* and *trans*–*cis* isomers of **49** and especially of **50**, differ significantly in TLC properties. This can be associated with different complexation properties of both isomers [166]. *Trans* isomers of azobenzocrowns show higher affinity towards metal cations than *cis* forms. Thus *trans*–*trans* isomer is probable able to form intramolecular sandwich type complexes (Fig. 26) with metal cations whereas for *trans*–*cis* isomer rather intermolecular complexes are expected. This hypothesis finds confirmation in previously published works of the above authors and in articles published by other groups [149, 166, 167].

Formation of sodium complex by *trans*–*trans* isomer of **49** was confirmed also by ^1^H NMR measurements. Stability constant value of (1:1) complex of **49** in acetone was estimated as logK ~ 3.0 from UV–Vis titrations.

**Fig. 27:**
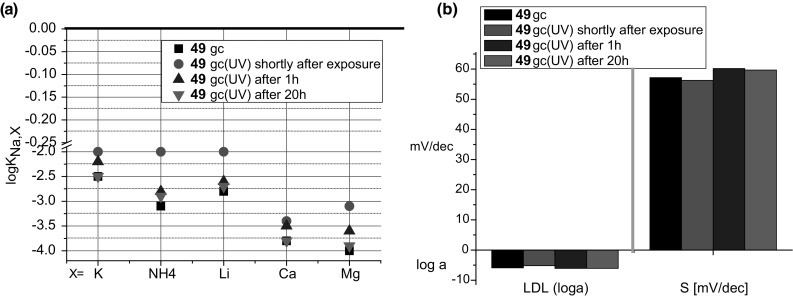
**a** Selectivity coefficients (SSM, 0.1M) and **b** potentiometric response characteristics: LDL [loga] and slope [mV/dec] for glassy carbon sodium selective electrodes based on **49** as ionophore. UV-electrode upon irradiation with UV light (9W) [163]. (Color figure online)

Bis-crowns **49** and **50** based on 13-membered rings, were tested as sodium ionophores in classic and miniature, solid contact: screen-printed and particularly glassy carbon membrane ion-selective electrodes. Plasticizers 2-nitrophenyl octyl ether (*o*-NPOE) and more lipophilic di(2-ethylhexyl) sebacate (DOS) can be successfully used for bis(azobenzocrown) containing membranes. It was proved that possible isomerization under usual conditions does not significantly affect the characteristics of the prepared electrodes. The influence of UV irradiation on the properties of glassy carbon electrode with ionophore **49** is shown in Fig. 27. After exposition to UV light (1 h, 365 nm), the electrode regains its properties practically after  2 h conditioning in NaCl solution.

**Fig. 28:**
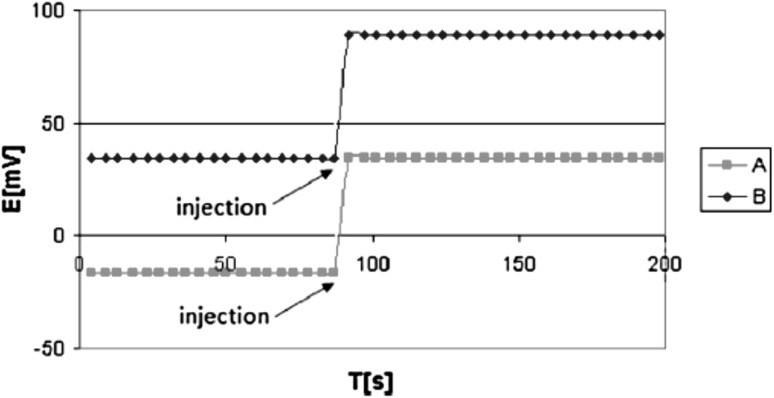
Response time of glassy carbon electrode with membrane with ionophore **49** (*o*-NPOE as plasticizer) A 0.9 mL of NaCl solution (0.1M) was injected to 100 mL of NaCl solution (10^−4^ M), B 0.9 mL of NaCl solution (1M) was injected to 100 mL of NaCl solution (10^−3^ M). Reprinted without changes from [163]. Copyright 2016 with permission from Springer Publishing Company (http://creativecommons.org/licenses/by/4.0/). (Color figure online)

Electrodes with the tested biscrowns **49** and **50** were found to have better selectivity coefficients K_Na/K_ than the electrodes with the monocrown **51**. The best selectivity coefficient Na/K was achieved for the screen printed graphite electrode with the addition of carbon nanotubes into the membrane (**50** as the ionophore, logK_Na,K_ = − 2.6). No significant differences were also observed between the selectivities of the classic and solid contact electrodes. In the last case lower detection limits (LDL) may be obtained. The membrane doped with carbon nanotubes deposited onto graphite screen-printed electrodes results in the better potential stability, detection limit and selectivity of biscrown-based electrodes. The electro-conductive material was introduced directly into the membrane in a manner analogous to that proposed by Ivaska and co-workers [168]. For glassy carbon electrodes to improve the conductivity, between the membrane and glassy carbon the conductive PEDOT/PSS polymer blend was introduced by electropolymerization. Such electrodes have better (lower) LDL than plain glassy carbon electrode. Electrodes with ionophores **49** and **50** characterize with response times not longer than 10 s, illustrated in Fig. 28 for membrane electrode doped with **49**.

**Fig. 29:**
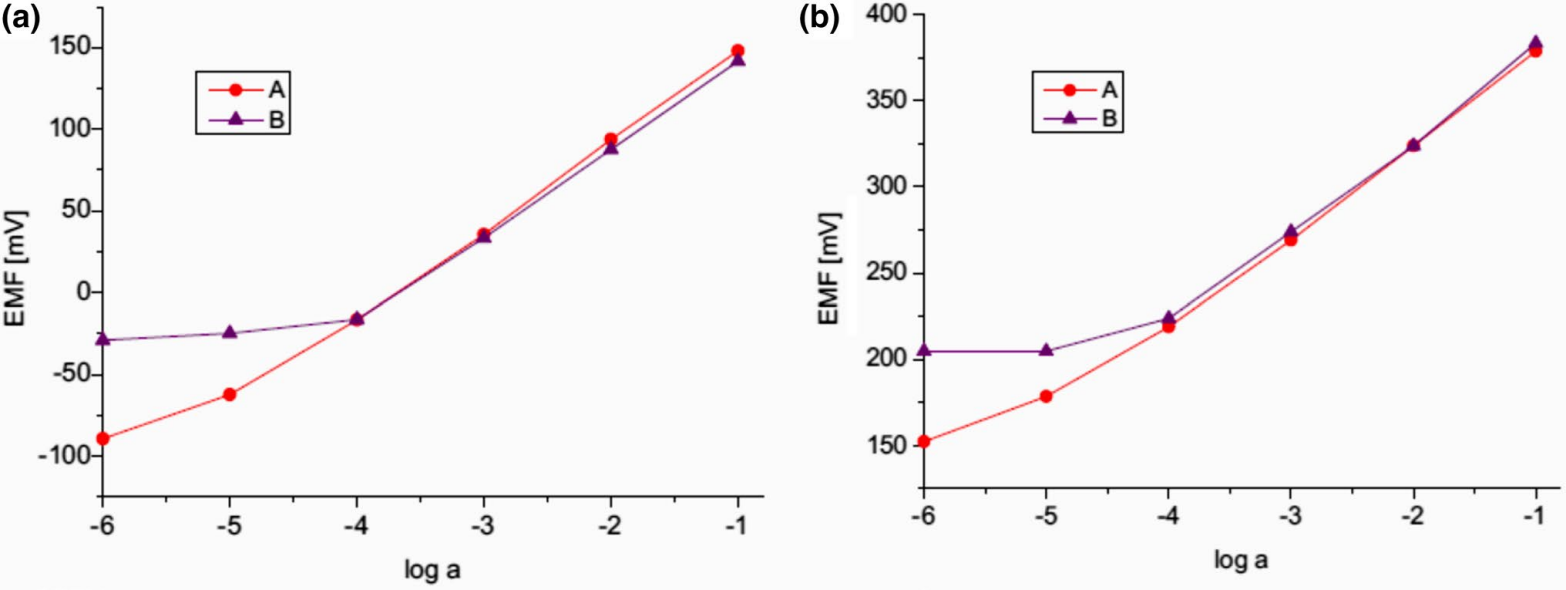
Response curves for Na^+^ obtained with ISEs based on ionophre **50 a** graphite screen-printed electrode **b** glassy carbon electrode. Curve A indicates the response for Na^+^ without and curve B Na^+^ in the presence of interfering ions (4.2 mM K^+^, 1.1 mM Ca^2+^, 0.6 mM Mg^2+^). Reprinted without changes from [163]. Copyright 2016 with permission from Springer Publishing Company (http://creativecommons.org/licenses/by/4.0/)

Electrodes based on **49–51** characterize by stable potential in a wide range of pH, depending on the type of the used plasticizer, e.g. electrodes with compound **49** and DOS show stable potential in the pH range 2–10 (0.1M NaCl). Proposed sodium sensor (based on **50**) fulfills requirements for electrodes used in clinical analysis [169]. The response of electrodes based on **50** for sodium in the presence of interfering metal cations corresponding to their blood plasma levels are shown in Fig. 29.

The electrodes were tested for sodium in blood plasma giving consistent results with independent measurements carried out in clinical analytical laboratory.

### Crown ethers with peripheral azo group

The interactions between photoswitchable azobis-(benzo-18-crown-6) and alkaline earth metal cations were studied by DFT and reactive molecular dynamics (reactive MD) by Pang et al. [170]. Optimized structures of complexes revealed that in the case of Ba^2+^ complex the distance between two cations is the largest among tested complexes in their *trans* form, and the shortest among *cis* complexes. Macrocycles become face-to-face when complexing Ba^2+^ ions. Small energy difference between Ba^2+^ complex in its *trans* and *cis* form indicates facile *cis* to *trans* thermal conversion. Calculation the Ba^2+^ complex allows to conclude that it is a suitable candidate for photocontrolled catalysis.

**Fig. 30:**
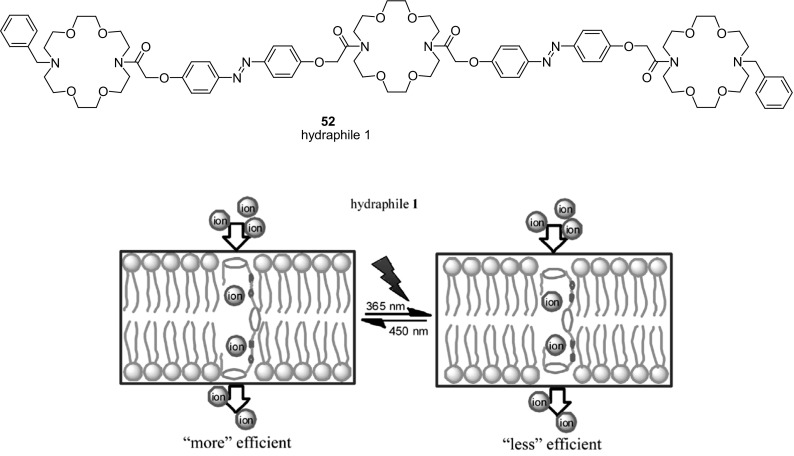
Tris(macrocyle), amphiphilic azobenzene moiety bearing compound **52** - hydraphile 1 (top) and schematic presentation of transmembrane ion transport in photoswitchable system based on hydraphile. Reprinted from [171]. Copyright 2015 with permission from Elsevier

To mimick the structure and function of biological ion channels the light-regulated transmembrane system was proposed by using tris(macrocycle) system based on diaza-18-crown-6 joined by azobenzene photoswitchable moieties **52** (hydraphile 1, Fig. 30) [171]. The liposome-based ion transport assays revealed that compound **52** displays an efficient transmembrane activity with Y_max_ around 0.7 at 40 μmol/L of **52** in DMSO. Due to the presence of azobenzene moieties the potassium ion transport by the molecule across bilayer membranes can be regulated by applaying of external source of light. The photoisomerization of azo groups induces changes of transmembrane length of the ion channel and this way regulating the efficiency of the ion transport.

**Fig. 31:**
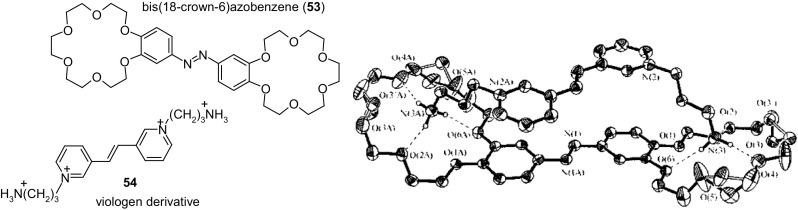
Structure of bimolecular complex of bis(18-crown-6)azobenzene **53** with viologen derivative **54**. Reproduced from [172]. Copyright 2008 with permission from Springer Publishing Company

In many chemical and photochemical processes donor–acceptor complexes (D-A complexes) play an important role. Such systems are also investigated as organic conductors and photoconductors that find applications in nonlinear optics. D–A complexes of a series of bis(crown)stilbenes, and also of bis(crown)azobenzene with salts of alkylammonium viologen derivatives were studied in solution and in a solid state by Gromov and co-workers [172]. X-ray structure of complex of bis(18-crown-6)azobenzene **53** (Fig. 31) with viologen derivative **54** showed that the central parts of donor and acceptor molecules feature planar geometry. The proposed systems can be used for the design of optical sensors and molecular devices.

## Colorimetric and spectrophotometric ion receptors

**Scheme 6:**
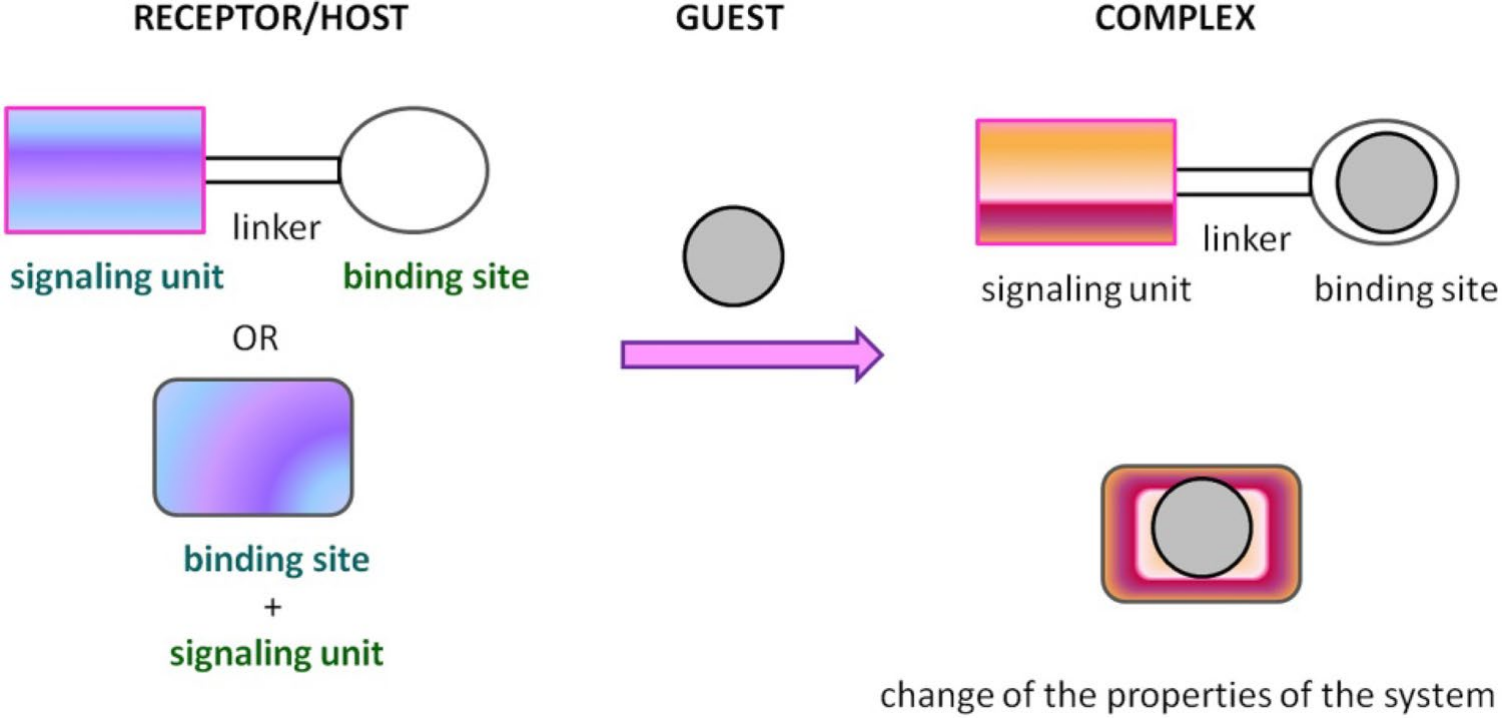
Schematic: the idea of chromo- and fluorogenic molecular receptors

Molecular recognition can be utilized in many branches of science and technique if the information about host–guest interaction could be converted into analytically useful signal, e.g. optical or electrochemical. Optical signaling in the visible range of the electromagnetic spectrum draws special attention because it enables non-instrumental sensing of various chemical species such as ions or neutral molecules, e.g. for monitoring of ions of biological or/and environmental importance. The receptor molecule besides binding site should be equipped with additional signaling unit, a functional group joined via linker or chromophoric/fluorophoric moiety forming an integral part of the molecule. Schematically, the idea of chromo- and fluorogenic molecular receptors is shown in Scheme 6. The mechanism of sensing depends on the nature of both the host and the guest. The binding mode, selectivity and sensitivity can be also influenced or controlled by the effect of the solvent and/or receptor immobilization on solid surfaces of various properties.

Inter alia functionalized macrocyclic compounds bearing azo moiety belong to this relatively popular group of sensing materials.

**Fig. 32:**
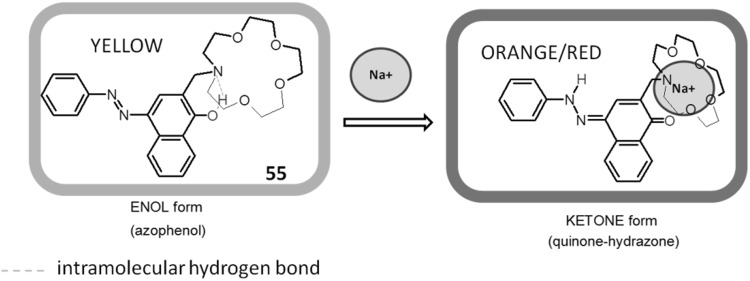
The mechanism of color change of azacrown ether modified with 4-(phenyldiazenyl)naphthalen-1-ol **55** synthesized by Antonov et al. exemplified by sodium complexation [173]

In the case of *para*- and *ortho*- hydroxyderivatives of azocompounds the color signaling mechanism may be associated with the change in the tautomeric equilibrium upon complexation. This is well illustrated by tautomeric switch based on functionalized azacrown ether **55** (Fig. 32) synthesized and investigated by Antonov and co-workers [173]. Uncomplexed ligand in acetonitrile exists in azophenol form stabilized by intramolecular hydrogen bond between phenolic OH group and nitrogen atom of crown ether residue. In the presence of alkali and alkaline earth metal cations—the color of the solution turns from yellow to orange–red, what is a result of bathochromic and hyperchromic effects in UV–Vis spectra. The complex formation is connected with the shift of the tautomeric equilibrium towards ketone (quinone-hydrazone) form. Metal cations are complexed by ether oxygen donor atoms and by carbonyl oxygen atom of ketone form.

**Fig. 33:**
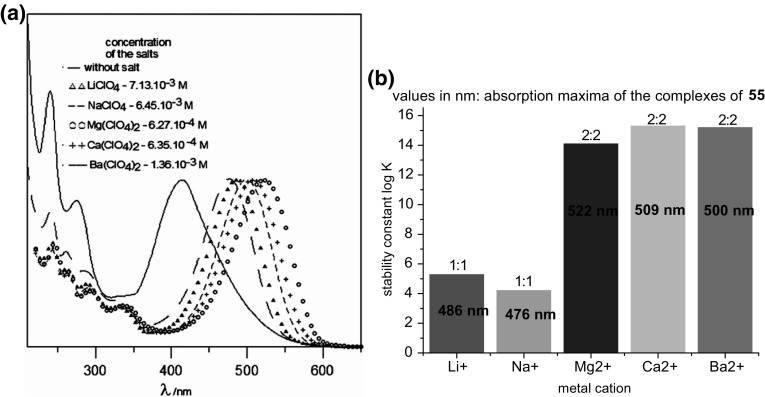
**a** Normalized absorption spectra of azacrown **55** (─) in CH_3_CN and its final complexes with alkali and alkaline earth metal ions. Reprinted from [173]. Copyright 2010 with permission from Elsevier. **b** the values of the stability constants of azacrown with metal cations and the position of the absorption maxima for the respective complexes

Lithium and sodium cations form complexes of 1:1 stoichiometry with azacrown **55** (Fig. 33). For magnesium and calcium initially 1:1 complex is formed. Under an excess of a metal salt 2:2 complex dominates. Direct 2:2 complex formation was found for barium perchlorate. Absorption spectra of azacrown registered in the presence of metal perchlorates are shown in Fig. 33a. In Fig. 33b the values of the stability constants of 1:1 and 2:2 metal complexes with discussed azacrown **55** are presented.

**Fig. 34:**
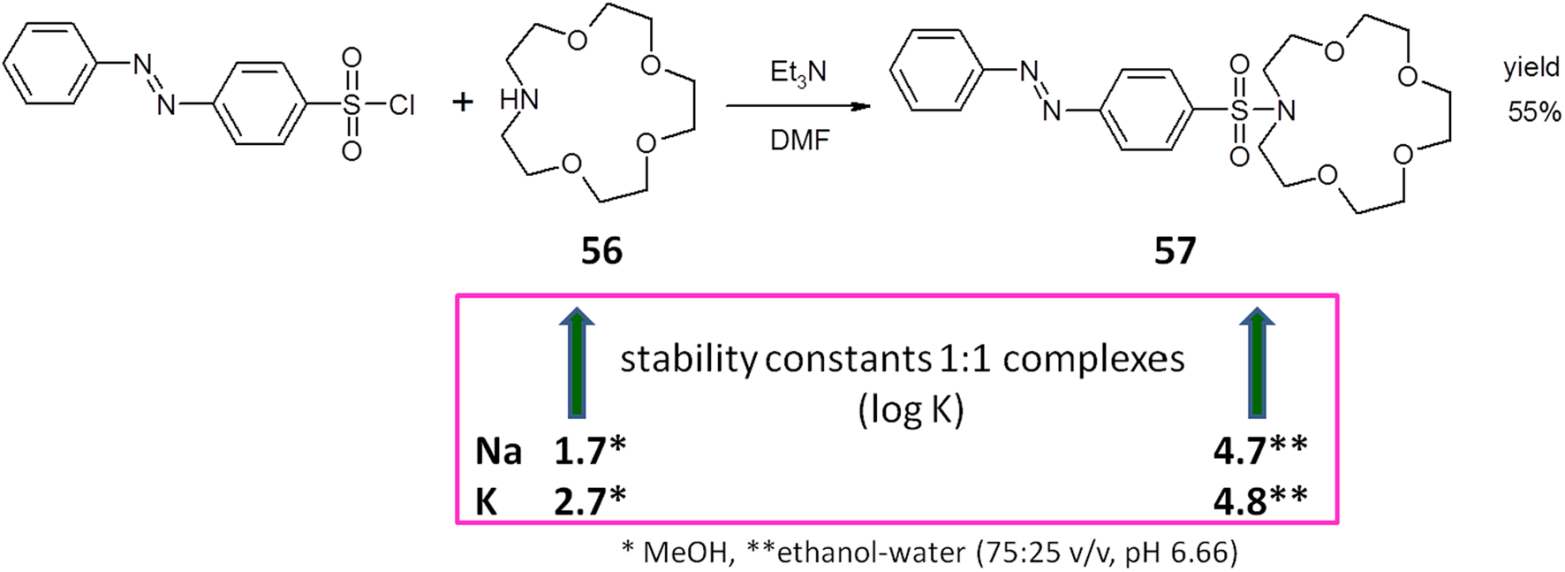
Lariat ether: *N*-[4-(phenyldiazo)benzenesulfonyl]-aza-15-crown-5 **57**—synthesis and comparison of sodium and potassium complexes stability constant values with parent aza-15-crown-5 **56** [174–178]

Aza-15-crown-5 **56** (Fig. 34) skeleton is a hopeful building block for colorimetric sensors. Lincoln and Sumby [174] used this macrocyle to synthesize *N*-[4-(phenyldiazo)benzenesulfonyl]-aza-15-crown-5 **57** (Fig. 34). This chromogenic compound was obtained in 55% yield by treating commercially available 4-phenyldiazobenzenesulfonyl chloride with aza-15-crown-5 in DMF in the presence of triethylamine. The synthesized lariat ether was studied as metal cation reagent in ethanol–water (75:25 v/v, pH 6.66) mixture. The stability constant values of 1:1 complexes of sodium and potassium cations with **57** are higher than for the parent aza-15-crown-5 **56** (Fig. 34) and its derivatives [175–178]. The solved X-ray structure of [Na(**57**)(H_2_O)]_2_(ClO_4_)_2_ complex showed that it is a dimer with the sulfonamide oxygen atom engaged in cation complexation. This indicates the cooperation of sulfonamide side arm and crown ether moiety in ion binding and explains the higher values of the stability constant compared with data for unsubstituted aza-15-crown-5.

**Scheme 7:**
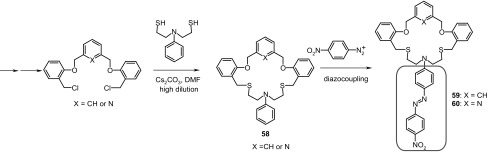
Synthetic route for preparation chromogenic macrocyles **59** and **60** [179]

The selectivity of metal cation binding can be controlled by using macrocycles with softer, sulfur donor atoms. Lee and Lee [179] synthesized, under high dilution conditions, macrocyclic derivatives incorporating aromatic moiety, i.e. benzene **59** or pyridine **60** (Scheme 7) within the macroring. Chromogenic character of macrocycles was achieved by extending the structure by diazocoupling of the obtained in the first step *N*-phenylated macrocyles **58** with *p*-diazonium salt.

**Fig. 35:**
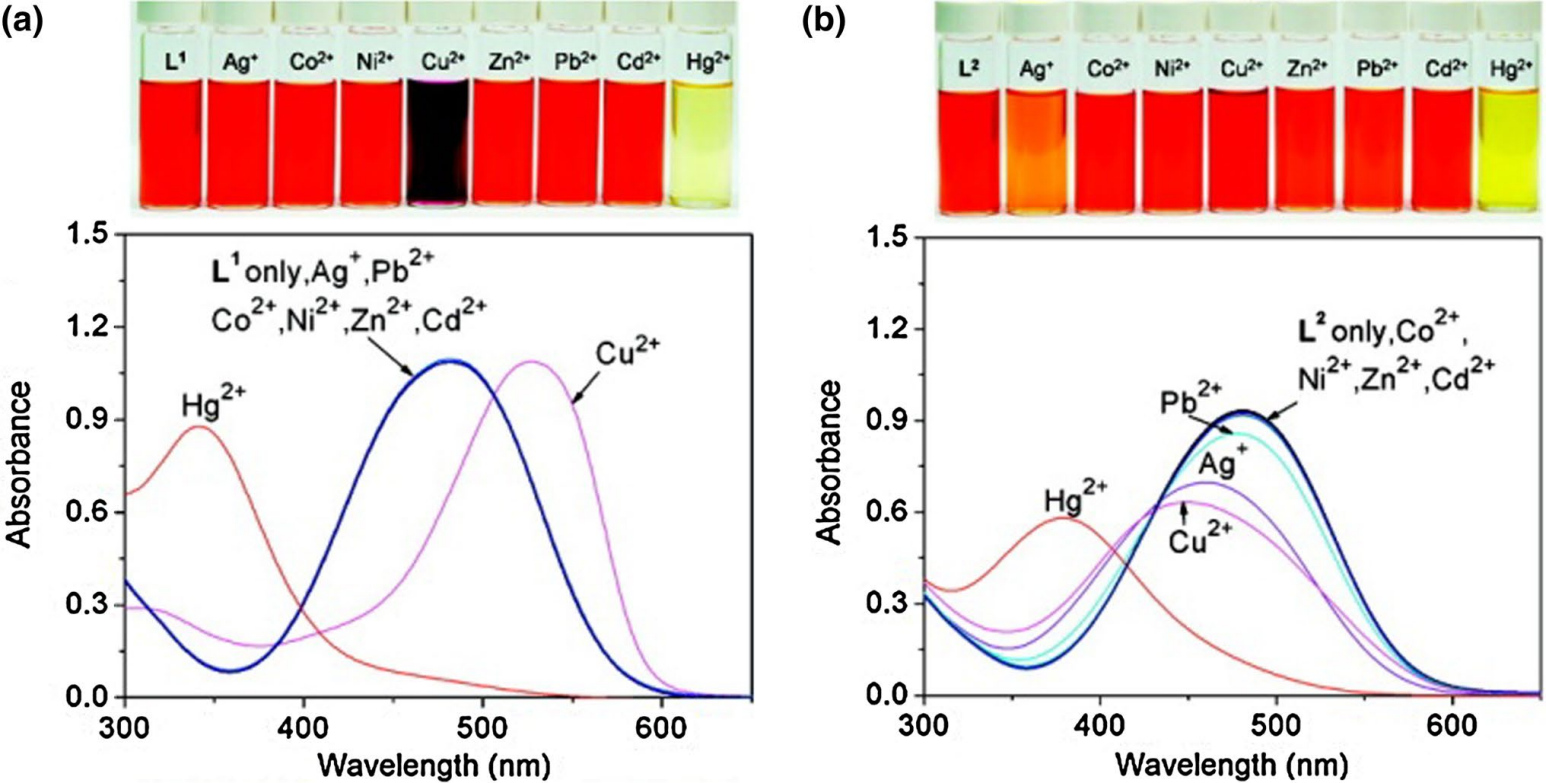
UV–Vis spectra of **a 59** and **b 60—**(40 μM) in the presence of metal perchlorates (5.0 equiv) in acetonitrile. Numbers of compounds in reproduced material correspond to following numbers of compounds in this work: L^1^ = **59**; L^2^ = **60**. Reprinted with permission from [179]. Copyright 2009 American Chemical Society

Both compounds **59** and **60** selectively bind mercury(II) in acetonitrile forming 1:1 complexes. Complexation of Hg^2+^ causes hipsochromic shift of absorption bands from 480 to 339 and 378 nm for **59** and **60**, respectively. Among other investigated metals only copper(II) cations cause bathochromic shift of absorption band of **59**, whereas spectral behavior of **60** remains intact. Color and spectral changes of **59** and **60** in acetonitrile solutions in the presence of metal salts are shown in Fig. 35. The crystal structure of **60** complex with mercury(II) ion showed metal cation located inside the macrocycle cavity. The difference in selectivity towards mercury ions versus other metal cations was explained by the engagement of the pyridine nitrogen atom in complex formation in case of **60**.

**Fig. 36:**
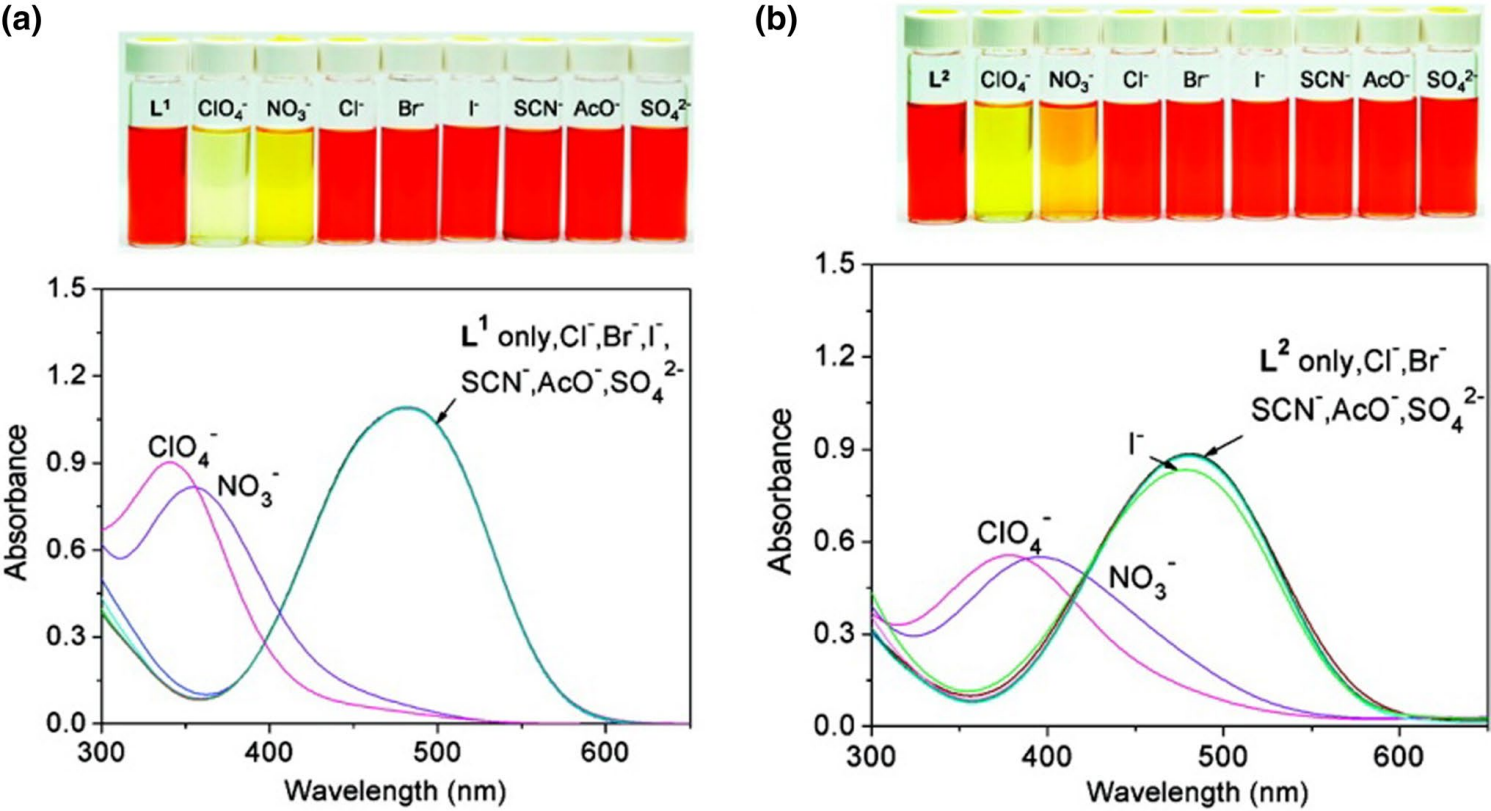
UV–Vis spectra of **a 59** and **b 60 -** (40 *μM*) in the presence of Hg^2+^ (5.0 equiv) upon addition of anion salts in acetonitrile. Numbers of compounds in reproduced material correspond to following numbers of compounds in this work: L^1^ = **59**; L^2^ = **60**. Reprinted with permission from [179]. Copyright 2009 American Chemical Society

Spectral and color changes in the presence of Hg^2+^ were found to be anion dependent (Fig. 36). Addition of perchlorates or nitrates to the acetonitrile solution of mercury(II) complexes of **59** and **60** causes spectral and color changes, which can be attributed to the ability of mercury to coordinate these anions. The obtained results indicate that proposed macrocycles can be used not only as mercury, but also as anion sensing molecules.

**Scheme 8:**
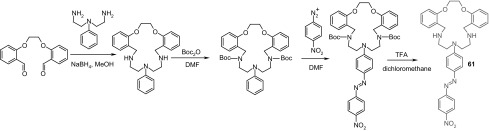
Synthesis of chromogenic macrocyle **61** [180]

Spectral and color changes caused by complexation of heavy metal cations were also found for macrocyle **61** bearing as chromogenic substituent *p*-nitroazobenzene [180] that was obtained in multistep reaction shown in Scheme 8.

**Fig. 37:**
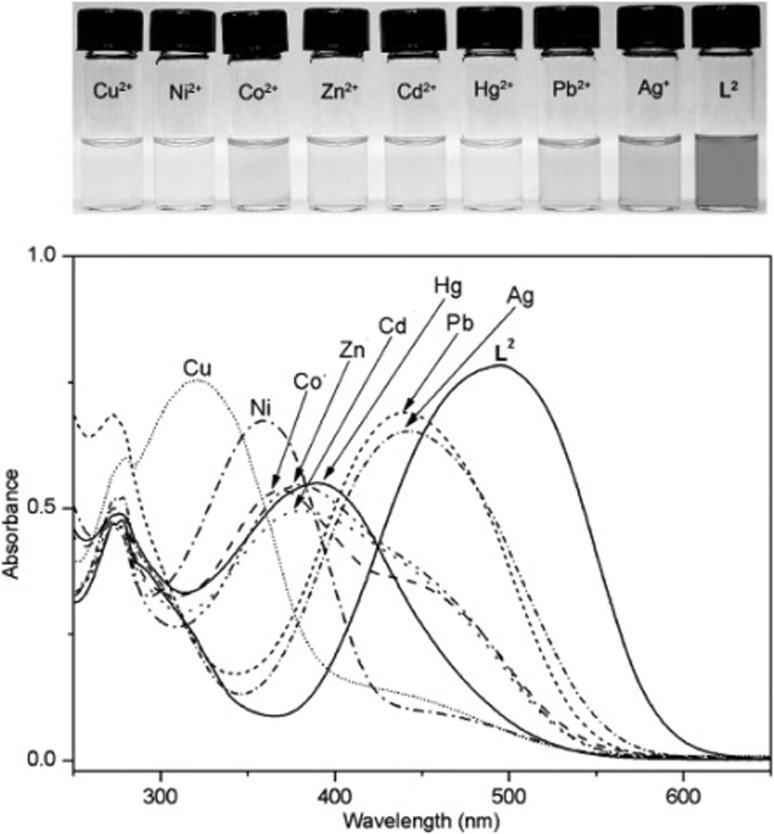
Changes in the UV–Vis spectrum of **61** on addition of metal nitrates in acetonitrile (ligand concentration, 5.0 × 10^−5^ M; and added metal ion, 3.0 equiv). Number of compound in reproduced material corresponds to following number of compound in this work: L^2^ = **61**. Reprinted with permission from [180]. Copyright 2009 American Chemical Society

Red acetonitrile solution of **61** changes color to yellow upon addition of metal salt, which is a result of metal cation induced hypsochromic shift of absorption band. The largest spectral and color changes among investigated metal cations causes copper(II) (Δ*λ*_max_ = 174 nm). The spectral and color changes of **61** in the presence of metal nitrates are shown in Fig. 37.

Compound **61** forms two types of solid state complexes, which differ in color: [Cu(**61**)NO_3_]NO_3_·CH_2_Cl_2_, a pale-yellow and dark red [{Cu(**61**)}2(*μ*OH)_2_](ClO_4_)_2_·2CH_2_Cl_2_·2H_2_O. The effect of counter ion on spectral changes upon copper(II) complexation was investigated using chloride, nitrate, perchlorate, acetate, and sulfate salts. A blue shift was observed and the influence of anion can be set in the following order: NO_3_^−^, ClO_4_^−^ > Cl^−^, AcO^−^ > SO_4_^2−^, which is in accordance with the Hofmeister series of relative anion lipophilicities.

**Scheme 9:**
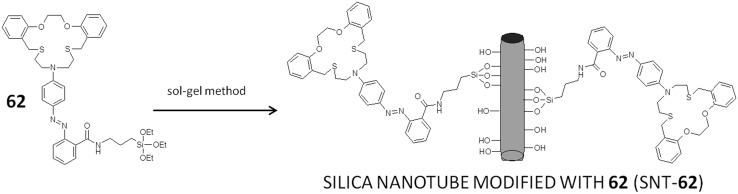
Schematically: modification of silica nanotubes with chromogenic macrocylic derivative **62** (SNT-**62**) [181]

Colored systems can be also used for preparation of sensing materials by immobilization of the respective receptor(s) on a chosen solid surface. For example, macrocycle **62** (Scheme 9) bearing azo unit, was immobilized on a silica nanotubes (SNT) using sol–gel method [181]. The described system (SNT-**62**) was presented as a heterogenous “naked-eye” and spectrophotometric metal cation sensor.

**Fig. 38:**
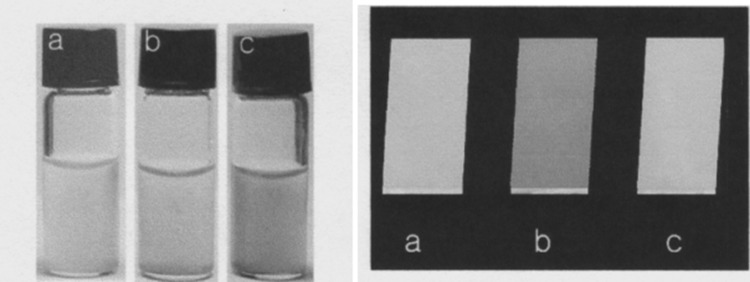
Left: pictures of the suspensions: **a** SNT-**62**, **b** SNT-**62** + 0.01 mM Hg(NO_3_)_2_, **c** STN-**62** + 1.0 mM Hg(NO_3_)_2_. Right color changes of glass plates coated with SNT-**62: a** before immersion and after immersion in **b** Hg^2+^ (0.01 mM) and **c** Cu^2+^ (0.01 mM) solution in water. Reprinted from [181]. Copyright 2007 with permission from John Wiley and Sons

Inorganic–organic nanomaterial (SNT-**62**) shows in water selective response by color change from yellow to violet towards Hg^2+^ among all other investigated metal cations: Ag^+^, Co^2+^, Cd^2+^, Pb^2+^, Zn^2+^, Fe^3+^, Cu^2+^. The color of suspensions also changes in the presence of nitrate and perchlorate anions from yellow to pink and violet, respectively. The addition of Cl^−^, Br^−^, I^−^, SCN^−^, or SO_4_^2−^ salts does not cause color change. It was also shown that modified silica nanotubes can act not only as colorimetric sensor for mercury(II) cation, but also for preparation of stationary phases for ion chromatography. The use of suspensions can be sometimes troublesome, thus a portable chemosensor kit was prepared by modification of the glass surface with SNT-**62**. The material also in this form exhibits selective response towards Hg^2+^ with color change from yellow to violet upon dipping in solution of mercury(II) salt. Color changes of the water suspensions of SNT-**62** upon addition of mercury(II) nitrate at different concentrations are shown in Fig. 38 (left). The color change of the glass sensor modified with SNT-**62** upon immersion into mercury(II) and for comparison copper(II) aqueous solutions is shown in Fig. 38 (right).

**Fig. 39:**
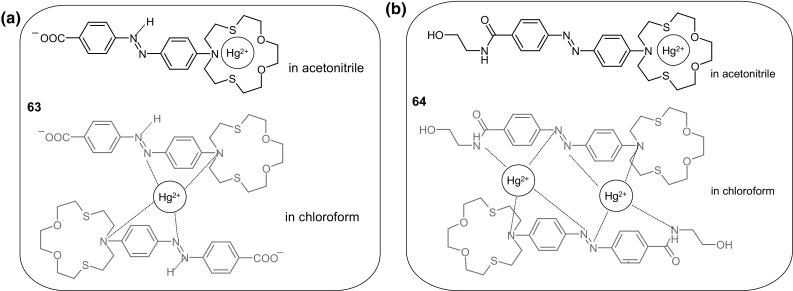
Proposed mechanism of mercury(II) complexation by macrocycles **63** and **64** depending on the solvent type [182]

Environmentally hazardous mercury(II) sensing based on dithiaazadioxo crown ether system with peripheral azo unit was described by Ha and co-workers [182]. Compounds **63** and **64** (Fig. 39) were investigated as Hg^2+^ receptors in solvents of diverse polarity (acetonitrile, its mixture with water and in chloroform). It was found that host–guest interaction strongly depends on the solvent nature. According to ^1^H NMR and spectrophotometric measurments it was stated that both ligands in acetonitrile form 1:1 complexes, if Hg^2+^ is coordinated inside the macrocyclic cavity (Fig. 39). As a consequence of molecular recognition solutions of both ligands undergo discoloration in the presence of Hg^2+^ ions. In less polar chloroform, different mechanism of ligand-ion interaction was proposed. Two molecules of **63** probably bind one mercury(II) cation forming sandwich complex. This is manifested by color change from yellow to pink. In the case of macrocycle **64** in chloroform complexes of 2:2 stoichiometry are formed.

**Fig. 40:**
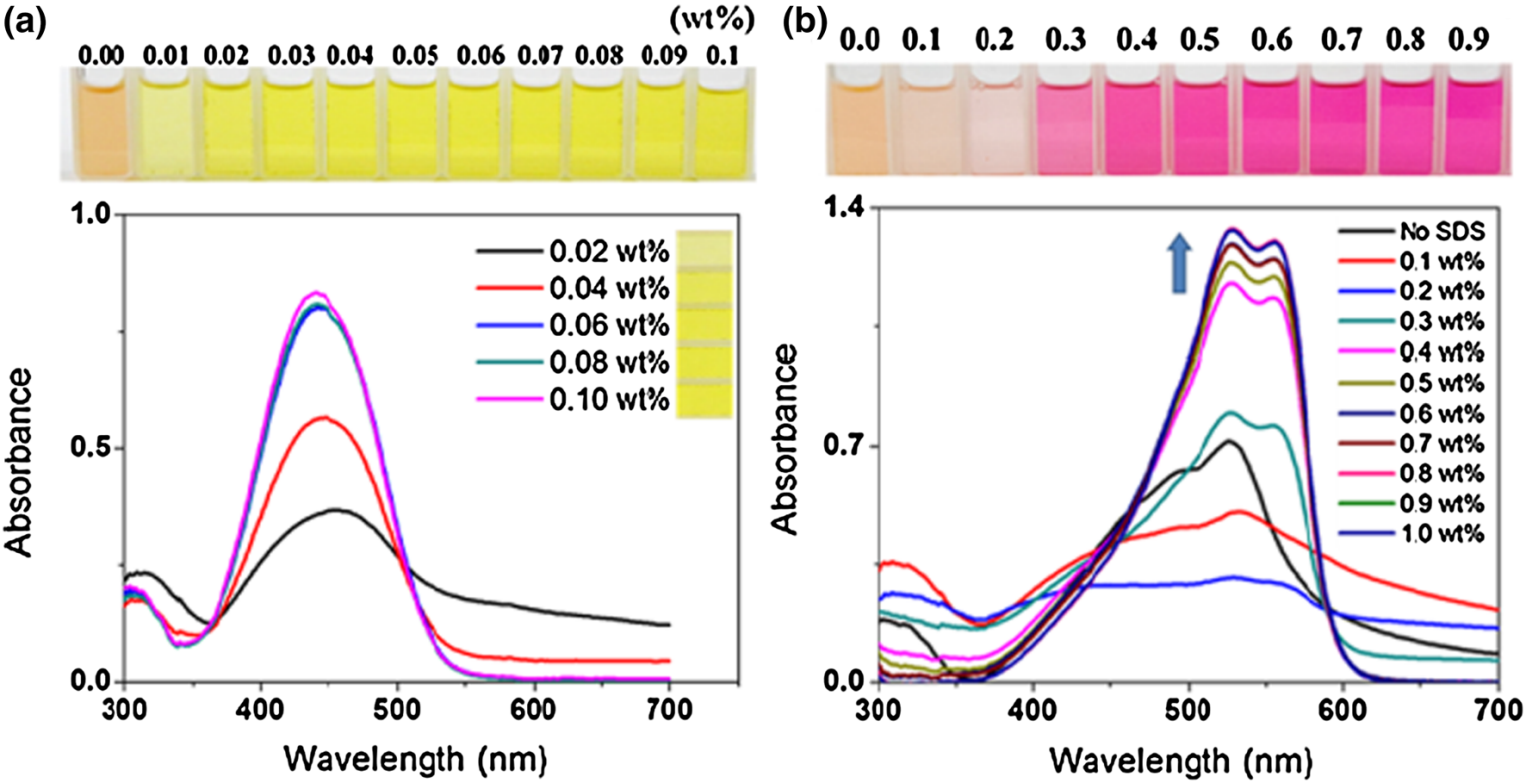
Color and spectral changes of **63** in various concentrations of  **a** CTAB and **b** SDS. Reprinted from [183] Copyright 2015 with permission from John Wiley and Sons. (Color figure online)

Ha and Jeon continued the work on selective mercury(II) sensing using compound **63** (Fig. 39) [183]. The colored macrocycle was applied for recognition of Hg^2+^ ions in aqueous solution. The effect of two surfactants cetyltrimethylammonium bromide (CTAB) and sodium dodecylsulfate (SDS) on spectral and color behavior of **63** was investigated. In the presence of CTAB the solution of **63** is yellow, while pink color is observed in the presence of SDS (Fig. 40). In the presence of Hg^2+^ the pink solution of **63**-SDS system becomes colorless enabling naked-eye ion recognition with detection limit 1.6 μM. The **63**-SDS based system was also used for preparation of the mercury sensitive cellulose test strips.

## Functionalized azobenzocrowns (azo moiety as a part of the macrocycle)

This chapter highlights the preparation and properties of azobenzocrowns, of different size of the macrocycle, equipped with additional functional groups in benzene rings: hydroxyl, amino, and dimethylamino, as well as *pull*–*push* type azobenzocrowns with nitro and dimethylamino groups.

**Scheme 10:**
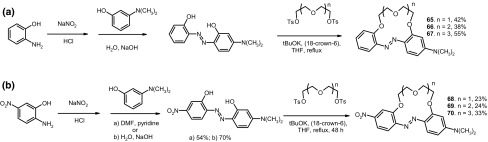
Synthesis of functionalized azobenzocrowns **65–70** [159]

13-, 16- and 19-membered crowns bearing electron donating (dimethylamino) **65**–**67** or two different electron donating/accepting groups (dimethylamino and nitro) **68**–**70** in the azobenzene fragment (Scheme 10) were obtained with the yields up to 55% [159].

**Fig. 41:**
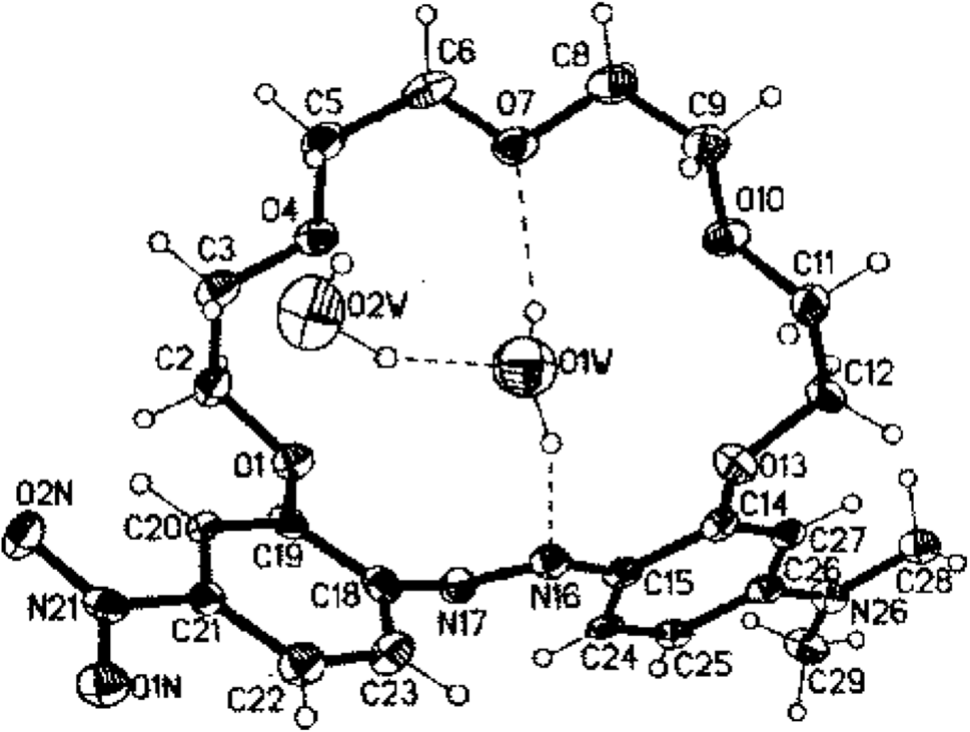
Molecular structure of **70**·2H_2_O with atom labeling scheme; ellipsoids are drawn at 50% probability level. Reprinted from [159]. Copyright 2005 with permission from Elsevier

Compounds **65–70** exist only in *E* form, both in the solid state and in solution. In Fig. 41 X-ray structure of **70**·2H_2_O is presented, showing the *E* geometry of the azo unit with aromatic moieties in the *trans*-positions and proved the existence of a molecular diaqua-complex [159].

**Fig. 42:**
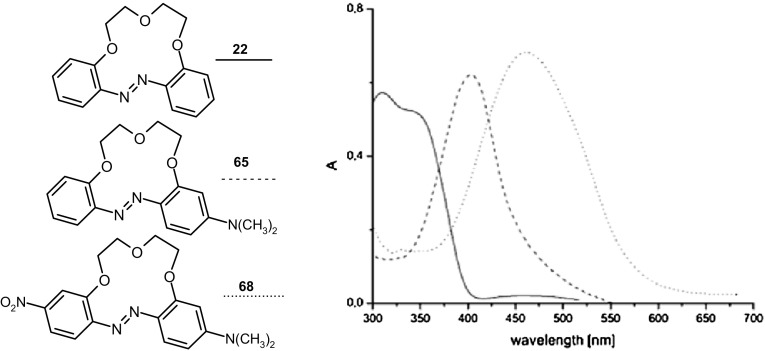
Comparison of absorption spectra of azobenzocrowns: parent—**22** (solid line), and functionalized with: dimethylamino—**65** (dashed line) and dimethylamino- and nitro—**68** (dotted line); (c = 7.0 × 10^−5^ M) in acetonitrile. Spectra reproduced from [159]. Copyright 2005 with permission from Elsevier

The absorption spectra of **65–70**, opposite to the parent azobenzocrowns **22**–**24** (Fig. 16a) have sharp and well pronounced maxima (Fig. 42).

**Fig. 43:**
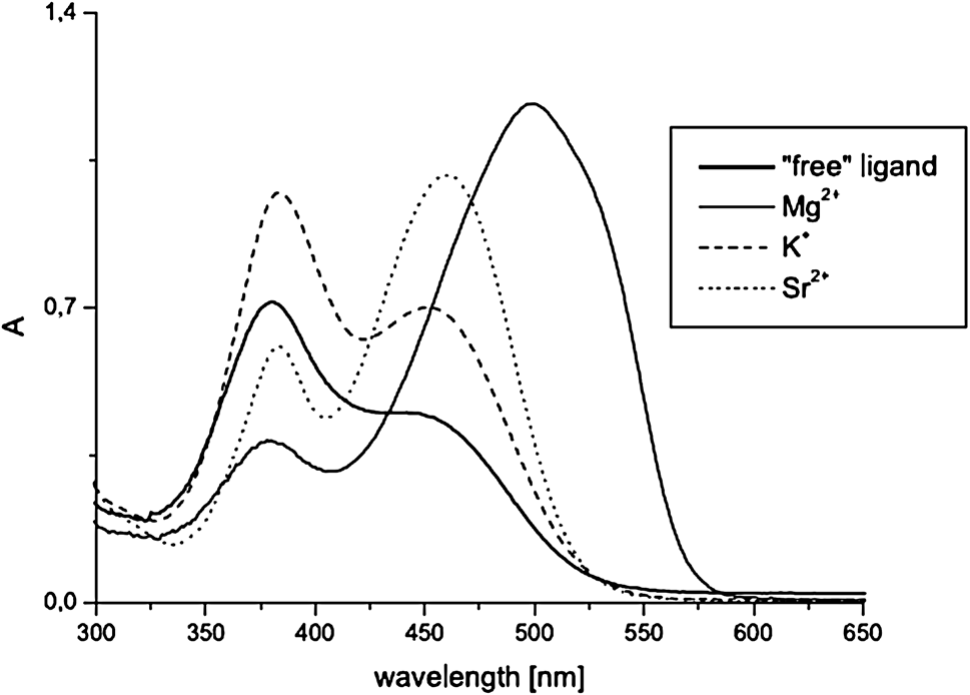
The comparison of absorption spectra of **67** (solid line, c = 5.7 × 10^−5^ M) and limiting spectra in the presence of: potassium (dashed line, c = 2.6 × 10^−3^ M), strontium (dotted line, c = 4.4 × 10^−5^ M) and magnesium (solid line with maximum at ~ 520 nm, c = 1.9 × 10^−3^ M) perchlorates in acetonitrile. Reprinted from [159]. Copyright 2005 with permission from Elsevier

The UV–Vis studies of alkali and alkaline metal cation complexation by compounds **65–70** showed magnesium selectivity of 19-membered azobenzocrown **67** in acetonitrile. Only in this case the complexation is characterized by significant spectral shift (Fig. 43) and by distinctive color change from orange to pink.

Another set of synthesized and investigated functionalized azobenzocrowns consists of derivatives with a hydroxyl substituent. Azobenzocrowns with hydroxyl group located in one of the benzene rings, in the *para* position to the azo group, have been synthesized prior to 2002 [158] and are also a part of current works carried in Luboch’s group.

**Scheme 11:**
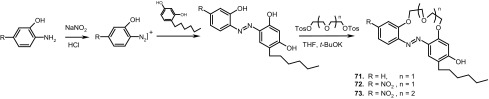
The synthesis of azobenzocrown ethers with peripheral hydroxyl group **71**–**73—**derivatives of 4-hexylresorcinol [160]

A simple method for the synthesis of 13- and 16-membered azobenzocrown ethers, derivatives 4-hexylresorcinol **71**–**73** with two peripheral groups, i.e. nitro and hydroxyl groups at two opposite sides of the conjugated chromophoric system has been described by Luboch et al. (Scheme 11) [160].

**Fig. 44:**
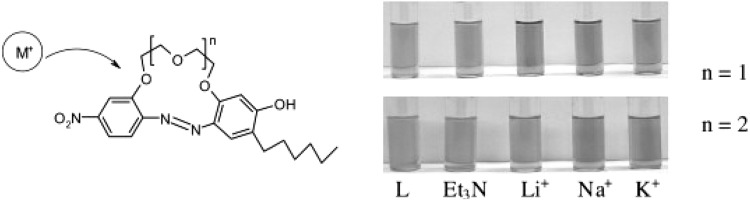
Color changes of azobenzocrowns solutions with peripheral hydroxyl group **72** (n = 1) and **73** (n = 2) in the presence of metal perchlorates in acetonitrile. Reprinted from [160]. Copyright 2009 with permission from Elsevier

Typical for 13-membered azobenzocrowns, including compounds **71** and **72** is selective binding of lithium cations. The most significant, among all investigated so far compounds of this type, is the spectral shift of 95 nm and color change from yellow to pink found for **72** (Fig. 44) in basic acetonitrile (Et_3_N) solution. Crown **73** is more lithium sensitive, but less selective, versus sodium and potassium (Fig. 44) giving the color change. The chromoionophoric behavior of the compounds potentially allows their application, under selected conditions, for construction of optical sensors.

**Scheme 12:**
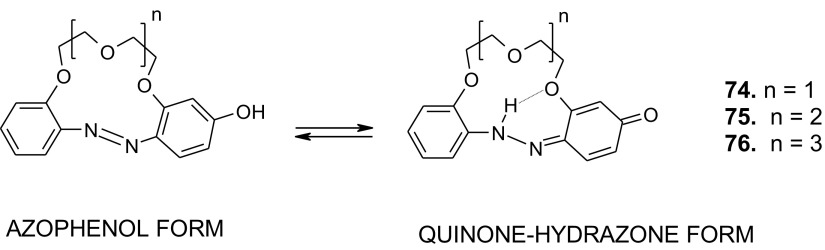
Tautomeric equilibrium for hydroxyazobenzocrown ethers **74–76** showing hydrogen bond inside the cavity of quinone-hydrazone form [158]

As shown in Scheme 12 the hydroxyazobenzocrowns undergo tautomeric equilibrium to quinone-hydrazones. The tautomeric equilibrium of hydroxyazobenzocrowns is affected by the size of the macrocycle. The larger the cavity size the lower the tendency to occur in the quinone-hydrazone form. This can be explained by weaker hydrogen bonds in macrocycles of larger cavity. 13-membered macrocyclic *p*-hydroxyazobenzene derivative—compound **74** (Scheme 11), in the solid state and in solvents of different polarity (chloroform, acetonitrile, acetone or methanol) exists in the quinone-hydrazone form. The azophenol form was observed (~ 30%) in DMSO.

16-Membered crown **75** in chloroform and in acetonitrile exists, like **74**, in the quinone-hydrazone form, but in DMSO only the azophenol form was found. Compound **76** of 19-membered ring entirely exists in the azophenol form in DMSO and chloroform. In acetonitrile no less than 75% of this form was detected [159], but in acetone both forms exist in comparable amounts.

**Scheme 13:**
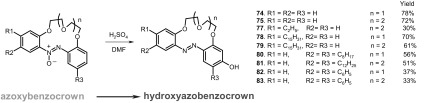
Rearrangement of azoxybenzocrowns in the presence of concentrated sulfuric acid and DMF [165]

*p*-Hydroxyazobenzocrown ethers can be obtained from *O*-protected podands by reduction [158] or directly from dihydroxyazocompounds as shown for stericaly hindered crowns **71–73** [160]. The reaction analogous to the Wallach rearrangement was proposed as a method for preparation of *p*-hydroxyazobenzocrowns using azoxybenzocrowns as substrates [161]. However, the reaction carried out in the mixture of concentrated sulfuric acid and ethanol suffers from the formation of side products and large amounts of used reagents [162]. Exhaustive synthetic research on the applicability of Wallach rearrangement allowed to conclude that the decrease of the side-products formation, lower amounts of reagents and finally, the most importantly, significant yield increase is obtained by carrying out the Wallach rearrangement in a mixture of concentrated sulfuric acid and dimethylformamide [165]. Under elaborated reaction and isolation conditions a series of hydroxyazobenzocrowns **74, 75** and **77**–**83** were successfully obtained (Scheme 13).

**Scheme 14:**
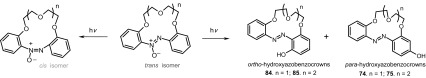
Azoxybenzocrowns: *trans*–*cis* photoisomerisation and photochemical rearrangement leading to *ortho*- (**84, 85**) and *para*-hydroxyazobenzocrowns (**74, 75**) [165]

In contrast to the Wallach rearrangement conducted under strongly acidic conditions where mostly *p*-hydroxyazo compounds are formed, the photochemical rearrangement leads also to *ortho*-hydroxyazo compounds **84, 85** (Scheme 14) [162]. Under fixed conditions the ratio of *para* to *ortho* hydroxyazobenzocrown isomers was dependent on the solvent. In toluene *o*-substituted compounds were dominating, *p*-substituted crowns were the main product in ethanol, whereas in DMF a mixture of comparable amounts of both isomers were obtained.

*o*-Hydroxyazobenzocrowns opposite to *p*-substituted analogs, exist mainly in azophenol form.

**Fig. 45:**
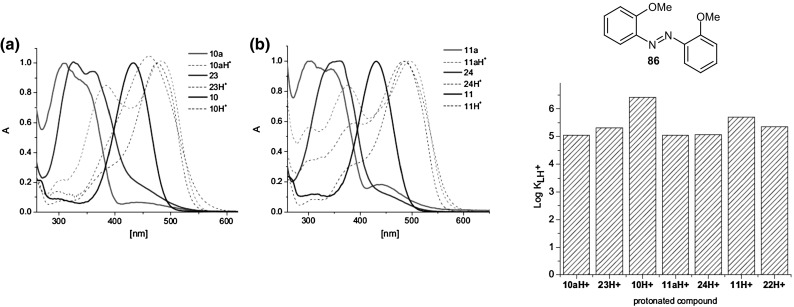
Left: comparison of normalized UV–Vis spectra of (a) 13-membered (b) 16-membered azobenzocrowns (solid) and their protonated forms (dashed lines) Right: proton binding constants for **22, 84, 74** and **23, 85, 75** azobenzocrowns and for acyclic analog **86** in acetonitrile. Number of compounds in reproduced material correspond to following numbers of compounds in this work: 10 = **74**; 10a = **22**; 23 = **84**; 11 = **75**; 11a = **23**; 24 = **85**; 22 = **86**. Reprinted from [165]. Copyright 2013 with permission from Elsevier. (Color figure online)

The spectral properties of *o*-hydroxyazobenzocrowns **84** and **85** were compared with **74** and **75**, and unsubstituted crowns **22** and **23** (Fig. 16a) [165]. Their normalized UV–Vis spectra (acetonitrile) (solid lines) and the corresponding protonated forms (dashed lines) are shown in Fig. 45a, b. Protonation constants in acetonitrile solutions are compared in Fig. 45 (right). The protonation constants can be ordered: *p*-hydroxyazobenzocrowns > *o*-hydroxyazobenzocrowns > unsubstituted azobenzocrowns ~ acyclic analog of azobenzocrowns **86**.

**Scheme 15:**
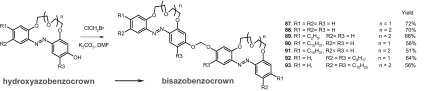
Synthesis of bisazobenzocrowns **87**–**93** with dioxymethylene spacer [165]

*p*-Hydroxyazobenzocrowns were used as substrates in the synthesis of bisazobenzocrowns (Scheme 15) of different lipophilicity, where two macrocyclic residues are joined via dioxymethylene group. Biscrowns (**87–93**) were obtained in yields up to 72%.

Bisazobenzocrowns were used as ionophores in classic and miniature (screen-printed) ion-selective electrodes. A selectivity coefficient logK_Na,K_ = − 2.5 (SSM, 10^−1^ M) for electrode with crown **87** as ionophore was one of the best result obtained for the whole group of the electrodes based on the 13-membered azobenzocrowns. Within the investigated 16-membered bisazocrowns the best potassium over sodium selectivity coefficient for potassium electrodes was logK_K,Na_ = − 3.5 (SSM, 10^−1^ M) found for compound **88**.

**Scheme 16:**
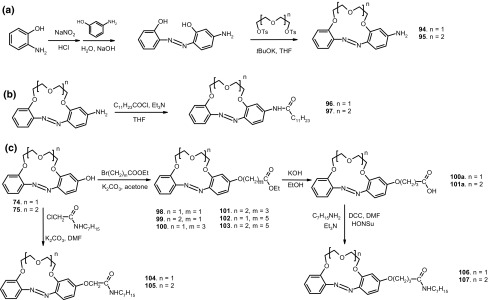
Synthesis of **a** amino (**94, 95**), **b** amide (**96, 97**), **c** ether–ester (**98–103**) or ether–amide (**104**–**107**) derivatives of azobenzocrowns [184]

13- and 16-Membered azobenzocrowns (Scheme 16) with aromatic amino (**94, 95**), amide (**96, 97**), ether–ester (**98–103**) or ether–amide (**104**–**107**) residue in *para* position to an azo moiety were synthesized and investigated [184].

**Scheme 17:**
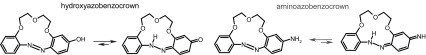
Comparison of tautomerism of 13-membered hydroxy- and aminoazobenzocrowns [184]

The studies of tautomeric equilibrium of aminocrowns **94** and **95** showed that in majority of solvents, similarly to open chain aminoazocompounds [185] they exist in aminoazoform (Scheme 17). It is opposite to discussed above hydroxyazobenzocrowns for which tautomeric equilibrium was found to be more solvent dependent [158, 159, 186].

The protonation of aminoazobenzocrowns shifts the tautomeric equilibrium towards protonated iminohydrazone form.

**Fig. 46:**
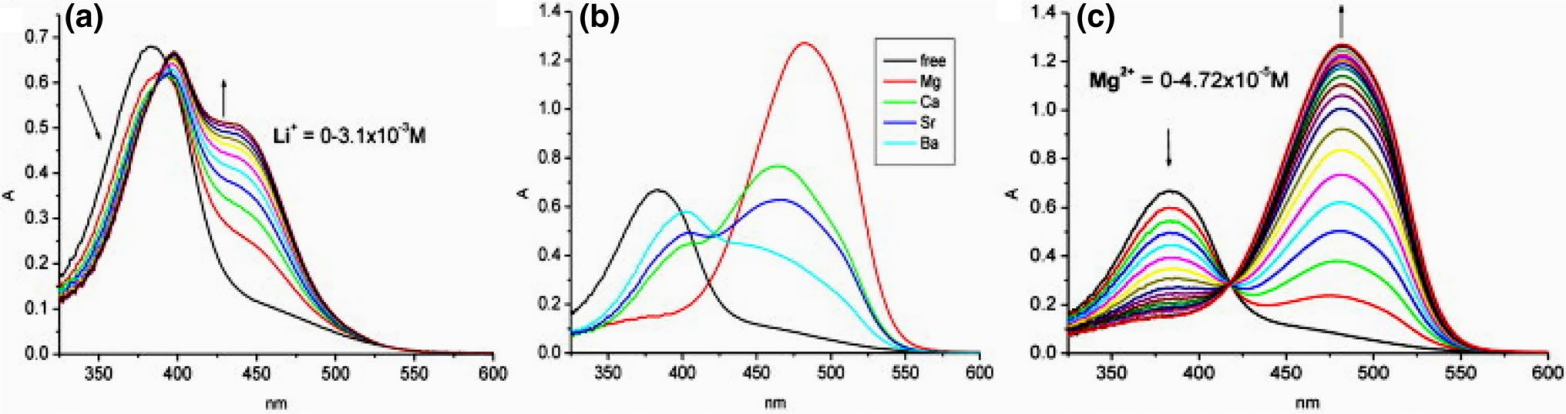
Changes in absorption spectra upon titration of solution of **94** (c = 3.27 × 10^−5^ M) with perchlorates: **a** lithium; **c** magnesium; **b** the limiting spectra obtained during spectrophotometric titration of solution of **94** with alkaline earth metal perchlorates in acetonitrile. Reprinted from [184]. Copyright 2013 with permission from Elsevier. (Color figure online)

13-Membered crown **94**, as expected, in acetonitrile preferentially complexes lithium ions. Stability constant of this 1:1 complex is logK = 4.0. This value is comparable with the value for unsubstituted **22** (logK = 4.1), but it is higher than for the corresponding 13-membered hydroxyazobenzocrown **74**. The stability constant obtained for magnesium complex, logK = 6.43, is the highest value for magnesium complex among all studied so far azobenzocrowns. Changes in the absorption spectra upon spectrophotometric titration of a solution of **94** with lithium and magnesium perchlorates in acetonitrile are illustrated in Fig. 46a, c. Fig. 46b shows limiting spectra for **94** upon titration with alkaline earth metal perchlorates.

16-Membered aminoazobenzocrown **95** forms 1:1 complexes with alkali and alkaline earth metal cations. In all cases, with exception for potassium, the values of the corresponding stability constants are higher than for parent azobenzocrown **23**, which are in turn higher than for complexes of hydroxyazobenzocrown **75**. The introduction of electron-donor amino group into the benzene ring in *para* position to azo moiety enhanced binding properties of azobenzocrowns.

**Fig. 47:**
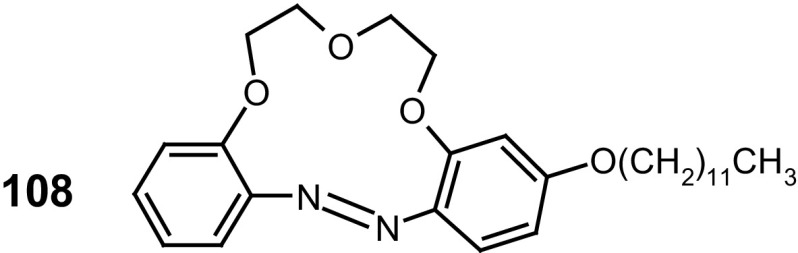
Alkoxy azobenzocrown **108** [184]

Lithium binding was investigated for a series of 13-membered azobenzocrown with oxyalkylcarbonester moiety as side chain **98, 100, 102** (Scheme 16) and was compared with properties of **22** and its alkoxy derivative **108** (Fig. 47).

**Fig. 48:**
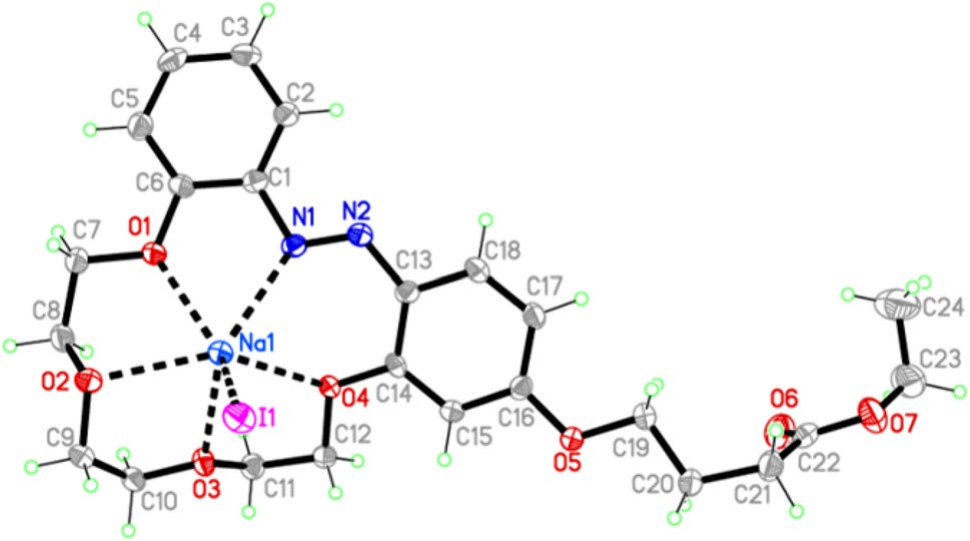
The crystal structure of sodium iodide complex of compound **101**. Reprinted from [184]. Copyright 2013 with permission from Elsevier

The general trend of spectral changes upon lithium complexation for oxyalkylcarbonester derivatives is similar as for **108**. The length of aliphatic acid chain has some effect on the binding strength of the lithium ions, however it cannot be the complexation of the same type as for lariat type crowns. The side chain seems to be too short to participate in complex formation. This is to some extent confirmed by the crystal structure of sodium iodide complex **101** (Fig. 48).

**Fig. 49:**
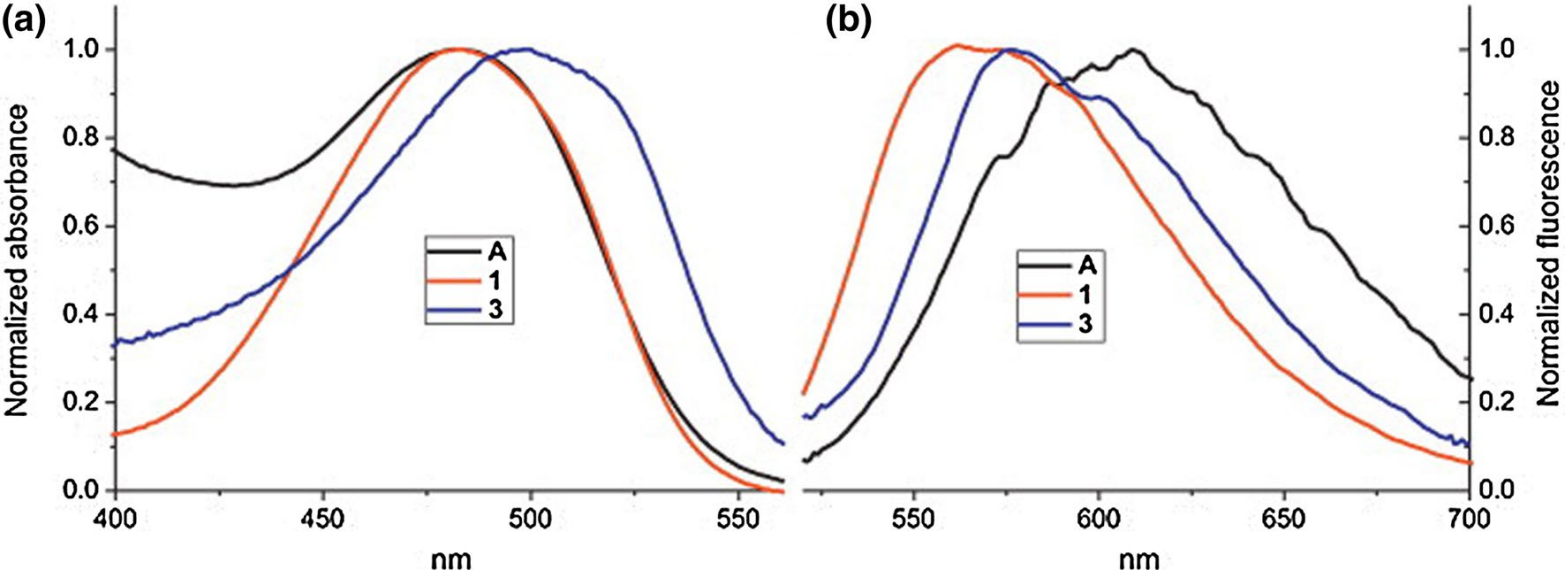
Normalized **a** UV and **b** fluorescence spectra of protonated 13-membered azobenzocrowns **22** (λ_ex_ = 487 nm, λ_em_ = 608 nm), **94** (λ_ex_ = 482 nm, λ_em_ = 568 nm) and **96** (λ_ex_ = 490 nm, λ_em_ = 576 nm) in acetonitrile. Numbers of compounds in reproduced material correspond to following numbers of compounds in this work: A = **22**; 1 = **94**; 3 = **96**. Reprinted from [184]. Copyright 2013 with permission from Elsevier. (Color figure online)

Vast majority of azo compounds, with few exceptions [105, 187–190] show no fluorescence. Protonated azobenzocrowns exhibit orange-red fluorescence. The position of emission band, and the value of the Stoke’s shift is dependent on the presence and nature of the substituent in the *para* position to the azo group [184]. Comparison of normalized absorption and the corresponding emission spectra for protonated forms of 13-membered azobenzocrowns **22, 94** and **96** are shown in the Fig. 49a, b.

**Fig. 50:**
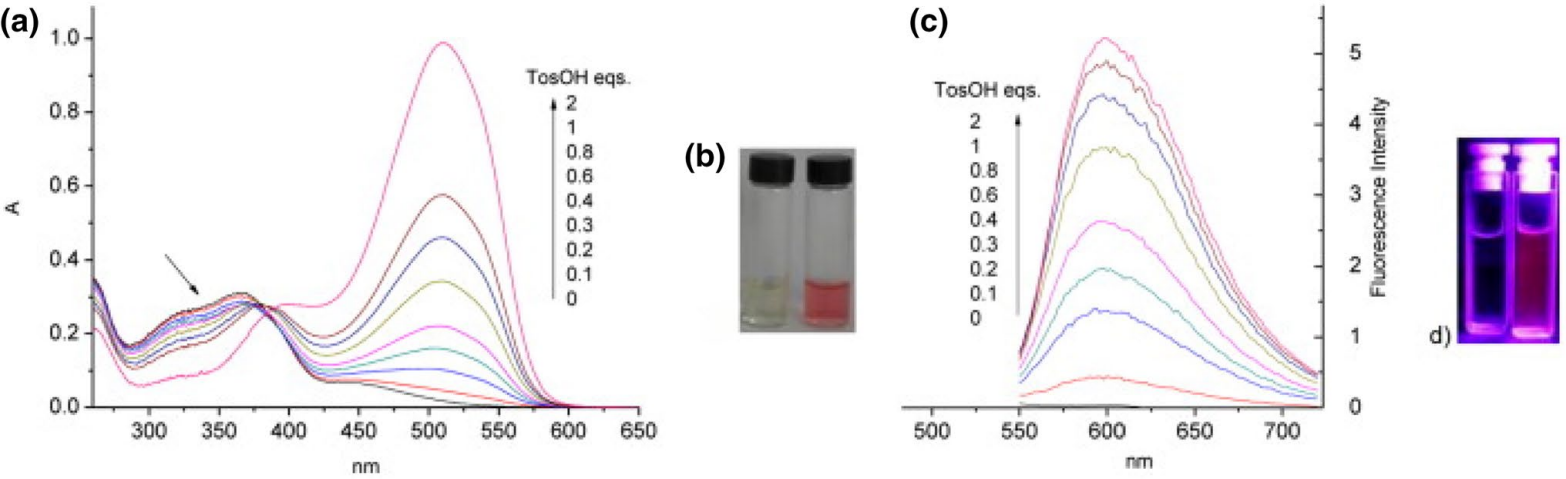
**a** Changes in UV–Vis and **c** in emission spectra of **96** (λ_ex_ = 510 nm, λ_em_ = 598 nm) (3.73 × 10^−5^ M) upon titration its solution with *p*-toluenesulfonic acid solution (TosOH); **b** color change and **d** red fluorescence of **96** in the presence of two-fold excess of TosOH in acetonitrile. Reprinted from [184]. Copyright 2013 with permission from Elsevier. (Color figure online)

Changes in the UV–Vis and emission spectra of compound **96** solution upon titration with solution of *p*-toluenesulfonic acid in acetonitrile are shown in Fig. 50a, b. Photos show a color change and red fluorescence of **96** caused by protonation.

Selected functionalized azobenzocrowns (Scheme 16) were tested as ionophores in ion-selective membrane electrodes. Ether-ester **100**–**103** and ether-amide **104**–**107**, similarly to described earlier alkyl and dialkyl derivatives [136, 137, 158] are good ionophores in membrane electrodes. The mechanism of ion selectivity can be explained by formation of “sandwich” type complexes with the main ions [149, 151].

**Fig. 51:**
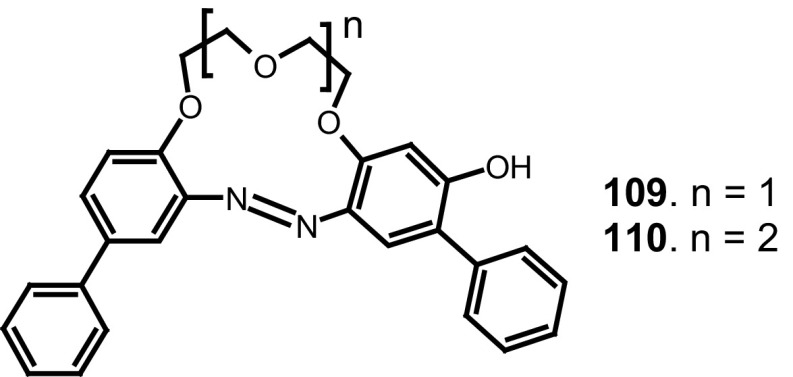
Hydroxyazobenzocrowns **109** and **110** with phenyl substituents in benzene rings [191]

The ^1^H NMR studies of tautomeric equilibrium of hydroxyazobenzocrowns with phenyl substituents in benzene rings (Fig. 51) showed that 13-membered crown **109** in acetonitrile exists in quinone-hydrazone form [191]. 10% of azophenol form was detected in DMSO. For 16-membered crown **110** the presence of quinone-hydrazone form was stated in acetone and chloroform [191] and also in DMSO (50%).

**Fig. 52:**
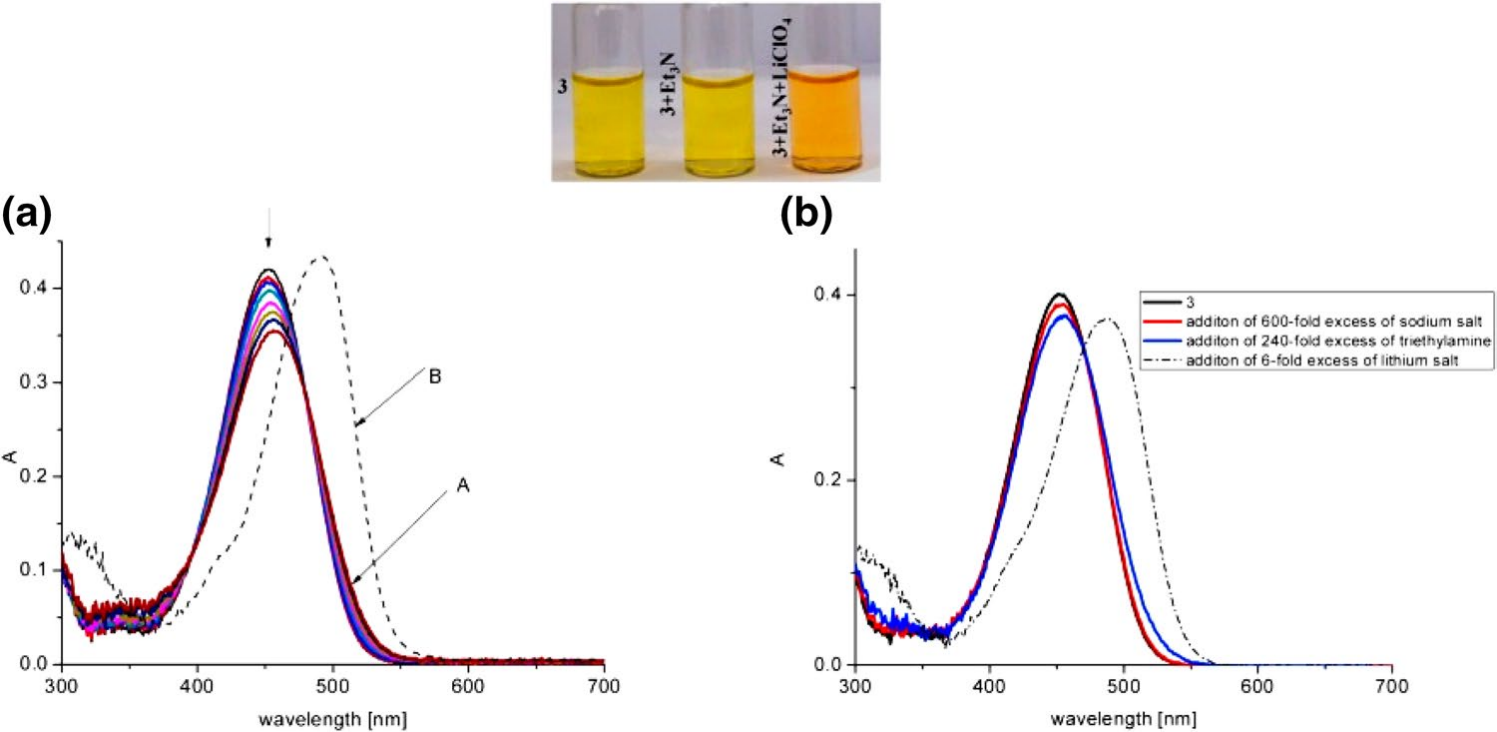
Top: comparison of color acetonitrile solution of 1**09** in the presence of Et_3_N and both Et_3_N and LiClO_4_. Bottom: **a** UV–Vis titration of **109** (7.2 × 10^−5^ M) with LiClO_4_ (0-5.5 × 10^−2^ M) in pure acetonitrile. Dashed lines are spectra registered upon addition to the titrated system solution of Et_3_N (2.8 × 10^−2^ M); **b** UV–Vis spectra showing the competitive binding of lithium by **109** in the presence of sodium salt and triethylamine in acetonitrile. Number of compound in reproduced material correspond to following number of compound in this work: 3 = **109**. Reprinted from [191]. Copyright 2017 with permission from Elsevier

For 13-membered hydroxyazobenzocrown **109** spectral response with spectral shift ~ 40 nm (Fig. 52a) and color change from yellow to orange (Fig. 52, top) caused by the presence of lithium salt was observed only in basic solution (Et_3_N) of acetonitrile. This corresponds to lithium complex formation by ionized hydrazone form of **109**. Lithium response is observed also in the presence of the excess of sodium salt (Fig. 52b).

**Fig. 53:**
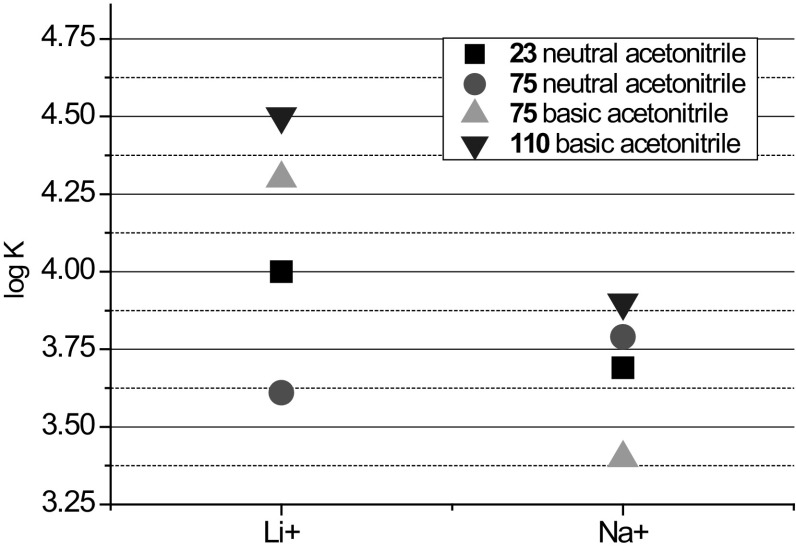
Comparison of stability constant values of complexes of **23, 75** and **110** in neutral and basic acetonitrile calculated from UV–Vis titrations [133, 166, 191]

16-Membered crown without phenyl substituents **75** (Scheme 12) forms complexes both in neutral and basic acetonitrile solution. In pure acetonitrile the presence of alkali and alkaline earth metal cations causes a blue spectral shift corresponding to shift of tautomeric equilibrium and formation of complex in azophenol form. Under the same conditions no spectral changes were observed in the presence of lithium and sodium for **110**. In basic acetonitrile (Et_3_N) for both **75** and **110** the presence of lithium and sodium salts (no changes in the presence of potassium) causes red shift of absorption band. This corresponds to formation of complexes by quinone-hydrazone forms. The comparison of the stability constants (logK), determined by UV–Vis titrations, of lithium and sodium complexes of **75** in neutral and basic acetonitrile and **110** in basic acetonitrile are shown in Fig. 53. Stability constant values of complexes of **23** in neutral acetonitrile were also included for comparison.

Lithium cation is stronger complexed by the ionized quinone-hydrazone form than by azophenol tautomer. For **109** and **110** the presence of phenyl rings appears to be important factor affecting tautomeric equilibrium driven by metal cation complexation and the strength of ion–azobenzocrown interactions.

Spectrofluorimetric titrations of **109** with lithium and sodium perchlorates causes the decrease of fluorescence intensity, especially in the presence of organic base. The value of Stern–Volmer (K_SV_) constant was the highest for lithium in the presence of triethylamine 222 M^−1^. For comparison this value for **74** is 80 M^−1^. For 16-membered diphenyl derivative **110** Stern–Volmer constants in the presence of trietylamine were also higher than for compound without phenyl substituents in benzene rings **75**, both for lithium and sodium. In pure acetonitrile quenching constants caused by metal perchlorates were higher for **75** than for **110**, what can be connected with complex formation by this compound in azophenol form in neutral solvent.

**Fig. 54:**
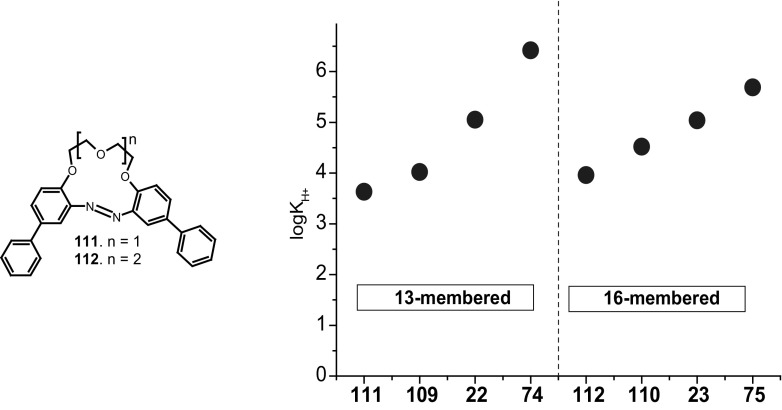
Comparison of proton binding constants for 13- (**111, 109, 22, 74**) and 16-membered (**112, 110, 23, 75**) azobenzocrowns in acetonitrile [191]

Acid-base properties of hydroxyazobenzocrowns were compared with their analogs without hydroxyl substituent by determination of proton binding constants in acetonitrile (Fig. 54). Protonation constants of hydroxyazobenzocrowns **109, 110** and **74, 75** are higher than for compounds without hydroxyl group (**111, 22, 112, 23**) and among hydroxyazobenzocrowns are higher for macrocycles without phenyl substituents in benzene rings **74** and **75**.

**Fig. 55:**
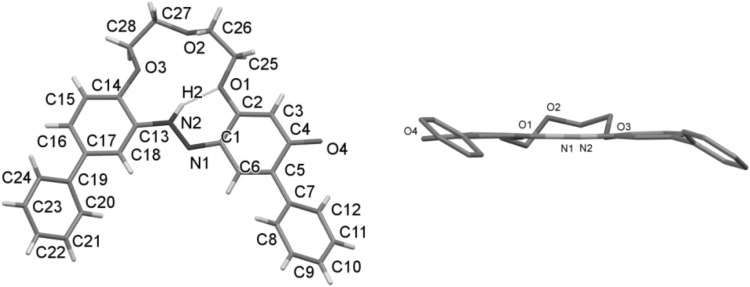
Left: molecular structure and labeling scheme for **109**; Right: view of **109** along the mean plane defined by the azobenzene moiety. Reprinted from [191]. Copyright 2017 with permission from Elsevier

X-ray structure of **109** was solved confirming the existence of this compound in quinone-hydrazone form in solid state (Fig. 55).

**Scheme 18:**
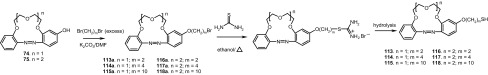
Synthesis of thiol derivatives of azobenzocrown ethers **113**–**118** [164]

*p*-Hydroxyazobenzocrowns can be considered as universal substrates for azobenzocrowns skeleton modifications. 13- and 16-membered azobenzocrowns **113**–**118** with peripheral thiol moieties (Scheme 18) were obtained from hydroxyazobenzocrown *via* the respective bromoderivatives (**113a**-**118a**) as substrates under optimized reaction conditions [164]. The most effective preparation method of thiol derivatives turned out to be a relatively simple thiourea route giving functionalized crowns in yields 27–46%.

**Fig. 56:**
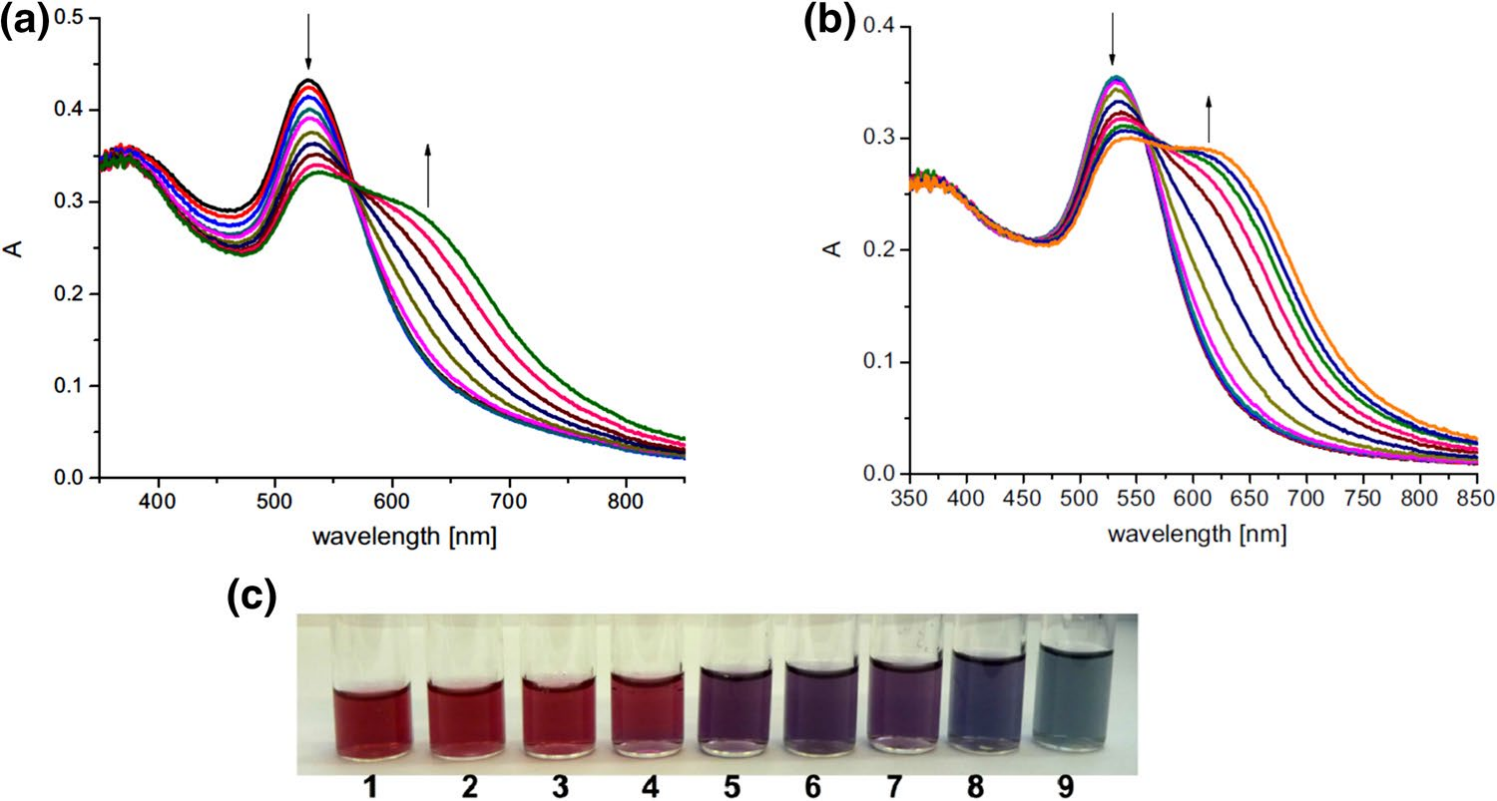
Changes in UV–Vis spectrum of: Left: Au-**116** (2.3 mL, 0.095 mM) upon titration with KCl solution (0.05 M, titration step: 0.15 mL), Right: Au-**116** (2.2 mL, 0.022 mM) upon titration with KCl solution (0.05 M, titration step: 0.1 mL). Bottom: Au-**116** solutions (1.5 mL) at concentration of 0.36 mM containing increasing aliquots of 0.05 M KCl: (*1*) 0 mL; (*2*) 0.1 mL; (*3*) 0.2 mL; (*4*) 0.3 mL; (*5*) 0.4 mL; (*6*) 0.5 mL; (*7*) 0.6 mL; (*8*) 0.7 mL; (*9*) 0.8 mL. Photograph taken 5 min after addition of KCl solution. Reprinted from [164]. Copyright 2015 with permission from Springer Publishing Company

Selected thiol derivatives were anchored onto the surfaces of Gold NanoParticles (GNPs) and studied as plasmonic sensors. The stable systems, which can be stored without change for several month were obtained by bifunctionalization of GNPs with thiol and lipoic acid. GNPs modified with 16-membered thiol derivatives and lipoic acid, showed spectral and visual response towards potassium cations in aqueous environment. In Fig. 56 the comparison of the spectrophotometric response of GNPs of different concentrations modified with thiol derivative **116** and lipoic acid (**Au-116**) towards potassium chloride is shown.

**Fig. 57:**
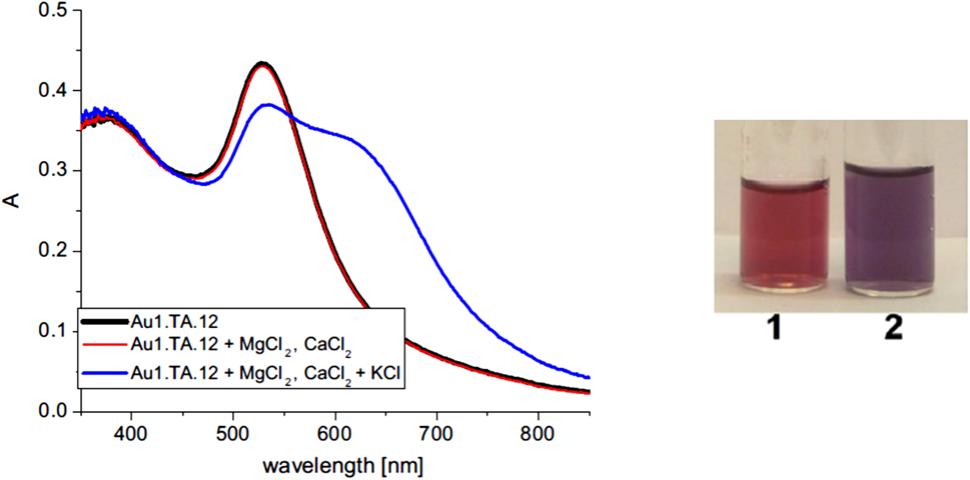
Left: Comparison of UV–Vis spectra of **Au-116** (2.3 mL, 0.095 mM) registered upon addition of mixture of magnesium and calcium chlorides (0.3 mL, 8 × 10^−4^ M) and the effect of addition of potassium chloride solution (0.6 mL, 0.05 M). Right: **Au-116** (1.5 mL) at concentration of 0.36 mM: (*1*) after addition of 0.3 mL of solution containing 8 × 10^−4^ M CaCl_2_, 8 × 10^−4^ M MgCl_2_, 1 × 10^−3^ M NaCl; (*2*) after addition of 0.3 mL of solution containing 8 × 10^−4^ M CaCl_2_, 8 × 10^−4^ M MgCl_2_ and 0.4 ml of 0.05 M KCl. Number in reproduced material corresponds to following number in this work: Au1.TA.12 = **Au-116**. Reprinted from [164]. Copyright 2015 with permission from Springer Publishing Company. (Color figure online)

The appearance of new absorption band ~ 600 nm in the presence of potassium is more distinct for diluted solutions GNPs. Color changes of **Au-116** system upon addition of potassium salt are shown in Fig. 56 (bottom). The presence of magnesium and calcium salts do not cause spectral changes, moreover, selective potassium response was found in the presence of these two ions (Fig. 57, left). Color of **Au-116** system in the presence of sodium, magnesium and calcium and color change upon addition of potassium salt is shown in Fig. 57 (right). This behavior is important from practical point of view when considering possible applications of modified GNPs in biomedical analysis.

**Fig. 58:**
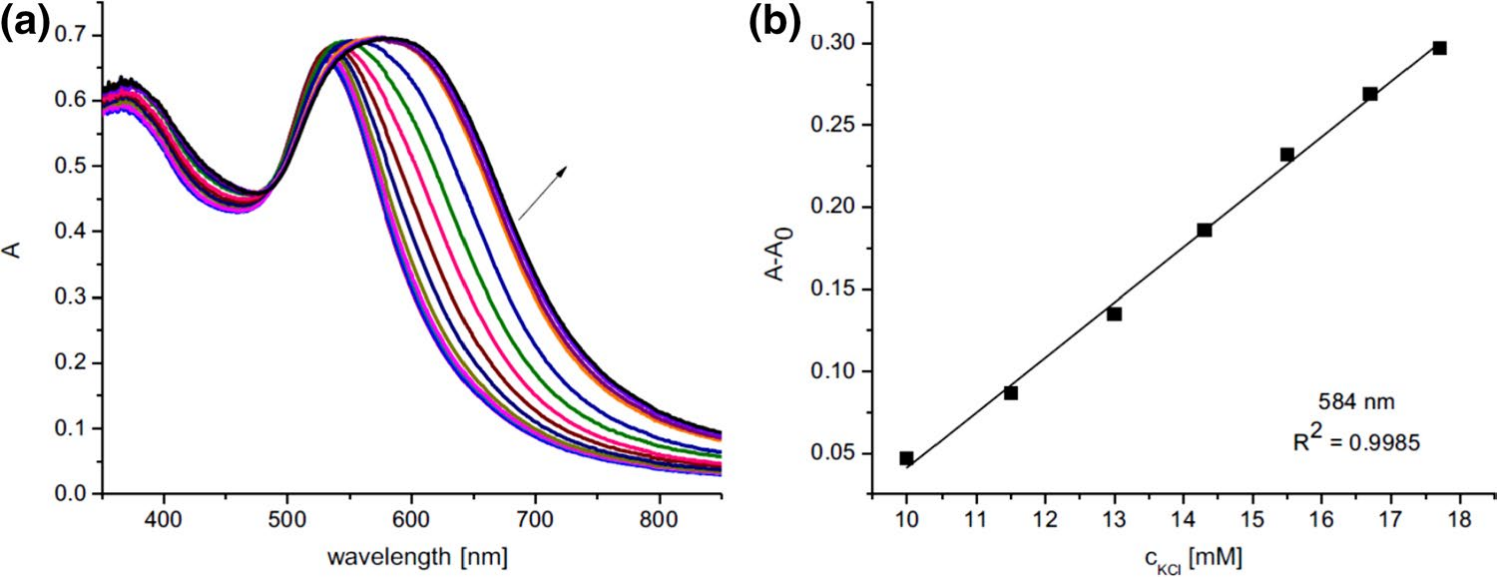
**a** Changes in UV–Vis spectrum of **Au-117** (2.0 mL, 0.25 mM) upon titration with KCl solution (0.05 M, titration step: 0.1 mL); **b** the range of linear relationship between absorbance and potassium chloride concentration (10–17.7 mM) given as a difference A–A_0_, where A is absorbance upon addition of salt and A_0_ is absorbance of **Au-117** before salt addition, at 584 nm. Reprinted from [164]. Copyright 2015 with permission from Springer Publishing Company. (Color figure online)

Among investigated 16-membered crowns the most promising results as potential potassium plasmonic sensor—regarding the stability of the system and fast response—were obtained for GNPs modified with crown **117** (**Au-117**). In Fig. 58 chages in absorption spectrum for the highest, among all tested, concentration of colloid is shown.

## Azo group(s) in macrocyclic compounds bearing azole rings

### Azo group(s) as integral part of the macrocyclic ring

**Fig. 59:**
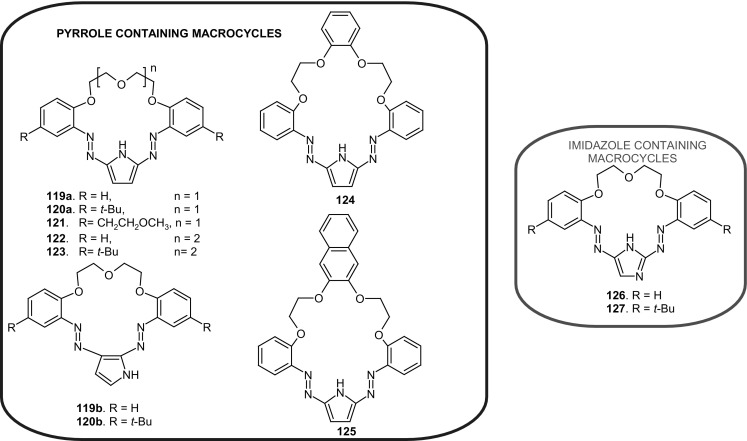
Chromogenic crown ethers with pyrrole (**119**–**125**) and with imidazole (**126, 127)** heterocyclic residue and with two azo groups in macroring [192, 193]

The first series of crown analogs, mainly 18-membered, consisting of two azo groups and heterocyclic (pyrrole or imidazole) residues (compounds **119**–**121, 126** and **127** Fig. 59) were obtained and studied since 2003 [192].

The further studies including 21-membered pyrrole derivatives (compounds **122–125** Fig. 59) showed the lead(II) selectivity of this class of compounds [193]. Complexation of lead(II) in acetonitrile solution was connected with large, bathochromic shift resulting in color change from orange to blue. High lead(II) selectivity of these compounds, over other studied metal cations, was also confirmed by experiments on transport through the liquid membranes [193]. Membrane electrodes doped with 21-membered pyrrole derived crowns **123–125** (Fig. 59) also exhibited lead(II) sensitivity with the best parameters for sensor in which lipophilic derivative **123** was used as ionophore.

**Fig. 60:**
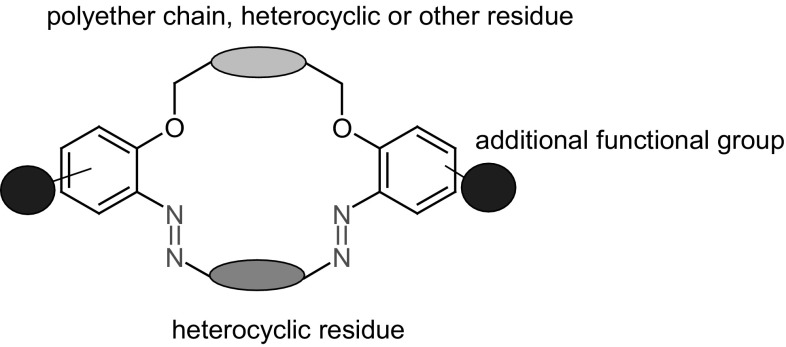
Schematically: the general formula of macrocyclic compounds with two azo groups and heterocyclic residue(s) as inherent part of the macroring

The relatively high yields of macrocyclization and attractive properties of the initially synthesized azomacrocycles with heterocyclic unit(s) motivated to expanded investigation of this class of compounds. The modification of the skeleton of macroring in different parts of the molecule (schematically drawn in Fig. 60) allowed to obtain macrocycles featuring with many interesting properties.

**Fig. 61:**
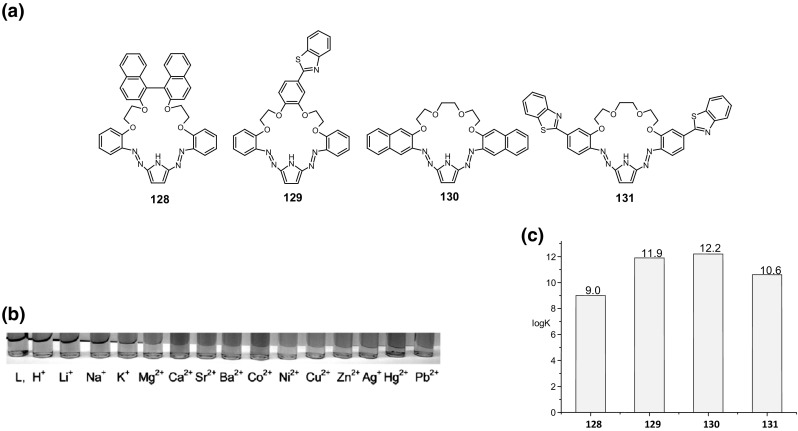
**a** Chromo- and fluorogenic macrocyclic derivatives with pyrrole residue in the macroring [194]; **b** Color changes of acetonitrile solution of **131** in the presence of the excess of metal perchlorates. Reprinted from [194]. Copyright 2011 with permission from Elsevier; **c** the comparison of the stability constants of 2:1 (L:Pb) complexes of compounds **128–131** in acetonitrile [195]. (Color figure online)

The structure of 23- and 21-membered macrocyclic pyrrole derivatives **128**–**131** shown in Fig. 61a comprise chromogenic and fluorogenic character [194]. Pyrrole containing macrocycles were prepared in multistep reactions; the final step was the azocoupling of the respective bisamines with pyrrole that proceeds with moderate yields.

Azocrowns bearing pyrrole residue **128**–**131** are lead(II) selective with the highest values of the stability constants and the most significant spectral shifts. The comparison of the color change in the presence of lead(II) and other metal cations is shown in Fig. 61b. The comparison of the stability constants of 2:1 (L:Pb) complexes of crowns **128**–**131** with lead(II) perchlorate in acetonitrile [195] is presented in Fig. 61c.

**Fig. 62:**
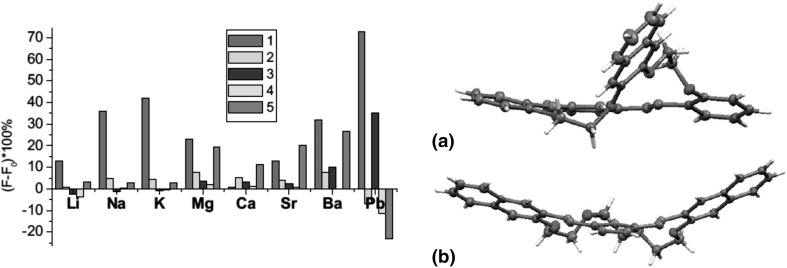
Left: the changes of fluorescence intensity for crowns **125** and **128–131** in the presence of 100-fold excess of metal perchlorates in acetonitrile. Right: the side view of the X-ray structure of the macrocycles **a 125** and **b 130**. Numbers of compounds in reproduced figure correspond to following numbers of compounds in this manuscript: 1 = **125**, 2 = **128**, 3 = **129**, 4 = **130**, 5 = **131**. Reprinted from [194]. Copyright 2011 with permission from Elsevier. (Color figure online)

The incorporation of the fluorescent moieties into the structure of azomacrocycles **125** (Fig. 59) and **128**–**131** (Fig. 61a) results in compounds for which lead(II) complexation is associated with quenching or increase of the fluorescence intensity, depending on the type of substituent. The changes of fluorescence intensity for crowns **125** and **128**–**131** in the presence of 100-fold excess of metal perchlorates in acetonitrile are shown in Fig. 62 (left).

The binding properties and associated changes in fluorescence spectra of crowns **125** and **128–131** can be to some extent explained by analyzing their X-ray structures (Fig. 62, right). Naphthalene fragment(s) influences the geometry of **125** and **130**. Particularly, the introduction of the naphthalene unit into the oxyethylene chain affects the conformation of this part of the molecule leading to the significant distortion of its shape. On the other hand the arrangement of the two naphthalene moieties in compound **130** causes molecule to be more flat, resembling a flying butterfly.

**Scheme 19:**
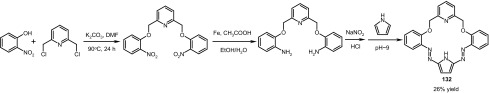
The synthesis of crown **132** with two heterocyclic residues: pyrrole and pyridine as inherent part of the macrocycle [196]

For the above-described compounds **119–125** (Fig. 59) the characteristic constituent of the structure is polyether chain coexisting with heterocyclic residue and two azo groups. More “soft” nitrogen atom donor, instead of the polyether fragment pyridine was inserted and the effect of such structure modification on metal cation binding was investigated [196]. Crown **132** (Scheme 19) constitutes of two heterocyclic residues: pyrrole and pyridine, and two azo groups forming inherent part of the macroring.

**Fig. 63:**
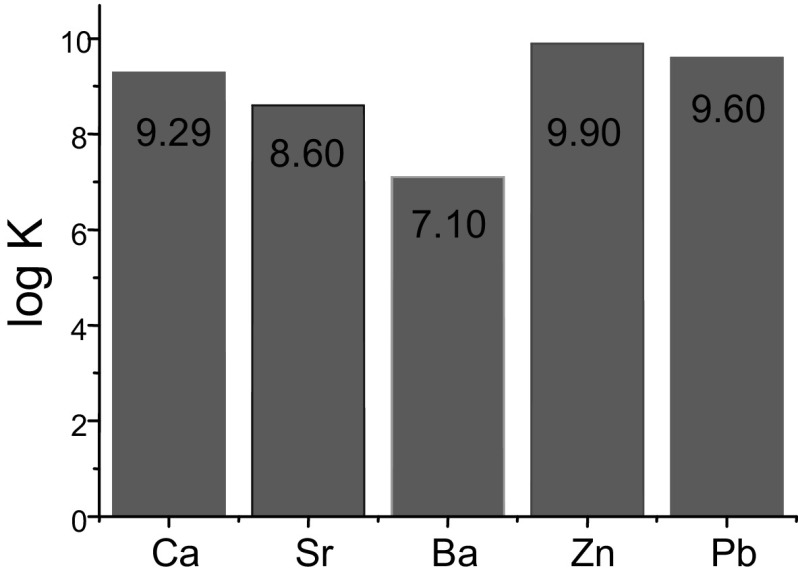
The comparison of the values of the stability constants of 2:1 (L:M) complexes of compound **132** with metal perchlorates in acetonitrile [196]

Metal cation complexation by **132** was studied using UV–Vis spectroscopy in acetonitrile and in its mixture with water. In acetonitrile, spectral and color changes were not observed in the presence of alkali metal ions and magnesium, but the presence of calcium, strontium and barium affected the absorption spectra of **132**. Among heavy metal cations in the case of nickel(II) and cobalt(II) slow kinetics of complex formation was observed. The presence of zinc(II) and lead(II) caused the most significant color changes of acetonitrile solution of **132** from red to violet. In Fig. 63 the values of the stability constants of 2:1 (L:M) complexes of **132** with metal cations in acetonitrile are presented with the highest value for zinc(II) complex. In water containing system (acetonitrile:water, 9:1), the spectral changes were observed only in the presence of lead(II) and zinc(II) salts.

**Fig. 64:**
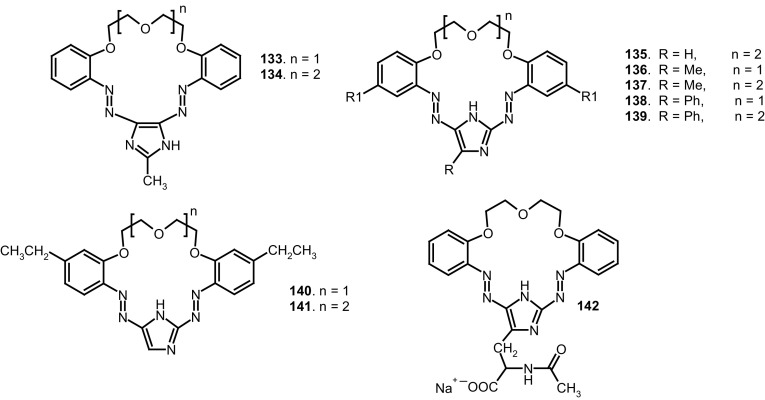
Imidazole bearing macrocylic azo compounds [197]

Besides pyrrole, also imidazole and its derivatives were used as substrates in diazocoupling reaction (reactions carried similarly to that shown in Scheme 19, under high dilution conditions) resulting in 17-(compound **133**), 18- (**136, 138, 140**), 20- (**134)** and 21-membered (**135, 137, 139, 141**) macrocycles differing in lipophilicity and substitution of the imidazole moiety (Fig. 64) in the yield up to 55% [197].

The synthesis of macrocyclic imidazole derivatives **126**, and **133–141** was also carried out in the presence of α-, β- and γ-cyclodextrins (CD) [198]. The most spectacular results were found for *γ*-CD in the case of diazocoupling of 2- and 4-methylimidazole with bisdiazonium salt. The yield of products **133** and **136** was as high as 89 and 75%, respectively. In reaction carried out without γ-CD compounds **136** and **133** were obtained in ~ 45% yield.

Besides imidazole, also 18- (**119a, 119b**) and 21-membered (**122**) pyrole derivatives were obtained by cyclodextrin assisted synthesis. The size of used cyclodextrin affects the total yield and the ratio of isomeric crowns **119a** and **119b**. The overall yield of the two macrocyclic compounds formed simultaneously reached 87% in the presence of *β*-CD. In the absence of *β-*CD the cumultative yield of both isomers is ~ 40%. Cyclodextrins assisted syntheses of azomacrocyles bearing pyrrole or imidazole confirmed the role of CDs as a molecular reactors.

**Fig. 65:**
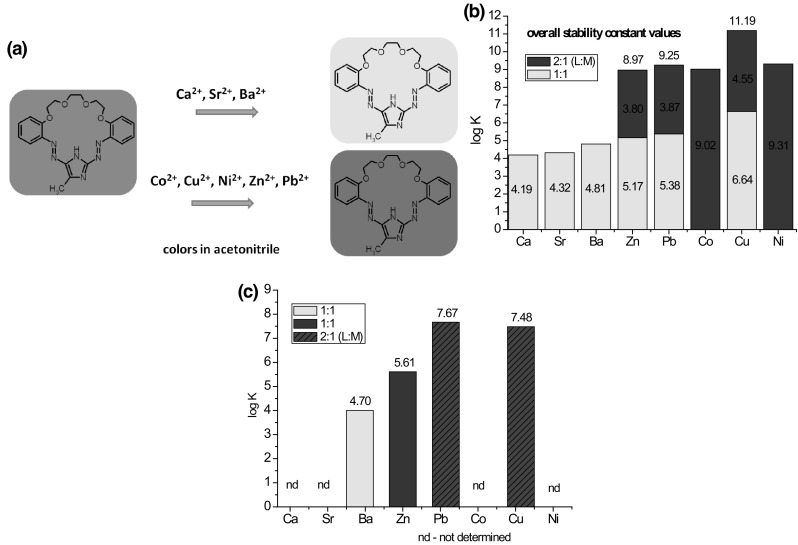
**a** Color changes of acetonitrile solution of **137** in the presence of metal perchlorates **b** the values of the stability constants of **137** complexes with metal perchlorates in: **b** acetonitrile **c** methanol [197]

Metal cations binding by 21-membered imidazole crown **137** was investigated as a model compound by UV–Vis spectroscopy in acetonitrile, methanol, and methanol–water (4:1) mixture. In acetonitrile, only calcium, strontium and barium among alkali and alkaline earth metal cations cause spectral change, i.e. hypsochromic shift, with simultaneous color changes from orange–red to yellow. In the presence of heavy metal salts the color of acetonitrile solution of **137** turns to pink-purple. Schematically, these color changes are illustrated in Fig. 65a. From spectrophotometric measurements it was concluded that complexes of **137** have different stoichiometry depending on the complexed metal cation. Alkaline earth metal ion complexes have 1:1 stoichiometry; in the case of cobalt(II) and nickel(II) 2:1 (L:M) complexes dominate. In systems containing **137** and zinc(II), lead(II) and copper(II) salts two 1:1 and the 2:1 (L:M) complexes exist under equilibrium. The values of stability constants (logK) and the most probable stoichiometry of the formed complexes are shown in Fig. 65b. It was found (^1^H NMR) that polyether chain plays a bigger role in the case of barium ion complexation than in the case of lead(II) complex, where imidazole residue was supposed to be engaged. In methanol, only barium, among alkaline earth metal cations, and zinc(II), lead(II) and copper(II), among heavy metal cations, caused significant changes in UV–Vis spectra. For barium and zinc(II) 1:1 complexes dominate, whereas lead(II) and copper(II) form complexes of 2:1 (L:M) stoichiometry. The values of the complex stability constants of **137** with metal perchlorates in methanol are shown in Fig. 65c. Comparing with acetonitrile, in methanol larger spectral shift, particularly for lead(II) complex was observed. The further increase of solvent polarity by water addition to methanol (mixture 4:1, methanol:water) resulted in increase of molar absorption coefficient of **137** and further increase of the selectivity. Only copper(II) complex with spectral shift of 55 nm and lead(II) with spectral shift of 61 nm caused spectral changes in water containing system. The values of the stability constants (logK) 4.75 and 5.51 for lead(II) and copper(II) 1:1 complexes are lower than in acetonitrile and methanol.

Macrocyclic imidazole derivatives (Fig. 64) were also tested as ionophores in membrane ion-selective electrodes. The obtained potentiometric sensors were lead(II) sensitive.

Selected macrocyclic derivatives of imidazole crowns **126** (Fig. 59), **135** and **137** (Fig. 64) were entrapped in silica xerogel matrix and tested as optical recognition elements for metal cations in water [199]. Among prepared materials, elements based on 21-membered derivatives of imidazole **135** and **137** showed spectral response (change of reflectance) in the presence of lithium salt in aqueous solution.

Structurally similar to the macrocyclic derivatives of imidazole discussed above is compound **142** (Fig. 64), *N*-acetylhistidine based macrocyle with two azo groups [200]. This is an example of crown ether containing azo group and –COOH mobile protons of unique pH controlled properties. The electrochemical behavior of imidazole derivative **126** (Fig. 59) and *N*-acetylhistidine azocrown ether **142** adsorbed on the electrode surface was investigated under different pH conditions. At pH ~ 12 similar voltammograms for **126** and **142** were obtained, but different changes were noticed under acidic conditions. It was concluded, that at pH higher than 4 the adsorbed compound **142** is present in electrochemically reducible azo form on the electrode surface. In voltammograms the first step of reduction for *N*-acetylhistidine azomacrocycle **142** was not observed below pH 4.0. This suggests that transformation to the hydrazine form occurs in chemical pathway, not by means of an electrode process. This is a unique example of pH dependent on/off process due to the presence of azo group.

**Fig. 66:**
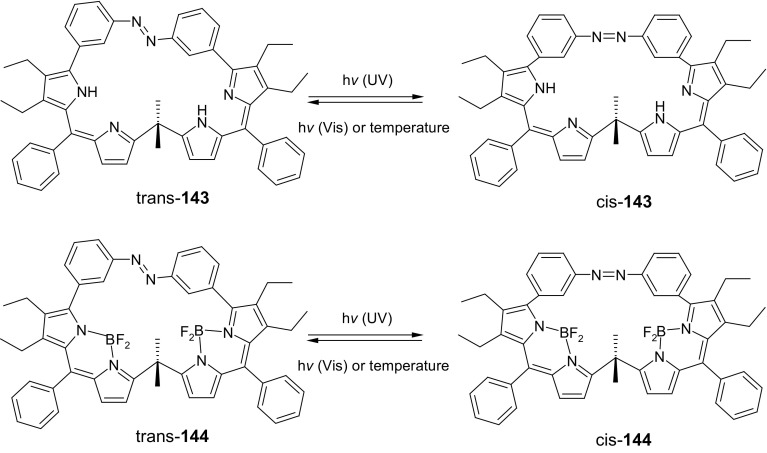
Tetrapyrrolic macrocyles **143** and **144** with azo group in macroring obtained by Tsuda and co-workers [201]

Tetrapyrrolic macrocyles with azo moiety as a part of macroring, compounds **143** and **144**, (Fig. 66) were obtained by Tsuda and co-workers [201].

^1^H NMR experiments performed in chloroform-*d* showed that after exposure of **143** solution onto UV-light (350 nm) a mixture of isomers was present. In the photostationary state the *trans* to *cis* ratio was 6:4. Higher conversion to the *cis* isomer was obtained for compound **144** with the *trans* to *cis* isomers ratio 4:6. According to theoretical calculations for *cis* isomer of **143** the photoisomerization results in a larger structural distortion of the macrocycle. Reverse *cis* to *trans* reaction for both compounds occurs thermally and can be accelerated upon irradiation with visible light. The half-life at 25 °C of thermal back reaction for **143** was estimated to be 8 days in toluene solution, whereas for **144** the time is much shorter (14 h). Taking into account the values of activation energy for **143** and **144** obtained from van’t Hoff plots it was stated that the thermal stability of *cis-***144** is lower than *cis*-**143** having less overcrowded structure. The obtained tetrapyrrolic derivatives were suggested to be used for photoswitchable molecular devices.

**Fig. 67:**
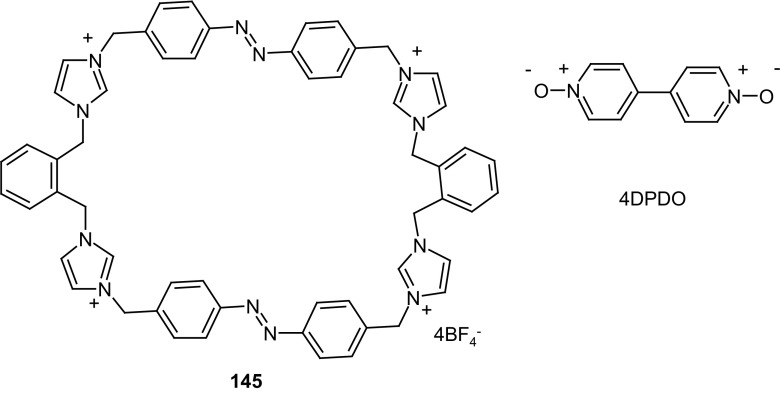
Molecular container **145** and its complementary guest—4,4′-dipyridyl-*N,N*′-dioxide (4DPDO) [202]

The reversible *trans*–*cis* isomerization of host molecules, as it was shown in relatively early works of Shinkai [141] can be used for light-driven transport of guest molecules. Scherman and co-workers [202] described the synthesis and complexing properties of macrocycle **145** (Fig. 67) containing bis(imidazolium)-azobenzene motifs.

According to ^1^H NMR analysis, compound **145** exists as all-*trans* isomer (*E,E*-**145**). UV-light-promoted isomerization generates a mixture of stereoisomers: *E,E*-**145** (18%), *E,Z*-**145** (38%) and *Z,Z*-**145** (44%). Visible light illumination causes the reversible process; the isomer *E,E*-**145** (64%) prevails in the photostationary state. Macrocycle **145** in all-*trans* form interacts with 4,4′-dipyridyl-*N,N*′-dioxide (4DPDO). According to quantum mechanical calculations, in the complex the ligand adopts cage-like conformation and the guest is encapsulated inside the host cavity. Each of the oxygen atoms of 4DPDO are hydrogen-bonded by two of the four acidic protons from imidazole rings. Exposure of the complex to UV light induces *trans* to *cis* isomerization of the macrocycle azo groups resulting in the guest release from the cavity, what was detected by ^1^H NMR spectroscopy. The studies revealed that the *Z*-predominant isomeric mixture of **145** interacts with the guest very weakly due to significant decrease of the **145** cavity size and change of the ligand conformation resulted from *trans* to *cis* isomerization. Irradiation of the mixture with visible light increases the ligand affinity to 4DPDO and ensures the guest encapsulation inside the macrocycle cavity. Thanks to these properties ligand **145** can be considered as photoswitchable molecular container.

### Azo group(s) as peripheral part of the macrocycle

**Fig. 68:**
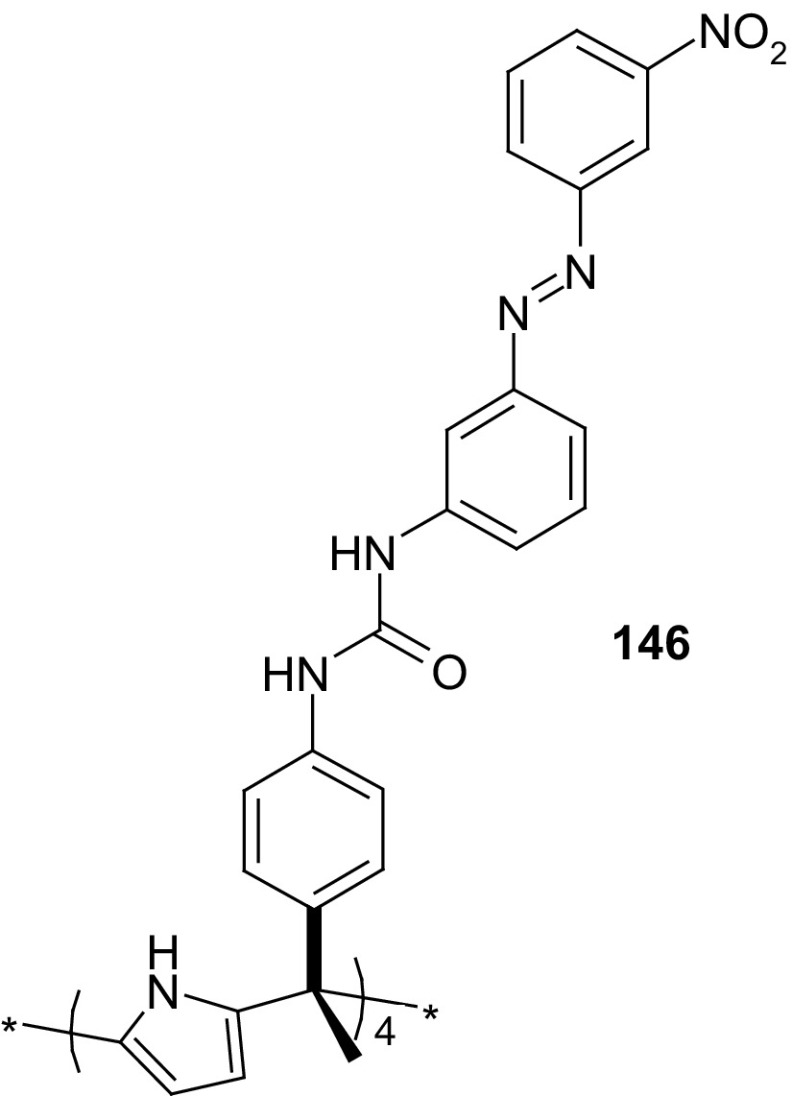
Calix[4]pyrrole ** 146** bearing azobenzene moieties described by Ballester and co-workers [203]

Thanks incorporation of azo group “spectroscopically silent” in the visible range compounds gain the chromogenic character and photoactivity. Ballester and co-workers [203] reported dimerization of urea-based calix[4]pyrole with four appended azobenzene units **146** (Fig. 68) templated by the encaplsulation of 4,4′-dipyridyl-*N,N*′-dioxide (4DPDO). The assembly can be observed only when all azo groups are in *trans* form. Light-induced *trans* to *cis* isomerization of a single azo moiety is probably sufficient to trigger the disassembly of the capsule. The subsequent back isomerization conducted in the dark or upon visible light irradiation results in the quantitative recovery of the complex.

**Fig. 69:**
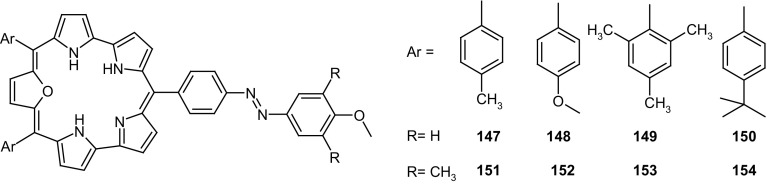
Azobenzene-smaragdyrin conjugates **147–154** [204]

Molecular switches or molecular devices can be based on porphyrins and phthalocyanines skeletons. An azobenzene moiety was joined at the meso position of expanded porphyrins, called also smaragdyrins [204]. Azobenzene-smaragdyrin conjugates were obtained using dipyrromethane bearing azo unit as precursor. Smaragdyrins **147–154** (Fig. 69) were synthesized by acid-catalyzed [3 + 2] oxidative coupling of dipyrromethane precursors with para-substituted 5,10-diphenyl-16-oxatripyrranes in ~ 25% yields. The spectroscopic and electrochemical measurements showed that the azobenzene residue covalently linked to the meso carbon atom leads to interaction between the azobenzene residue and the macrocyclic π-system. In solid state compound’ **151** azo group is in *trans* form. The reversible *trans⇄cis* isomerization for smaragdyrin–azobenzene conjugate was studied for compound **151** in toluene by irradiation (360 nm) and monitored by UV–Vis spectroscopy. In UV–Vis spectrum upon illumination the following changes were observed: a decrease of absorption intensity of band at 350 nm and some increase in the absorption band at 450 nm with a sharp decrease of the band intensity in the Soret band region. These changes were attributed to the *trans* to *cis* isomer conversion. Upon continuous irradiation within about 5 min a green color characteristic for smaragdyrin disappeared indicating decomposition of the system.

**Scheme 20:**
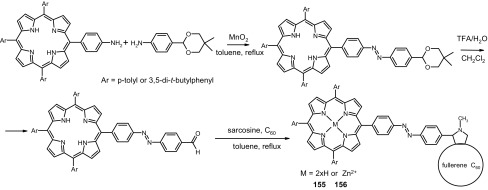
The synthesis of porphiryn-azobenzene-fullerene dyads **155** and **156** [205]

The linkage of porphyrin—both free and in form of zinc(II) complex—and fullerene C_60_ moieties (Scheme 20) with a central azobenzene moiety gave dyads **155** and **156** which properties can be controlled by photoinduced changes only of the azo moiety [205]. The properties of obtained macrocyles were exhaustively studied by spectroscopic and electrochemical methods. Unfortunately, no evidence of photoinduced *cis*–*trans* isomerization was found; moreover the photochemical decomposition of the azo dyads was noted upon continuous irradiation.

## Calixarenes with azo group

Calixarenes—cyclic oligomers are obtained by base or acid-induced condensation of phenol with aldehyde. Since the first synthesis of calixarenes by Gutsche [206], calixarenes have been used in many branches, e.g. in prevention of the oxidation of organic substances [207, 208]. However, the main applications are due to their ability to form complexes with metal cations, neutral species [209–212] and anions [213, 214]. Calix[*n*]arenes are popular host molecules, which properties can be modified towards chromogenic, photoswitchable and redox active compounds by introducing an azo moiety. Modification of calixarenes skeleton can lead to conformational and structural changes and can affect the binding properties of these macrocyclic compounds.

**Fig. 70:**
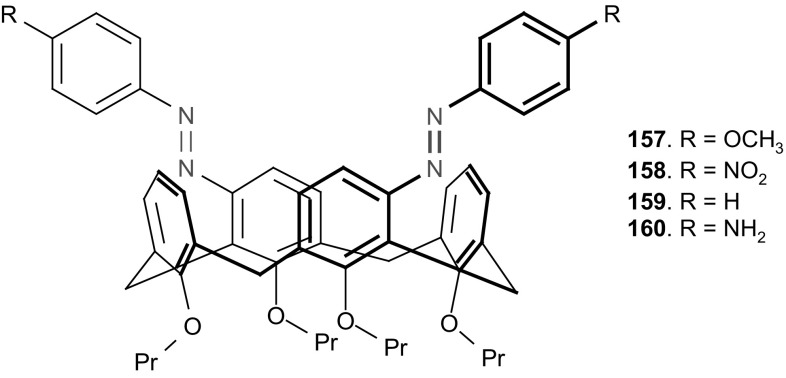
*Meta-*substituted  calix[4]arenes **157**–**160** of unusual rigidity [215]

The conformation of  calix[4]arene, i.e. *cone, partial cone* or *1,3-alternate* depends inter alia on the number and type of substituents of the macroring. Calix[4]arenes in the *cone* conformation with azo moieties at the *meta* position (in relation to the alkoxy groups) (Fig. 70) were obtained via nitroso derivatives obtained by preceded mercuration [215]. The effect of the unique for calixarenes substitution pattern and introduction of two bulky residues is rigidified molecular structure. In solution *pinched cone*–*pinched cone* interconversion is stopped (found by temperature dependent ^1^H NMR in dichloromethane), meaning that modified calix[4]arene adopts only one of the two possible pinched cone conformations. The resulting *meta* position modified calix[4]arenes can be considered as molecular receptors exhibiting cation-π binding properties.

**Scheme 21:**
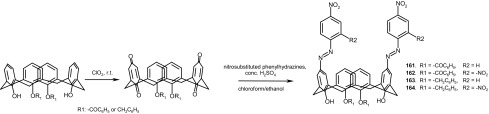
Synthesis of *O*-dialkyl  azocalix[4]arenes **161**–**164** with two free phenolic groups by diquinone route [216]

A series of  azocalix[4]arenes **161**–**164** with one, two, three, and four free phenolic groups were synthesized in reaction of 4-nitro- and 2,4-dinitrophenylhydrazines with calix[4]arene diquinones and also in diazocoupling reactions of  calix[4]arenes [216]. As an example, the synthesis of dialkyl  azocalix[4]arenes with two free phenolic groups by diquinone route is shown in Scheme 21.

The relationship between the conformation of  azocalix[4]arene and the number of free phenolic OH groups was established by X-ray studies. It was concluded that azocalix[4]arene adopts a cone conformation if it contains at least one free phenolic OH group. *Partial cone* or *1,3-alternate* conformers of  azocalix[4]arenes were found in macrocycles with all substituted phenolic groups. The possibility of the controlling the conformation of  calix[4]arenes is very important in design of the complementary hosts to a particular guest molecules. Additional benefit is the introduction of chromogenic groups useful in preparation of spectrophotometric and naked-eye sensing reagents.

**Fig. 71:**
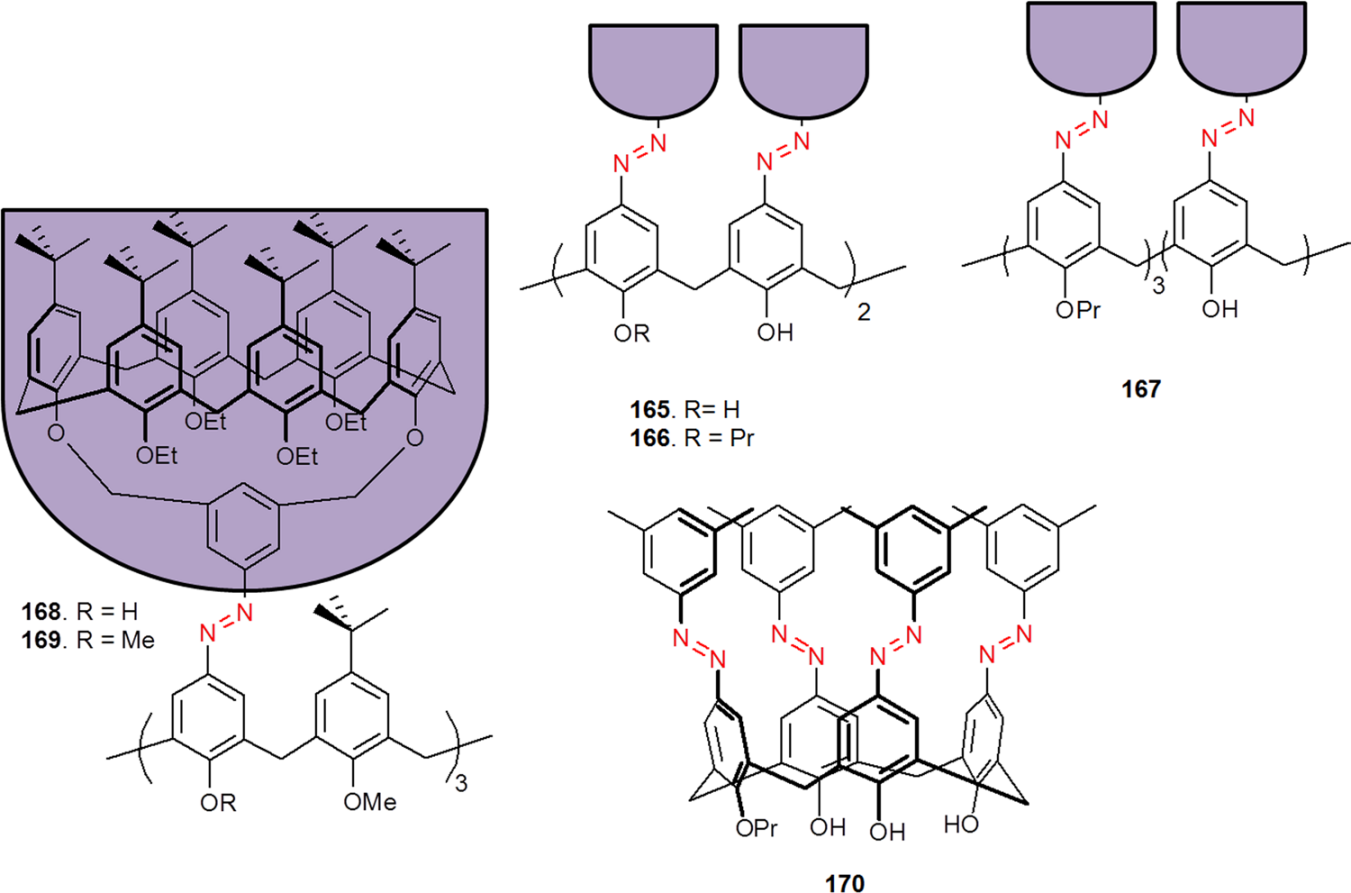
Dendricalixarenes **165**–**169** functionalized with photoswitchable azo moieties and model compound 1**70** [217]

Dendricalixarenes **165**–**169** (Fig. 71) [217] with azo groups at the upper rim of  calix[4]- or  calix[6]arene can be next examples of the structural consequences of attaching photoactive azo moieties. For preparation of azo derivatives, a method of direct diazocopuling of the diazonium salt with calixarene core was elaborated. The expected effect of the introduction of photoswitchable azo groups was to control the flexibility and accessibility of the inner spaces of the dendrimers by *trans*–*cis* isomerization. It was found that receptors **166, 167**, and **169** (with the azo groups in the *trans* configuration) do not form complexes despite the increased accessibility of the inner spaces of the dendrimers. Complex formation was not observed also upon photoinduced conversion into *cis* forms. However a model compound **170** forms a stable complex of high binding constant with 4-(4-dimethylaminostyryl)-*N*-methylpyridinium iodide (DASPMI) of rod-like geometry.

**Fig. 72:**
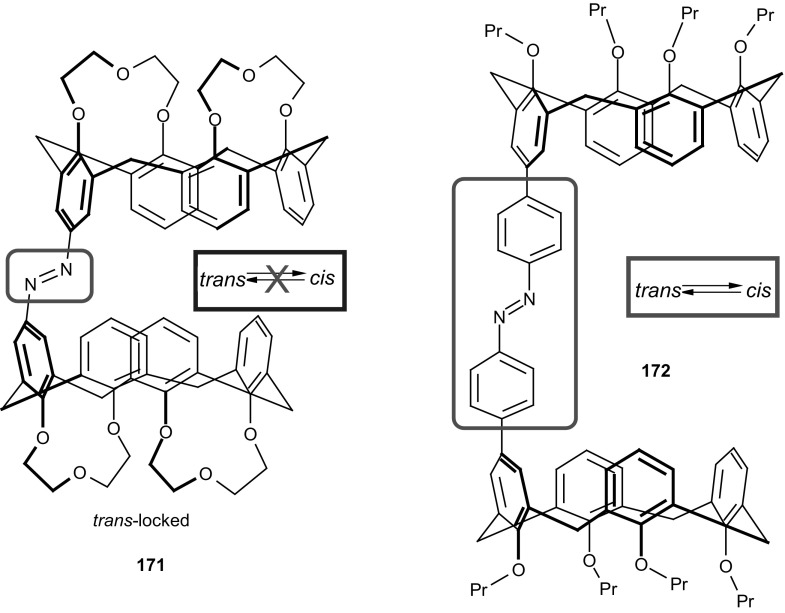
Dimeric  azocalix[4]arenes: **171**—not isomerisable, **172**—undergoing reversible *trans*–*cis* isomerization [218]

Photoactive azo residue was also used for joining two molecules of calix[4]arenes to form dimeric structures (Fig. 72) [218]. Azo linked calix[4]arene **171** exists in a locked *trans* form [219]. The modification of the structure by providing azobenzene linker between two calix[4]arene molecules results in more flexible structure, for which the reversible *trans*–*cis* isomerization can be achieved. UV illumination of **172** solution in chloroform leads to photostationary state with 35:65 *trans : cis* ratio (according to ^1^H NMR measurements). The reverse *cis*–*trans* isomerization rate of **172** in the dark, is solvent polarity, viscosity and pH dependent. These properties of dimeric calix[4]arene make it useful as a light-responsive molecular container.

**Fig. 73:**
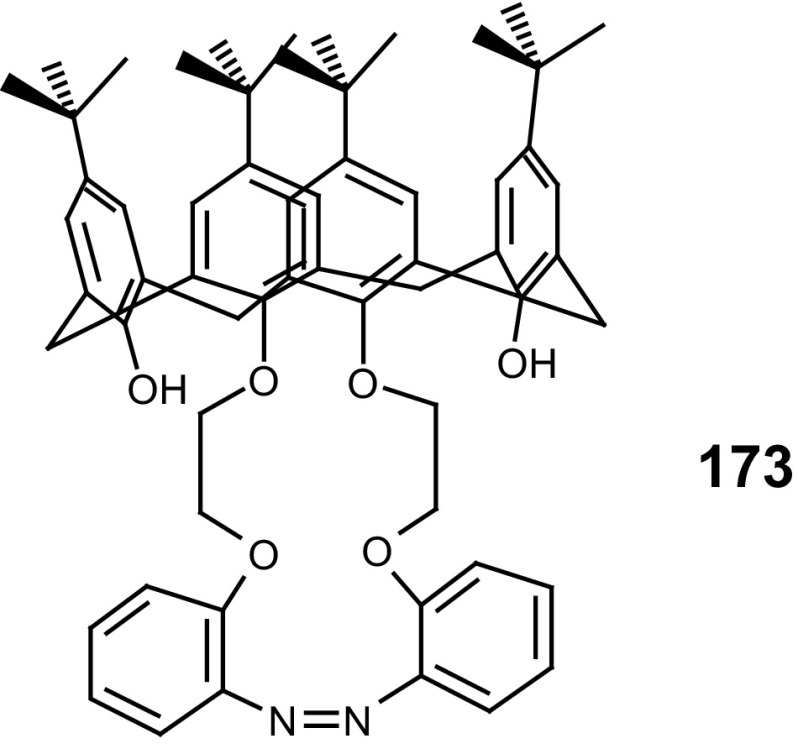
Azobenzene crown ether  *p*-*tert*-butylcalix[4]arene **173** studied analyzed by DFT calculations [220]

Calixarene skeletons with crown ether functionality form metal cation complexes with the involvement of the macrocyclic polyether unit. Complexation of metal cations by azobenzene crown ether  *p*-*tert*-butylcalix[4]arene **173** (Fig. 73) was analyzed using DFT calculations [220].

The optimized structures of crown ether *p*-*tert*-butylcalix[4]arene showed that more stable is this form in which azo moiety has *trans* configuration. Proposed models also showed that for both *trans* and *cis* isomers better complexation efficiency for alkali metal cations characterizes crown-ether with benzene-rings (*exo*) pockets. The obtained models also proved the higher affinity of macrocycle for sodium over potassium ion. Preferential sodium cation complexation was found for the *trans*-complex in the *exo*-binding mode comparing the *cis-exo* analogue.

**Fig. 74:**
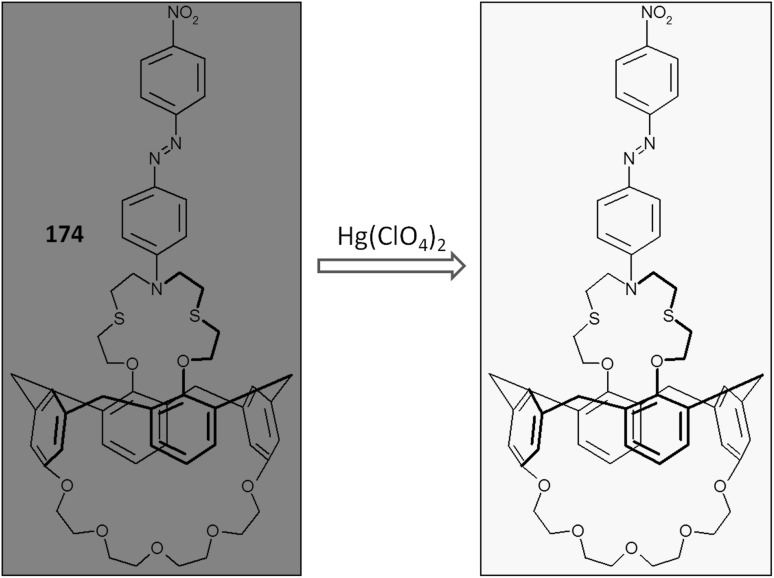
Calix[4]biscrown **174** sensitive to mercury(II) perchlorate as chromogenic sensing molecule [221]

 Calix[4]biscrown compound bearing azobenzene unit **174** was described as a colorimetric receptor for Hg^2+^ ions in acetonitrile (Fig. 74) [221]. According to UV–Vis analysis it was stated that complexe of 1:1 stoichiometry  is formed. Complexation is manifested by change of color from red to pale yellow only when perchlorate anions are used as the counterion. X-ray analysis of Hg(NO_3_)_2_-**174** complex shows crystal structure with the formula [Hg(**174**)(NO_3_)_2_]_n_ where each of *exo*-coordinated Hg^2+^ is in a distorted tetrahedral surroundings with its coordination sites occupied by two sulfur atoms from two different ligand molecules and two NO_3_^−^ anions. Addition of potassium salt to Hg^2+^-**174** solution in acetonitrile affects its UV–Vis spectrum indicating formation of heterodinuclear complex.

**Scheme 22:**
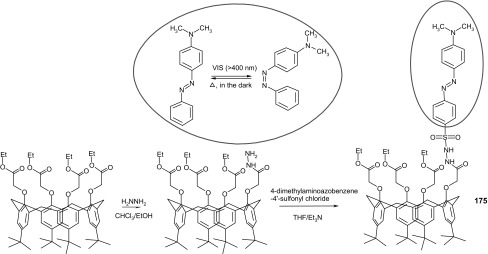
Synthesis of a photoresponsive ion carrier **175** based on calix[4]arene [222]

Supramolecular systems, where ionic species are transported across bilayer lipid membranes are used as artificial models for natural photo-excitable membranes. It was demonstrated that ether derivative of *p*-*tert*-butylcalix[4]arene bearing photoresponsive dimethylaminoazobenzene moiety **175** (Scheme 22) - acts as ion carrier in visible light (> 400 nm) driven transport of sodium cations across lipid bilayer (soybean phospholipids) [222].

**Fig. 75:**
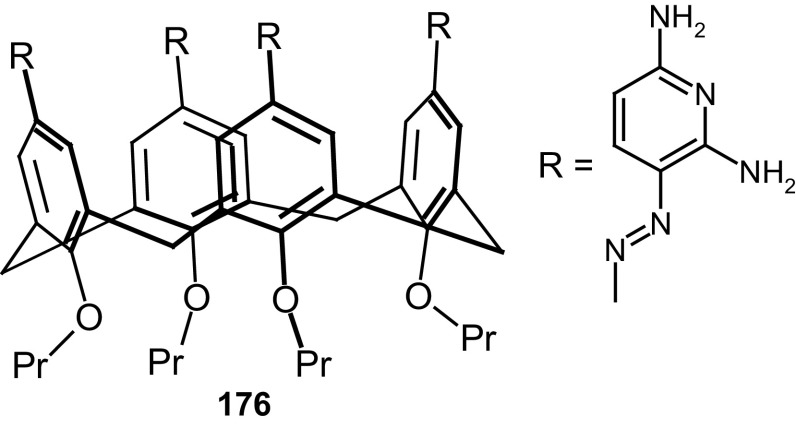
Azocalix[4]arene showing antibacterial activity [223]

Macrocyclic compounds with azo groups are interesting objects not only as molecular receptors or building blocks in molecular devices or machines. Some of them show antibacterial activity. An example can be azocalix[4]arene **176** (Fig. 75) obtained by diazo-coupling of tetradiazonium salt of calix [4] arene and 2,6-diaminopyridine, which shows strong activity against Gram-positive bacteria *Bacillus cereus*, while the activity against *Escherichia coli* is mild; the *Pseudomonas aeruginosa* shows resistance [223].

## Cyclodextrins with anchored azo moiety

**Fig. 76:**
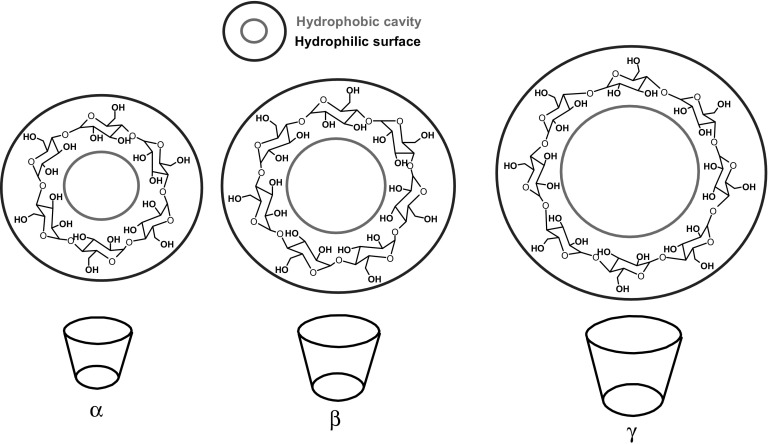
α-, β- and γ-CD and schematically their hydrophilic surface and a hydrophobic cavity

Cyclodextins (CDs) known since the Villiers discovery [224] for more than 125 years, are cyclic oligosaccharides that consist of d-glucopyranose units linked by α-(1,4)-glycosidic bonds. Depending on the number of glucopyranose units CDs can be classified as α-, β- and γ-CD with six, seven and eight units, respectively (Fig. 76).

Hydrophilic outer surface and a hydrophobic cavity of the native CDs and their derivatives cause that these compounds form inclusion complexes with a number of guest molecules: organic, inorganic, biological molecules and ions [225]. The versatile career of CDs is due to their availability, biocompatibility, biodegradability and industrial scale production. That is why complexes of CDs with various molecules have found multitude applications in many branches of science and industry, for example: in supramolecular polymers [226–228], hydrogels [229], bioactive materials [230], drug delivery [231–234], dynamic materials [235], catalysis [236] and separation methods [237–239]. Substituted cyclodextrins give the possibility of the more specific modification of different type of substrates, molecules and surfaces [240], changing their physical and chemical properties, which opens the way to the new applications of such hopeful systems [241]. Among them, derivatives bearing azobenzene moiety at various locations of the cyclodextrin(s)’ containing architectures, are intensively studied due to their chromogenic character and unique photo-responsive properties induced by the photochemical *trans*–*cis* isomerization.

**Fig. 77:**
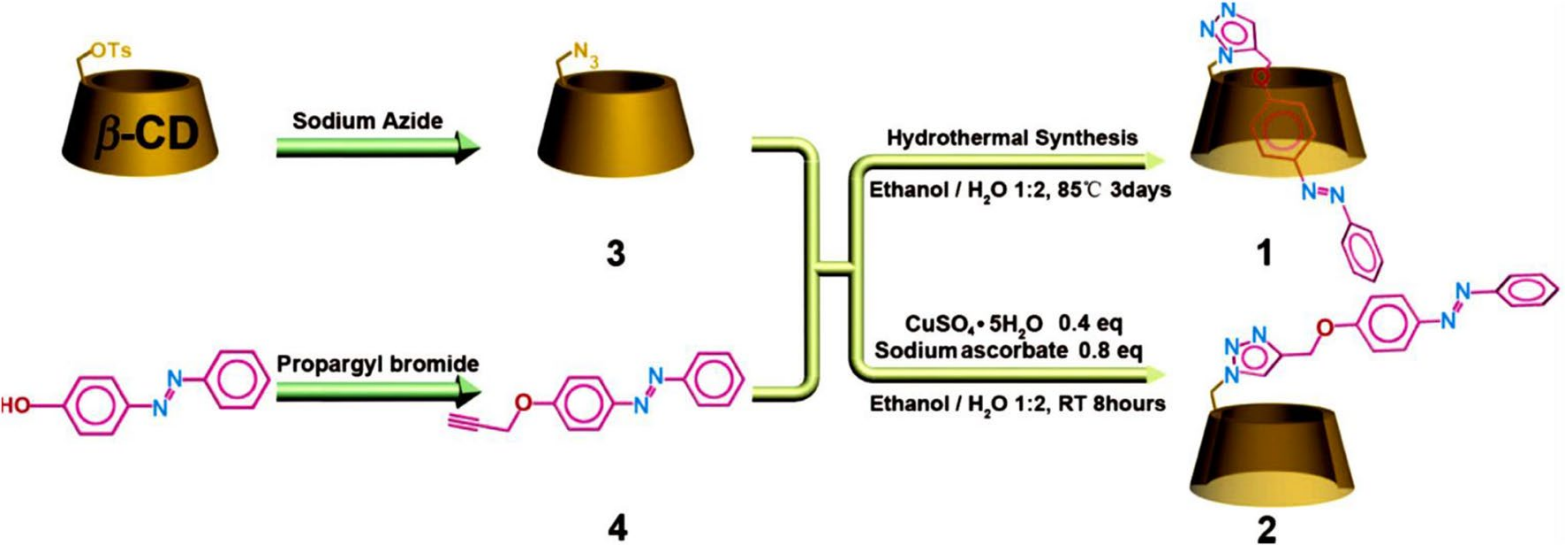
The scheme of Huisgen’s cycloadditions carried out under diverse conditions leading to different conformers **177a** and **177b**. Numbers of molecules in reproduced material correspond to following numbers in this manuscript: 1 = **177a**; 2 = **177b**. Reprinted with permission from [242]. Copyright 2008 American Chemical Society

Cylodextrins are often utilized in a construction of the nanometer-scaled supramolecular architectures. The properties and the organization of self-assemblies can vary, inter alia, depending on reaction conditions. This allows to control the structure of supramolecular frameworks by tuning the conformation of the building blocks. The interesting example of systems of the same chemical composition, but different conformations was given by Liu and co-workers [242]. In Huisgen’s cycloaddition, (Fig. 77) using the same reactants, but carrying out the reaction under different conditions two conformations: **177a**—self-locked and **177b**—self-unlocked were identified. The structures of obtained products were confirmed by X-ray and spectroscopic studies. In self-locked conformer—in solution and in solid state—azobenzene moiety is located in the own cavity. It presents a unique  [1]rotaxane without a stopper part. Conformer **177b** was found to exist as a linear supramolecule.

**Fig. 78:**
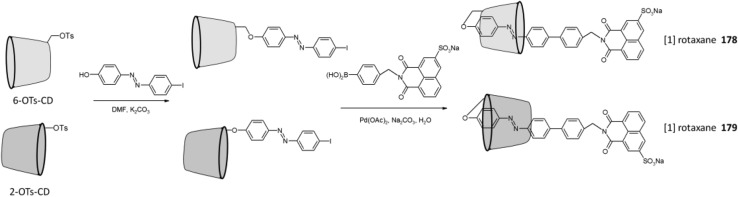
Disparate oriented  [1]rotaxanes **178** and **179** bearing azo moiety [243]

[1]Rotaxanes **178** and **179** (Fig. 78) based on β-cyclodextrin skeleton, bearing azo moiety were also efficiently prepared via self-inclusion complexation and Suzuki-coupling capping in aqueous solution [243]. Obtained [1]rotaxanes **178** and **179** were characterized by absorption and induced circular dichroism spectra. [1]Rotaxane **179** undergoes photo- and thermal reversible *trans*–*cis* isomerization.

**Fig. 79:**
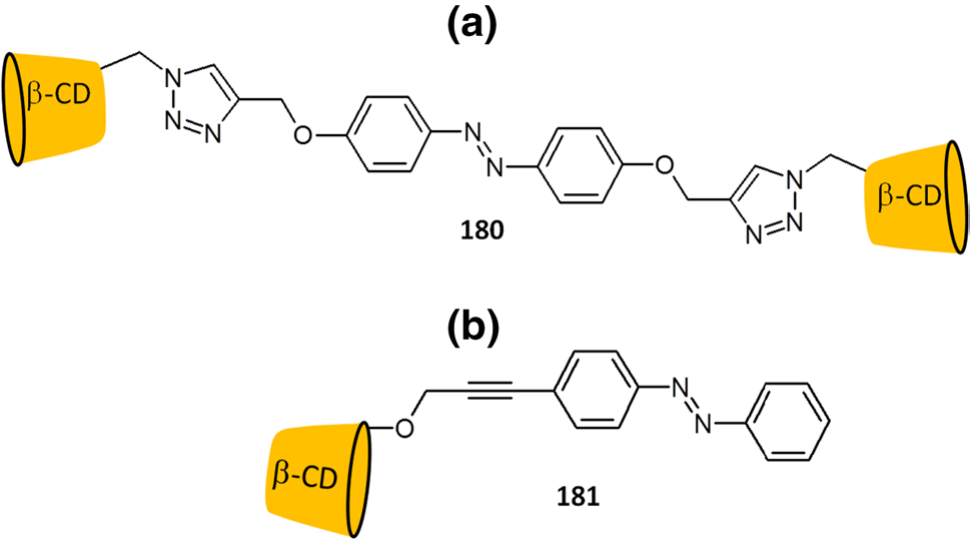
**a** Azobenzene bridged β-cyclodextrins **180** [244]; **b** β-CD modified on a secondary faced with azobenzene **181** [245]

The goal of the introduction of azo moiety while modifying the cyclodextrins is the possibility of using them as photoswitchable systems. The efficient modification of the cyclodextrins’ skeleton requires the elaboration of the appropriate synthetic procedures. Huisgen’s [3 + 2] cycloaddition was used for the efficient synthesis of azobenzene bridged β-cyclodextrin **180** (Fig. 79a) which was obtained in a click reaction in 72% yield [244].

An oxidative coupling in Sonogashira-type reaction has been used in synthesis of β-cyclodextrin derivative bearing an azobenzene group on the secondary face **180** (Fig. 79b) [245]. Optimized reaction conditions (degassing of the reaction mixture, the use of [Pd(PPh_3_)_4_], pre-heated oil bath, diluted reductive H_2_ atmosphere) allowed to diminish the formation of the dimeric side product. Such conditions resulted in 62% yield of the demanded azocompound.

**Fig. 80:**
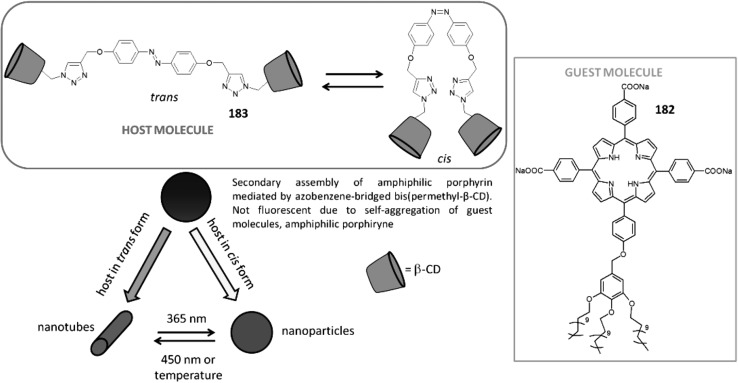
Schematic presentation of host - azobenzene bridged β-CD **183** and guest - amphiphilic porphyrin molecule **182**, and the secondary assembly and its transformations [246]

Not only assembly/disassembly processes of supramolecular systems can be photocontrolled, but also morphological transformations of supramolecular assemblies can be light-induced. Liu and co-workers [246] described the nanotube–nanoparticle morphological conversion for the secondary assembly of amphiphilic porphyrin (**182**, guest molecule) mediated by azobenzene-bridged bis(permethyl-β-CD) (**183**, host molecule; Fig. 80). Azo-bridged β-CD, both as *trans* and *cis* isomer, forms complexes of 1:1 stoichiometry with guest molecule, porphyrin **182**. The estimated binding constant value for complex of *cis*-isomer is higher than for *trans* isomer. This can be a result of cooperative binding of the porphyrin derivative by two *β*-cyclodextrin cavities in *cis*-complex (the sandwich type complex). The interaction of *trans*-azobenzene-bridged bis(permethyl-*β*-cyclodextrin) **183** with amphiphilic porphyrin derivative **182** in aqueous solution (pH 7.2) leads to aggregate formation with hollow tubular structure. According to TEM images the average inner and outer diameters of obtained nanotubes were about 45 and 61 nm with a wall thickness of about 8 nm. It was deduced that interior and exterior surfaces of nanotubes are composed of units of *trans*-**183-182** complexes, whereas the alkyl chains of **182** interlace with each other in the middle of tubular walls. UV-light irradiation (at 365 nm) of *trans*-**183-182** solution induces morphological conversion being a result of *trans* to *cis* isomerization of the azobenzene moiety. TEM analysis confirmed that upon photoisomerization long *trans*-**183**-**182** nanotubes turn to nanospheres of average diameter of 180–220 nm. Subsequent irradiation of complex with the *cis* isomer enables nanotubes reconstruction. This photocontrolled process was found to be reversible and repeatable. TEM and DLS experiments confirmed that morphological switching can be repeated by irradiation for tens of times.

**Fig. 81:**
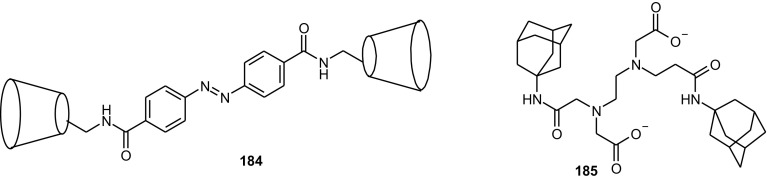
Host molecule **184** and complementary to it guest **185** [247]

Photochemical properties of bis-*β*-cyclodextrin bearing azobenzene unit **184** was reported by Djedaini-Pilard and co-workers [247]. It was found that **184** can form two different inclusion complexes with adamantyl derivative **185** depending on **184** photoinduced isomers (Fig. 81). The *cis*-**184** complex of 1:1 stoichiometry is created when two cyclodextrin cavities bind simultaneously two adamantyl units of the guest. It was deducted that formation of supramolecular polymers with *n:n* stoichiometry for *trans*-**184** is the most probable.

**Fig. 82:**
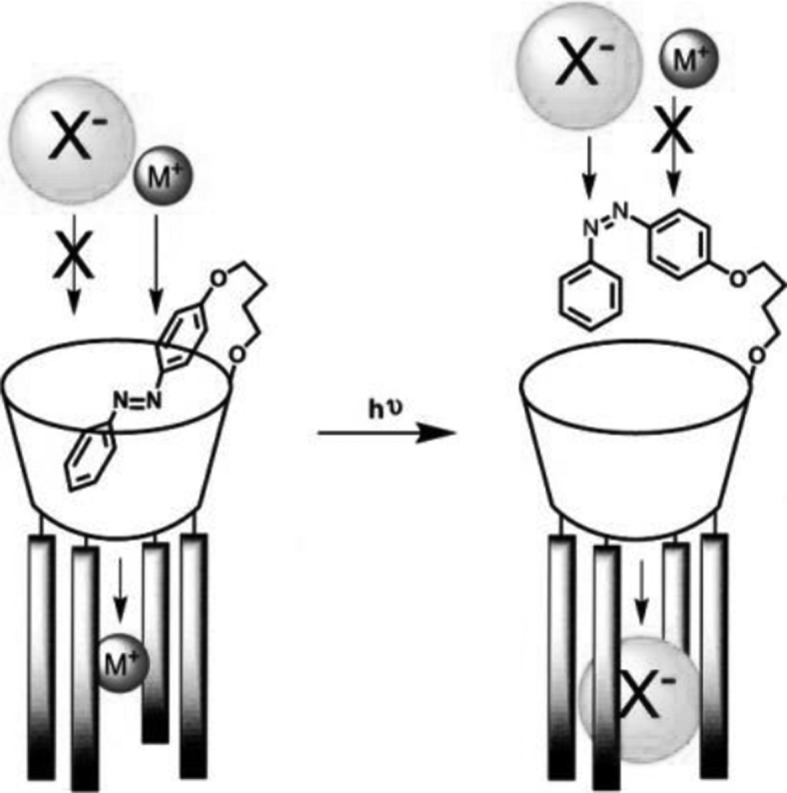
A gate synthetic ion channel based on cyclodextrin as a channel and azobenzene moiety as a gate. Reprinted with permission from [248]. Copyright 2008 American Chemical Society

Azobenzene attached to the secondary face of β-CD was utilized as gated synthetic ion channel (Fig. 82) [248]. β-CD works as a channel for ionic species transport, whereas photoresponsible azobenzene unit acts as a gate, opening or closing upon irradiation. Dependent on *trans* or *cis* conformation of azobenzene moiety, cations or anions are preferentially transported.

**Fig. 83:**
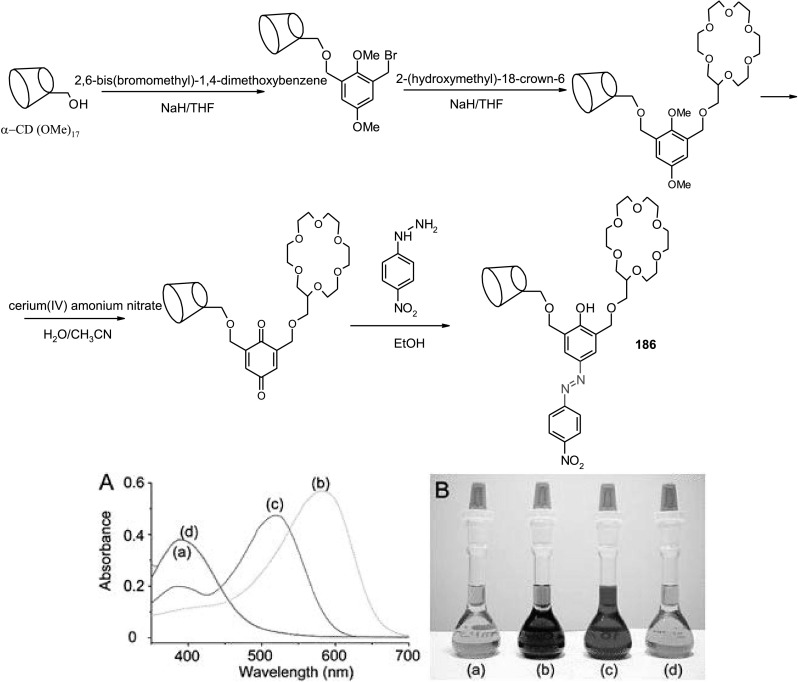
Top: Synthetic procedure for **186**, bottom: **A** UV–Vis spectra and **B** photographs of **186** (0.03 mM) with amines (1000 equiv) in CHCl_3_: **a 186, b 186** + *n*-octylamine, **c 186** + di-*n*-butylamine, and **d 186** + tri-*n*-butylamine. Reprinted with permission from [249]. Copyright 2006 American Chemical Society

Permethylated α-cyclodextrin-6^A^-monoalcohol modified with azophenol **186** (Fig. 83) was used [249] for colorimetric detection and differentiation of primary, secondary and tertiary amines. Chloroform solutions of **186** change color in the presence of 1° and 2° amines as a consequence of spectral shift from 380 to 580 for primary and to 530 nm for secondary amines. Tertiary amines do not cause spectral changes. The values of the stability constants of **186** complexes with primary amines were found to be higher than with secondary amines. It was deducted that the number of possible hydrogen bonds formed between the oxygen atoms of crown ether and amine protons is crucial for the binding strength of the guest molecule.

**Fig. 84:**
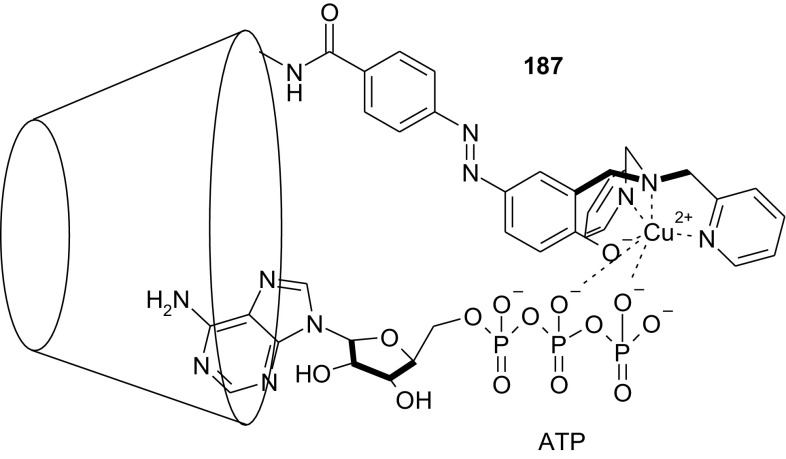
Complex of cyclodextrin derivative **187** with ATP [250]

Azobenzene bearing γ-cyclodextrin derivative **187** (Fig. 84) was tested as a probe for derivatives of phosphoric acid [250]. It was proved that the described host molecule reveals high selectivity towards ATP over other tested phosphoric acid derivatives (mono-, pyro-, and triphosphate, AMP, ADP) in aqueous solution at pH 7.4. Compound **187** forms with ATP complexes of 1:1 stoichiometry with binding constant 6640 ± 890 M^−1^. The estimated value is 2.5 times higher than for complex without cyclodextrin unit, what indicates significant role of macrocyclic cavity in the guest complexation. On the basis of ^1^H NMR experiments it was suggested that adenine moiety of the guest is bound inside cyclodextrin cavity, thanks to what it is in close proximity to the azobenzene unit making π–π interactions between adenine and azobenzene stronger than in the case of ligand without cyclodextrin residue. Additionally, recognition of phosphoric moieties of ATP is provided by dipicolylamine—Cu^2+^ unit. These multipoint interactions are probably responsible for high selectivity of ATP recognition.

Photoswitching properties of azobenzene make it an interesting candidate for controllable drug therapy. For example, azobenzene units were used in fabrication of a triple-layer nanocomposites tested in vitro anticancer therapy as a drug delivery system [251]. The single particle consists of gold nanobipyramids (the core), mesoporous silica nanoparticles (the middle layer), and hyaluronic acid functionalized with *α-*cyclodextrin and azobenzene. Inside the silica pores anticancer drug—doxorubicin is loaded. Experiments carried out for human squamous carcinoma cells (representative cancer cells) and human keratinocyte cell (representative normal cells) revealed that these nanocomposites are able to specifically accumulate around the tumor tissue due to noncovalent interactions between hyaluronic acid and CD44 receptor overexpressed in cancer cells. Localized irradiation with near-infrared light (780 nm) converts *cis*-azobenzene to its *trans* isomer what leads to hydrogel formation due to noncovalent interactions between *α-*cyclodextrin and *trans*-azobenzene. Thanks to the presence of specific enzyme—hyaluronidase around the tumor cells the network in the hydrogel is degraded resulting in the anticancer drug release and its transport to the cancer cell nuclei.

**Fig. 85:**
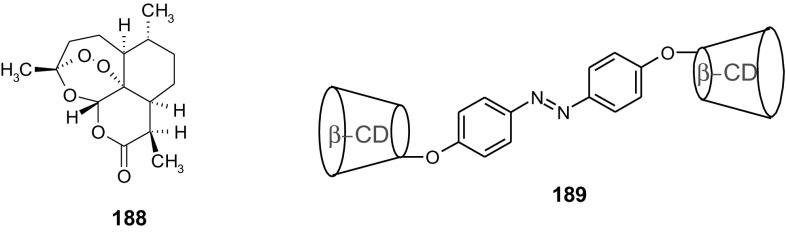
Azobenzene bridged bis(β-CD) **189** and the formula of artemisinin **188** [252]

The association of artemisinin (ART) **188** with an azobenzene bridged bis(β-CD) derivative with an azobenzene 6–6′ linker **189** in aqueous solution was investigated by circular dichroism (CD) spectroscopy (Fig. 85) [252].

It was shown that bis(β-CD) with *trans*-azobenzene unit binds artemisinin (1:1 complex) and this process can be light controlled. Upon irradiation at 363 nm *trans*–*cis* isomerization causes loss of the binding ability of artemisinin.

## Polymers bearing macrocycle(s) and azo motif(s)

Azo derivatives found a vast range of applications in polymer science. Polymers containing azobenzene moiety have been intensively studied due to their photoresponsive properties ensuring the obtainment of functional materials. An azo group can be a part of a supramolecular system in polymer matrix (non-covalent interactions) or can be covalently bound within a polymer chain. Polymers, responding to light irradiation are widely investigated systems due to reversible (or irreversible) changes of physical properties [253]. This can be utilized in many branches of science.

The change of the polymer properties can be very often achieved by using molecules, which act as molecular containers. Elegant molecules of such properties are cyclodextrins, described ealier, which can be also used for design and synthesis of functional polymers.

**Fig. 86:**
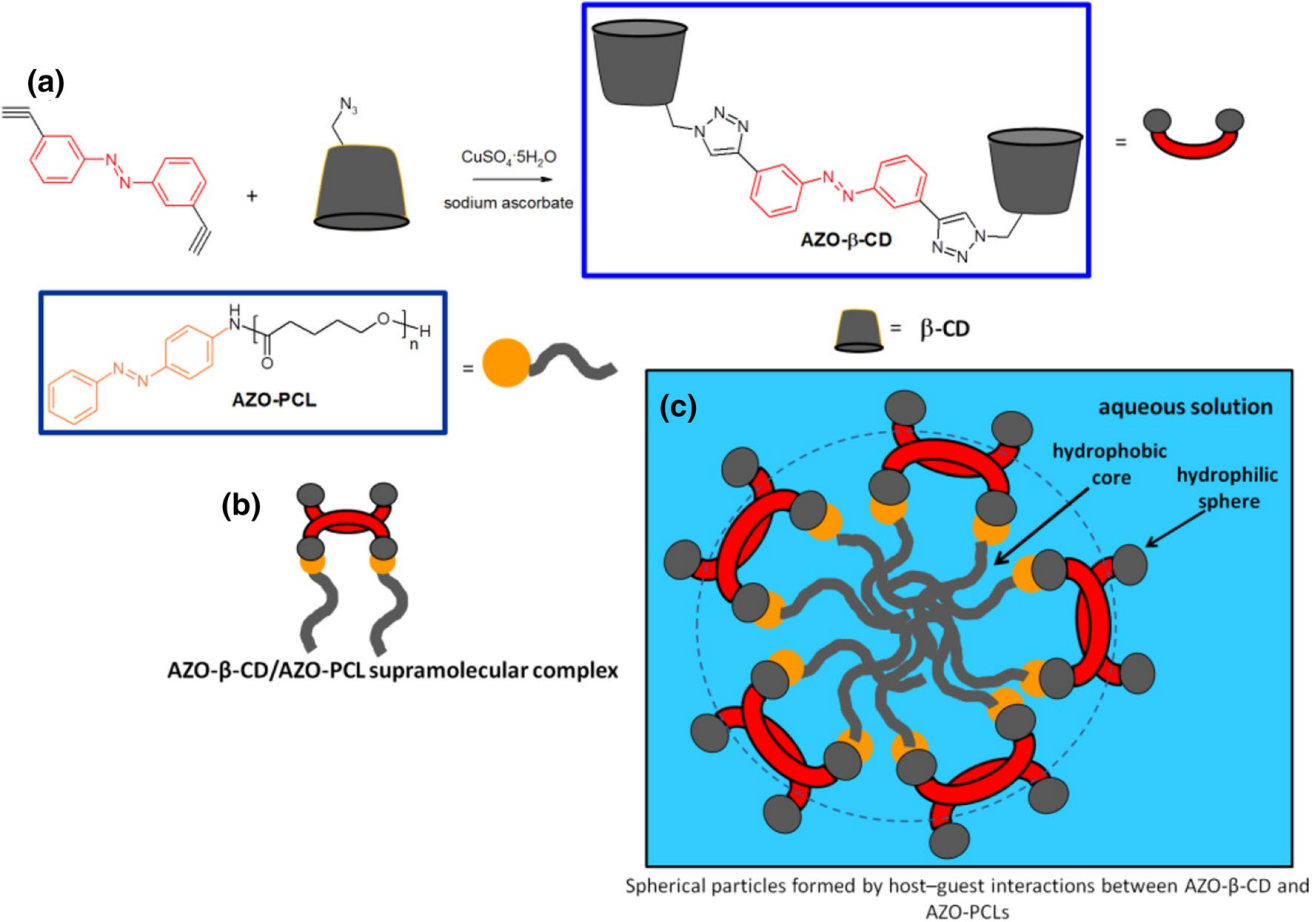
**a** The synthesis of AZO-β-CD by click reaction, **b** AZO-β-CD/AZO-PCL supramolecular complex, and **c** the possible aggregation mode: spherical particles are formed by host–guest interactions between AZO-β-CD and AZO-PCLs [254]

A system utilizing noncovalent interactions between synthesized in a click reaction AZO-β-CD (Fig. 86), which interacts as a “dimer” with azo bearing polyester obtained in reaction of ε-caprolactone with *p*-aminoazobenzene (AZO-PCL) (Fig. 86a), was described by Ma et al. [254]. It was suggested that in aqueous solution micellar aggregates are formed due to host–guest interaction between (AZO-β-CD) and AZO-PCL (Fig. 86b). On the basis of ^1^H NMR spectra it was suggested that the guest molecule in its *trans* form is included shallowly into cyclodextrin cavity from its wider site. After UV-light irradiation the transparent opalescence solution becomes turbid, what is a result of decomplexation followed by disaggregation. The uniform vesicles are reformed upon exposure of the solution to visible light. Authors propose possible use of the system in the control or release of drugs.

**Fig. 87:**
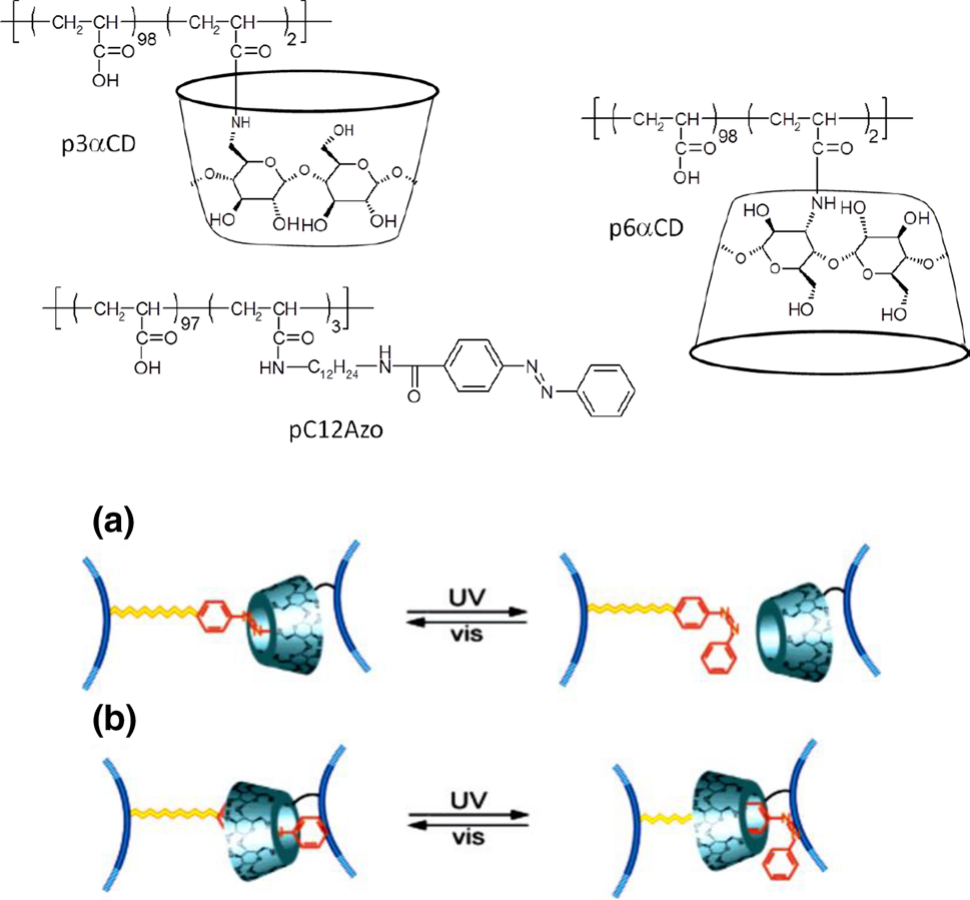
Top: p3αCD and p6αCD and pAA carrying azobenzene moieties (pC_12_Azo) used for studies of self-assembly. Bottom: schematic representation for interactions of CD and azo moieties upon irradiation with UV and visible light for (**a**) p3αCD/pC12Azo, (**b**) p6αCD/pC12Azo. Reprinted with permission from [256]. Copyright 2006 American Chemical Society

Photosensitive hydrogel based on α-CD, dodecyl-modified poly(acrylic acid), and a photoresponsive competitive guest [255] inspired further studies of self-assembling systems with polymer side chains. Poly(acrylic acid)s (pAA) with p3αCD and p6αCD functionalities and pAA carrying azobenzene moieties (pC12Azo), were used for the construction of the photoresponsive system based on polymer–polymer interactions (Fig. 87) [256]. The properties of obtained systems were studied in details among others by steady-shear viscosity (η) measurements. The method was chosen because the interaction of the CD polymers with pC12Azo (formation of inclusion complexes of CD moieties in the CD polymers with side chains of guest polymers) may cause an increase of solution viscosity. The mixture of the p3αCD/pC12Azo and p6αCD/pC12Azo has shown contrast η changes upon photoirradiation: decrease in the case of the p3αCD/pC12Azo mixture, and increase of η for p6αCD/pC12Azo mixture. Irradiation with visible light causes the reverse process in the above cases, i.e. η values became similar to those before the UV exposure. The differences in η values were explained by the fact that UV light causes dissociation of inclusion complexes for the p3αCD/pC12Azo mixture, and the formation of interlocked complexes for the p6αCD/pC12Azo mixture (Fig. 87, bottom).

**Fig. 88:**
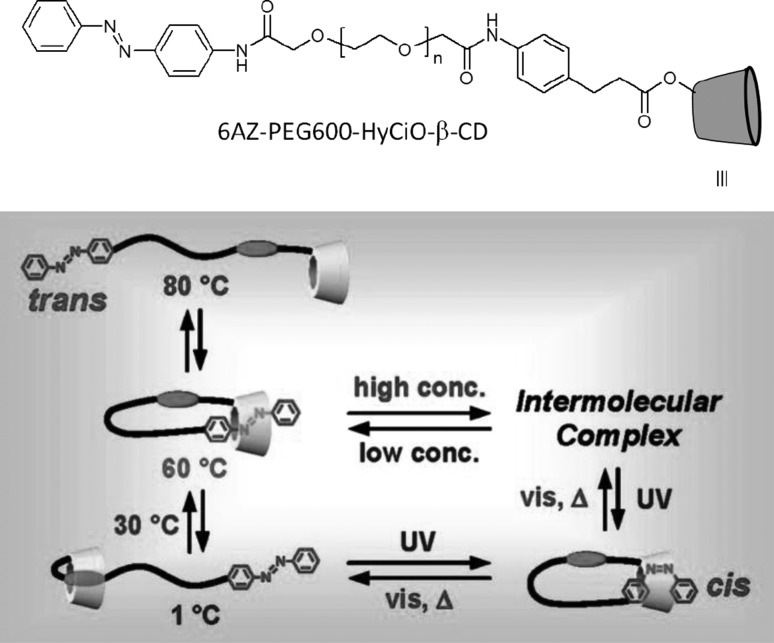
Top: 6-Az-PEG600-HyCiO-β-CD, Bottom: proposed conformational changes of 6-Az-PEG600-HyCiO-β-CD in aqueous solutions by external stimuli. Reprinted with permission from [257]. Copyright 2007 American Chemical Society

PEG-substituted CD with an azobenzene residue at the end of the PEG chain (6-Az-PEG600-HyCiO-β-CD) was obtained by Harada and co-workers (Fig. 88) [257]. The photochemically and thermally induced conformational changes in aqueous solutions were studied by 1D and 2D NMR analyses. It was found that at low concentration, 6-*trans*-Az-PEG600-HyCiO-β-CD forms different types of intermolecular, self-inclusion complexes or exists in an uncomplexed form depending on the temperature. An intermolecular complex is formed at high concentration. Regardless of the concentration, irradiation by UV light promotes complexation with the CD including the azobenzene part.

**Fig. 89:**
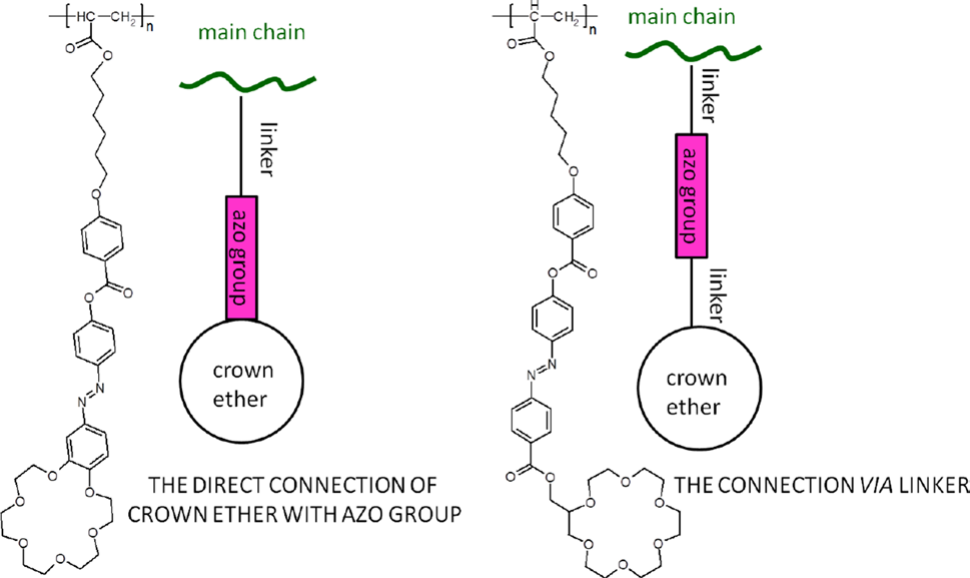
Photochromic crown ethers-containing LC polymers and their schematic representation [260]

The attaching of azobenzene groups to side-chains of liquid crystalline polymers results in light-controllable polymer materials. Such films and coatings can be applied for example as optical molecular devices. One group of such materials are derivatives of crown ethers bearing residues able to form liquid crystalline (LC) phases. Complex formation by crown ether moiety can lead to the appearance or disruption of supramolecular structures. A series of photochromic azobenzene-crown-containing compounds forming crystalline and nematic phases were described by Shinkai and co-workers [258]. Photochromic crown ether-containing LC homopolymers and copolymers based on azobenzenes were later described also by Bobrovsky and co-workers [259]. The complexation of metal ions by these compounds cause the decrease of clearing temperature and sometimes the transition into the amorphous state. The investigation of the relationship between molecular architecture of this type polymers and their photo-optical properties and phase behavior was the main scope of studies. Bobrovsky and co-workers [260] described among others the synthesis and properties of two types of polymers differing in the position of the crown ether in relation to the photoresponsive azobenzene residues (Fig. 89). Macrocyclic moiety was linked directly to chromogenic residue (Fig. 89, left) or via carboxymethylene spacer (Fig. 89, right). Phase behavior, spectral properties and kinetics of photo-orientation processes inside thin films of polymers shown in Fig. 89 were found to be dependent on the location of crown ether with respect to the residue bearing azo group. In the case where a crown ether was introduced as separated non photochromic side group the decrease of the degree of photoinduced orientational order was found. Complex formation with potassium ions by compound shown in Fig. 89 (left) results in the decrease in degree of the photoinduced order. Possible application in the creation of new sensing materials was suggested.

**Scheme 23:**
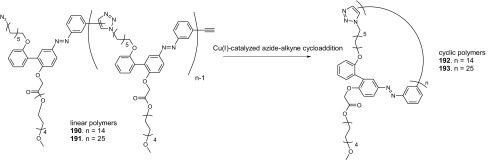
Linear **190** and **191** and cyclic **192** and **193** photoresponsive polymers described by Zhu and co-workers [261]

Zhu and co-workers [261] described linear **190** and **191** and cyclic **192** and **193** (Scheme 23) amphiphilic polymers containing azobenzene moieties. Macrocyclic polymers were obtained in Cu(I)-catalyzed azide-alkyne cycloaddition to achieve intramolecular macroring closure process, one of the most popular and powerful “click” synthetic reaction [262].

According to the obtained results, azomacrocycles exhibit increased glass transition temperatures, faster *trans*–*cis*–*trans* photoisomerization, and enhanced fluorescence intensity in comparison to their acyclic analogues. In water:THF mixture (1:1, v/v) both macrocylic and linear polymers self-assemble into spherical nanoparticles. The size of aggregates formed by cyclic compounds are significantly smaller than those of corresponding linear analogues due to more dense and compact packing of macrocyle-bearing particles. Alternating irradiation of nanospheres with ultraviolet (365 nm) and visible (435 nm) light causes isomerization of the azo group located in polymer main chain. This induces reversible shift of the hydrophilic-hydrophobic balance of macromolecules and leads to the dissociation and reaggregation of the particles. The photoresponsive behavior is slower for nanospheres containing cyclic polymers than in the case of particles with materials of linear structure.

The skeleton of the photoresponsive polymers also can be enriched with other functionalities that for example are able to form complexes with metal cations.

**Fig. 90:**
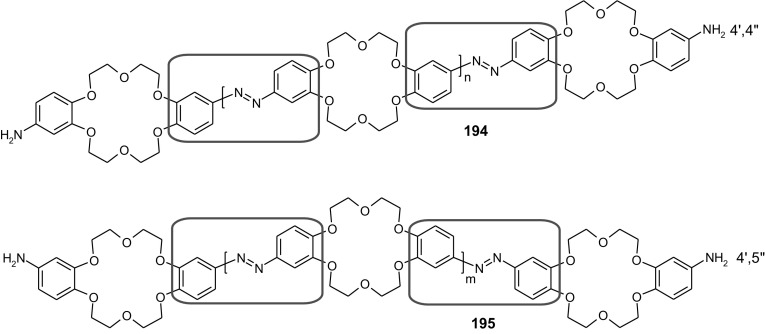
Structure of poly(azodibenzo-18-crown-6-ether)s **194** and **195** [263]

Wiktorowicz et al. [263] prepared polymers comprising dibenzo-18-crown-6 moieties joined by azo bridges **194, 195** using reductive coupling procedure (Fig. 90). Spectrophotometric measurements showed that the polymers are pH-sensitive and exhibit solvatochromic properties. Alternating irradiation of the polymers with UV and visible light induces reversible *trans*–*cis*–*trans* photoisomerization. Due to the presence of crown ether cavity, the described polymers interact with Ba^2+^ ions and also with low molar mass pyridinium type guests, leading to complex-induced phase separation in solvents of lower polarity. In alcohols the polymers reveal thermo-responsive behavior exhibiting the upper critical solution temperature type transitions. This effect depends on the polymer concentration and the degree of polymerization.

**Fig. 91:**
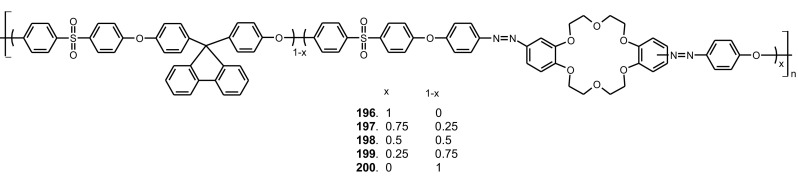
Copoly(ether sulfone)s with azo dibenzo-18-crown-6 and fluorene fragments [264]

Cozan and co-workers [264] described the preparation of copoly(ether sulfone)s with azocrown ether and fluorene fragments. The polymers **196**–**200** (Fig. 91) showed good solubility in solvents of different polarity. Thermogravimetric analysis showed the lowest thermal stability of the copolymer **196** among all investigated polymers as it contains only azo-crown ether units that are sensitive to thermal degradation. The insertion of fluorene moieties into a polymer chain significantly enhances thermal stability. The *trans* to *cis* isomerization of the polymers in DMSO occurs after irradiation with UV light (at 375 nm). The rate constant of the first order photoisomerization increases with decreasing the number of azobenzene units. It was also found that complexation of K^+^ inside the macrocyclic cavity increases *trans* to *cis* isomerization rate.

Photo-induced structural transitions of azo compound bearing dibenzo-24-crown-8 (DB24C8) moiety, dibenzylammonium salt (DBA), and 1,2,3-triazole groups were tested by Dong et al. [265]. Due to host–guest interactions between DB24C8 and DBA from separated molecules linear supramolecular polymers of 1:1 threaded structures (pseudorotaxanes) are formed. The presence of azobenzene moiety allows to control the complex formation, as *trans*-azobenzene-appended DBA interacts with DB24C8 stronger than its *cis* isomer. After addition of [PdCl_2_(PhCN)_2_], 1,2,3-triazole rings of different polymer chains are linked together by the metal coordination, what leads to the formation of cross-linked supramolecular polymers. In dichloromethane the cross-linked assemblies have a form of red gel. UV irradiation (365 nm) of linear and branched polymers induces *trans* to *cis* isomerization, resulting in weaker host–guest interactions and, in a consequence, dissociation of supramolecular polymers. The structural change of cross-linked supramolecular polymer is manifested by naked eye observable decrease of viscosity. The reformation to the gel state is achieved by exposing the solution to visible light (430 nm).

**Scheme 24:**
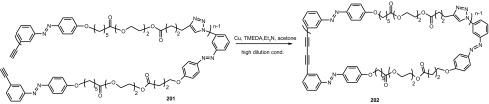
The synthesis of the molecularly-defined linear **201** and cyclic oligomers **202** (n = 1–6) [266]

The photoisomerization of a series of macrocyclic oligomers containing azobenzene moiety in the main chain and their linear analogs was studied by Zhu and co-workers [266] (Scheme 24). Tetraethylene glycol (TEG) was chosen as the building block for the preparation of amphiphilic polymers of good solubility. According to the UV–Vis spectrophotometry it was shown that the *trans* to *cis* and reverse process are the first order reactions for both linear **201** and cyclic **202** compounds. The estimated values of rate constants for macrocyclic oligomers are distinctly higher (for *trans* to *cis* isomerization) and slightly higher (for *cis* to *trans* isomerization) in comparison with results for linear ones, especially for *n* = 1. This can be explained by the more stable conformation of cyclic *cis*-azobenzene than linear *trans* analog.

**Scheme 25:**
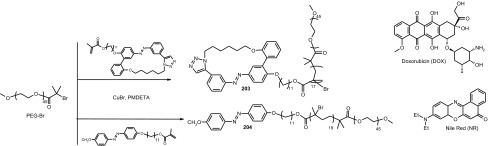
Poly(ethylene glycol)methyl ether based amphiphilic copolymers with cyclic azobenzene unit **203** and its linear analog **204** [267]. The chemical formulas of doxorubicin and Nile Red are also shown

In turn, poly(ethylene glycol)methyl ether was used as building block for other amphiphilic copolymers with cyclic azobenzene unit **203** [267]. For comparative purposes a linear analog **204** was also obtained. The synthetic route is shown in Scheme 25. The obtained copolymers assemble in phosphate buffer solution (pH 7.4) into stable vesicles with hydrophobic blocks containing the azobenzene moieties aggregated in the membranes of the vesicles, and the hydrophilic PEG arrangements on the outer and inner surface of the vesicles. Due to presence of azo moiety the obtained polymers are not only photoresponsive, but also sensitive towards reducing reagents. These properties were used for the investigation of the encapsulation and release of Nile Red (NR- a model compound for drug delivery system) and anticancer drug doxorubicin (DOX). NR-loaded vesicles are fluorescent. The intensity of fluorescence can be controlled by illumination with UV light (365 nm). The reverse *cis*–*trans* process occurs upon irradiation with visible light at 435 nm.

Azo compounds can be reduced by azoreductase or popular reducing reagents, such as for example sodium dithionite. The result is azo bond cleavage, which can be used in effective drug transport. DOX-loaded vesicles were investigated in reductant-release of the encapsulated substance. The release rate of DOX from cyclic polymer **203** is higher compared with the linear analog **204**. This points out the importance of investigated copolymers—particularly cyclic compounds—as potential agents in the treatment of colon disease.

**Fig. 92:**
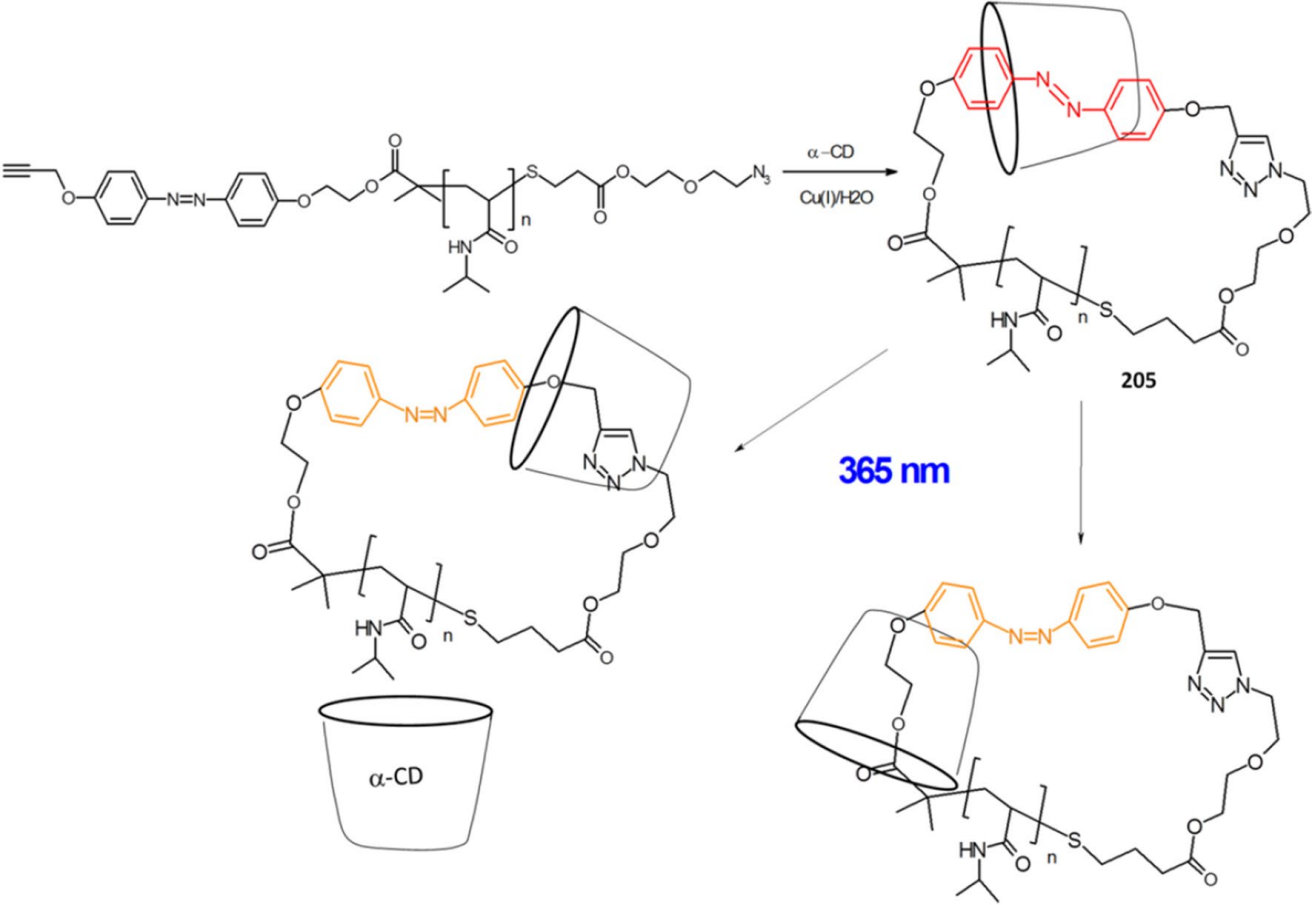
Cyclic azo-poly(*N*-isopropylacrylamide) with interlocked α-cyclodextrin **205** and photoinduced molecular motion within the polymer [268]

Combination of cyclodextrin and azobenzene units bearing polymer were used by Winnik and co-workers [268] to obtain molecular “charm bracelets”. The cyclic poly(*N*-isopropylacrylamide) with azobenzene inserted in the main chain **205** (Fig. 92) was synthesized by the “click” ring closure of α-azobenzene ω-azido poly(*N*-isopropylacrylamide) in the form of inclusion complex with α-cyclodextrin. UV-light irradiation of aqueous solution of **205** (at 365 nm) induces motion within the molecule as *cis*-azobenzene unit obtained upon photoisomerization, due to its size, is expelled from the α-cyclodextrin cavity pushing the host to the other sections of the polymeric ring. The *trans* to *cis* photoisomerization does not affect the temperature of phase transition of the polymer, whereas in the case of the analog without cyclodextrin the temperature increases by 1.7 °C. This may be explained assuming that the enhanced polarity due to *trans* to *cis* isomerization in polymer **205** is overshadowed by the strong hydrophilicity of α-cyclodextrin moiety interlocked along the polymer ring and no change of the phase transition temperature is observed.

## Miscellaneous macrocyclic systems bearing azo group(s)

**Fig. 93:**
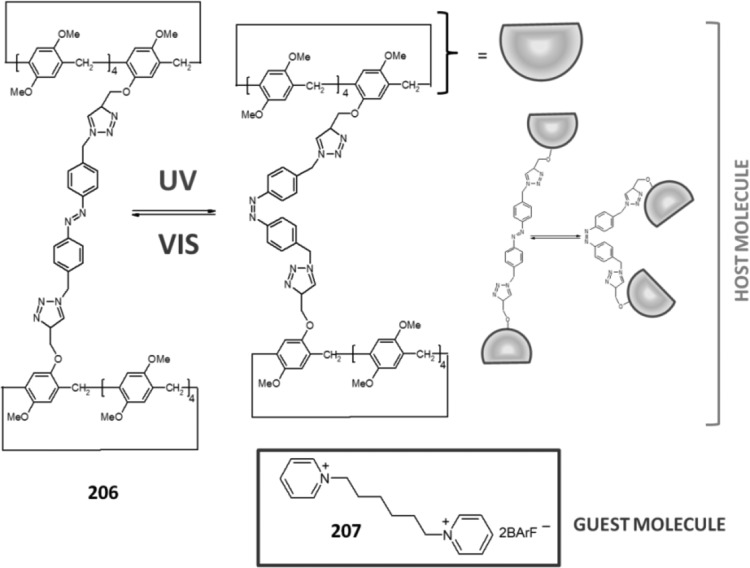
Chemical structures of the azobenzene-bridged pillar[5]arene dimer **206** and the homoditopic pyridinium guest molecule **207** [269]

Molecular containers such as pillarenes are promising blocks for building of photoswichable assemblies. Ogoshi et al. [269] obtained supramolecular polymers consisting of *trans*-azobenzene-bridged pillar[5]arene dimer **206** and bispyridinium cations linked by hexamethylene unit **207** (Fig. 93). On the basis of ^1^H NMR spectroscopy it was stated that in dichloromethane at low concentration (2 mM) complexes of 1:1 stoichiometry are formed, in which pyridinium cation moiety **207** is included inside the cavity of **206**. At higher concentration (100 mM) supramolecular assemblies were detected according to DOSY ^1^H NMR experiments. Irradiation of diluted solution with UV light induces *trans* to *cis* isomerization of component **206**. At the photostationary state the ratio of *trans* to *cis* isomer is 26:74. At high concentration nearly half of *trans*-**206** does not convert into the *cis* form. Efficient reverse process occurs after exposure to visible light (436 nm). Under equilibrium the ratio of *trans* to *cis* isomer is 93:7. It was demonstrated that photoisomerization from *trans* to *cis* form weakens the host–guest interactions, probably due to the steric hindrance caused by the *cis* isomer of **206**. As a consequence, the created at high concentration, supramolecular polymers disassembly after UV-light irradiation. Photo-switching between assembly and disassembly of supramolecular system looks completely reversible by alternating irradiation between visible and UV light.

**Scheme 26:**
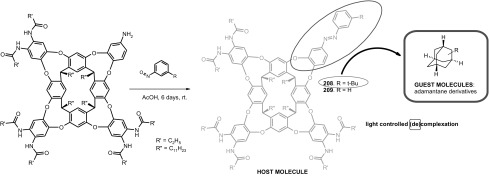
Synthesis of azo derivatives of cavitands **208, 209** and their complexation properties [270]

Cavitands **208** and **209** (Scheme 26) bearing azo moiety integrated with macrocyclic [270] structure undergo *trans*–*cis* photoisomerization upon illumination with UV light (365 nm). *Cis*–*trans* conversion proceeds by heating to 164 °C for 5 min or irradiating with 450 nm light for 20 min. *Trans*–*cis* and *cis*–*trans* cycles can be repeated 5 times without degradation of the system. Both the *trans* isomers of **208** and **209** have deep cavities able to bind guest molecules. In fact, **208** and **209** were found to form complexes with small molecules of adamantane series in d_12_-mesitylene. The highest values of stability constants were found for 1-adamantanecarbonitrile and 2-adamantanone. It was explained assuming the possibility of stabilization of formed complexes by hydrogen bonding and polar interactions with the upper rim of the cavitands. The complexation of adamantane guests can be light controlled, namely irradiation controls uptake and release of guest for **208**.

**Fig. 94:**
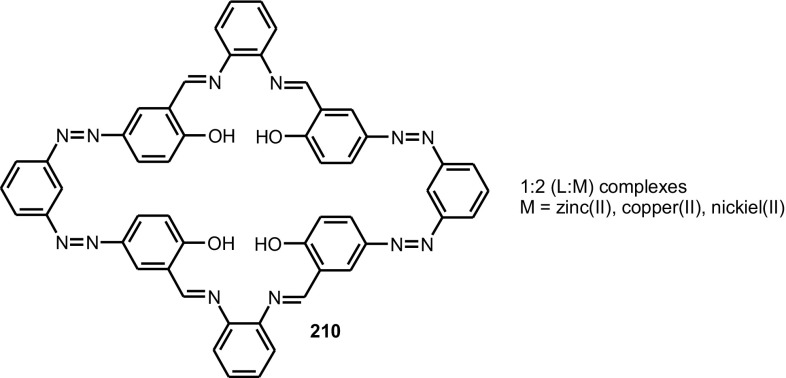
Macrocyclic Schiff base **210** bearing inherent azo groups [271]

Azo moieties can constitute part of macrocyclic Schiff bases, as for example fluorescent product of [2 + 2] condensation of *N*,*N*′-bis-(2-hydroxybenzaldehyde-5-yl)-benzene-1,3-diazene and benzene-1,2-diamine **210** (Fig. 94) [271]. The stoichiometry of complexes of **210** with zinc(II), copper(II) and nickel(II) is 1:2 (L:M) as showed by elemental analyses and spectral studies. Fluorescence spectra registered in DMSO showed quenching of fluorescence of Schiff base upon metal binding.

**Fig. 95:**
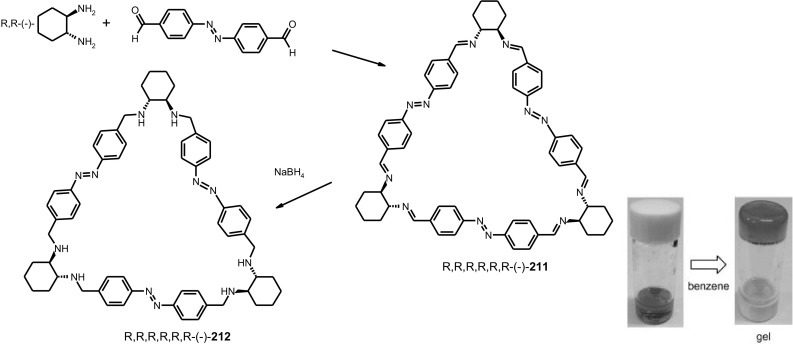
Left: the synthesis of chiral macrocyclic Schiff base **211** and its reduced derivative **212**. Right: sol–gel transformation of **211**. Reprinted from [272]. Copyright 2010 with permission from Elsevier

Another example of azo derivative of Schiff base type can be chiral macrocycle **211** (Fig. 95), with three azobenzene residues [272]. This compound was obtained by [3 + 3] condensation reaction of enantiomerically pure *trans* 1,2-diaminocyclohexane with azobenzene-4,4′-dicarbaldehyde in dichloromethane. The subsequent sodium borohydride reduction of **211** produces macrocyclic hexaamine **212**, also with three azobenzene units. Irradiation of chloroform solution of (*R*,*R*,*R*,*R*,*R*,*R*)-**211** with 365 nm light for 30 min, causes the decrease of absorption peak at 348 nm and increase of bands intensity at 273 and 450 nm. The back process occurs upon leaving the solution at room temperature for 48 h. Similar observations were made for reduced analog (*R*,*R*,*R*,*R*,*R*,*R*)-**212**. Interesting properties were found for **211** dissolved in benzene. In this solvent a translucent and orange colored gel was obtained. Scanning electron microscopy (SEM) measurements of the obtained material showed the presence of elongated fibers with diameters of around 1 μm in the dried gel. The illumination of the gel with UV light for several hours led to gel–sol transformation. The reverse process occurs upon heating the sol. Macrocycle **212** forms inclusion complexes with various aromatic organic guest molecules. Complexes of 1:1 stoichiometry were found for benzene and toluene as the guests and 2:1 (**212**:guest) when *o*-, *m*-, and *p*-xylenes were complexed. This can indicate better complementarity of the host and benzene or toluene than in the case of larger xylene molecules.

**Fig. 96:**
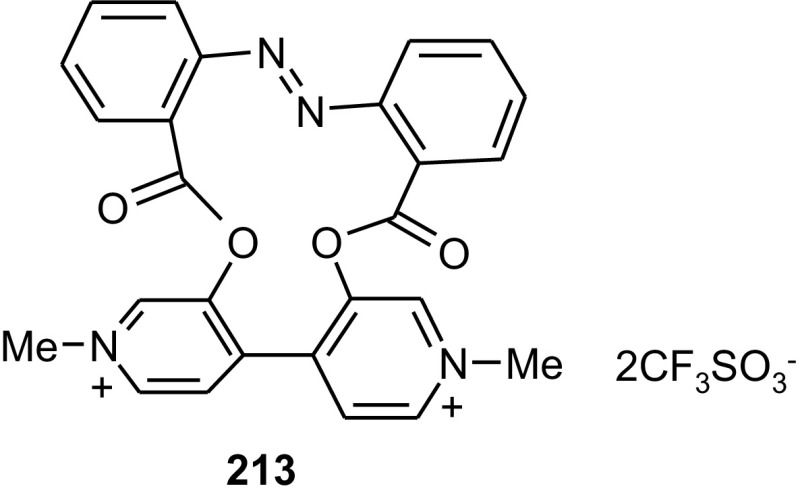
Highly-strained cyclophane **213** comprising azobenzene and methyl viologen units [273]

Not always photoinduced transformations are reversible. Red colored compound **213** (Fig. 96) obtained in reaction of 3,3′-dihydroxy-4,4′-bipyridine and azobenzene-2,2′-dicarboxylic acid in dichloromethane is a highly-strained cyclophane **213** comprising azobenzene and methyl viologen units [273]. Cyclic voltammperometric measurements showed its unique irreversible electrochemical behavior. *Trans–cis* isomerisation upon visible light illumination of **213** is also irreversible.

**Fig. 97:**
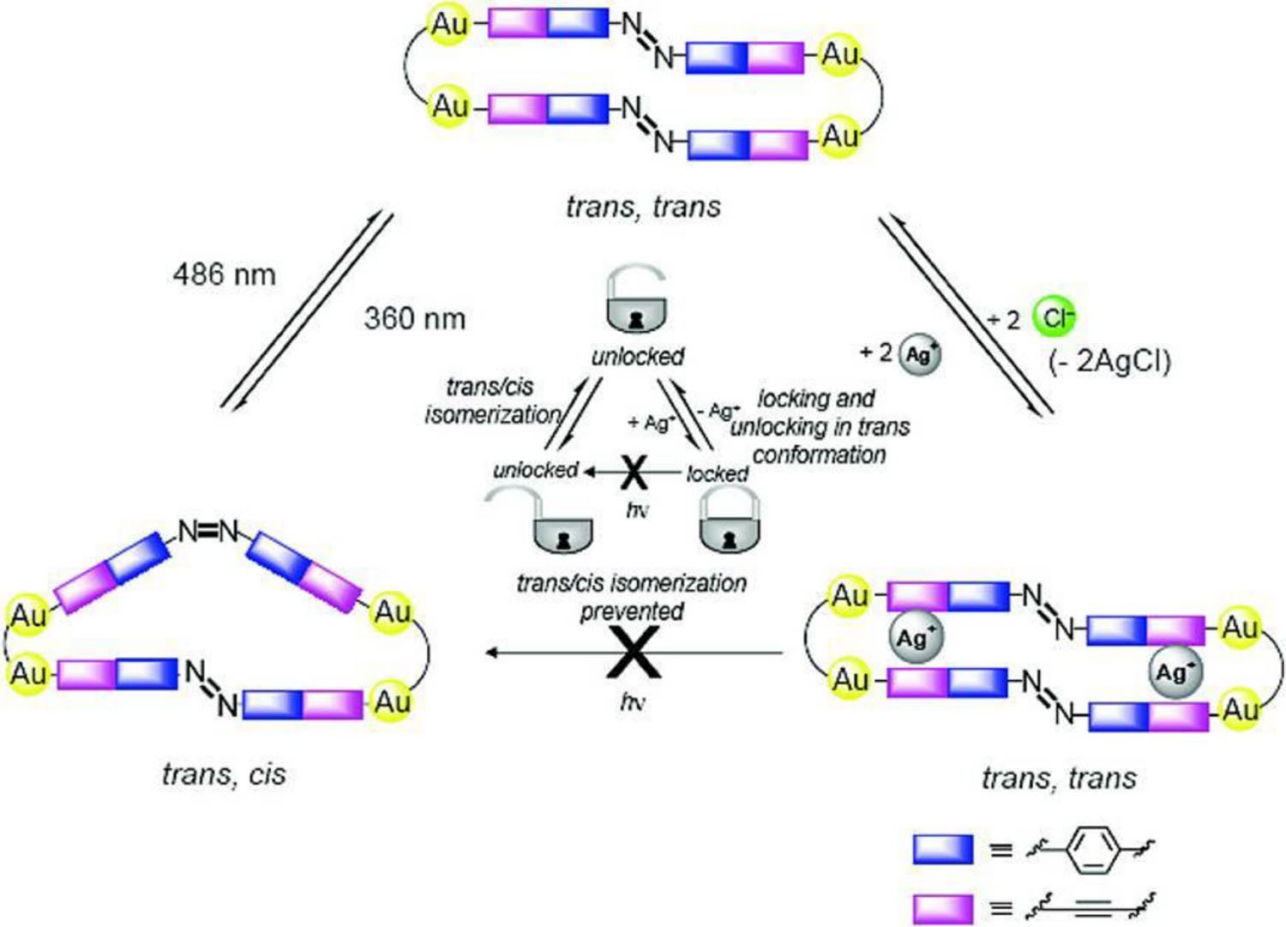
Schematic diagram demonstrating the “locking” and “unlocking” mechanism brought about by the addition and removal of Ag^+^ ions in preventing and facilitating *trans*–*cis* isomerization of [Au_4_(P^P)_2_(C≡C–L–C≡C)_2_]. Reprinted with permission from [274]. Copyright 2007 American Chemical Society

The photo- and redox properties of azo compounds can be extended to more sophisticated systems due to incorporation of transition metal cations into their structure. In such cases both the photoisomerization of azo compounds and chemical and physical properties of transition metal cations (optical, redox, magnetic etc.) can be utilized for construction of functional systems. Among the others, tetranuclear macrocyclic gold(I) alkynyl phosphine complexes with two azobenzene moieties, were obtained (shown schematically in Fig. 97) and investigated as photoswichable system [274]. It was found that the photo switching of gold(I) complex could be locked or unlocked with a second input: by the addition or removal of silver(I) ions.

**Fig. 98:**
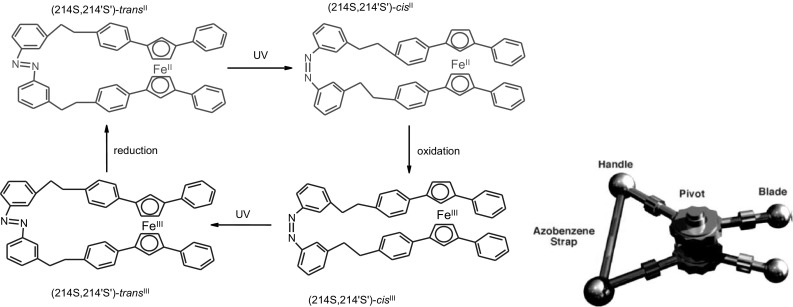
Left: the operation of molecular pliers **214** by light and redox stimuli. Right: schematic illustration of molecular pliers. Reprinted from [275]. Copyright 2008 with permission from Royal Society of Chemistry

The conformational change of the molecule which is a consequence of the reversible *trans*–*cis* isomerization and the red-ox properties of iron are good examples of construction block for multi-stimuli molecular devices. The interlocking of a ferrocene-based rotary module with a photochromic azo unit of molecular machines operating *via* power-conversion mechanisms can be constructed. Such systems resemble daily used devices such as pliers **214** shown in Fig. 98 [275].

**Fig. 99:**
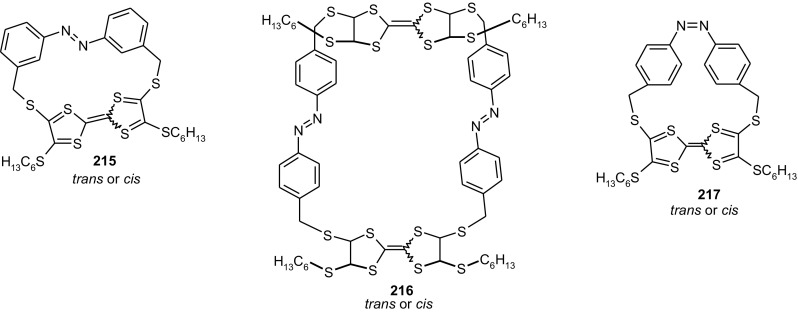
Macrocyclic azocompounds bearing tetrathiafulvalene units **215**–**217** [276]

Azov et al. [276] investigated macrocyclization of tetrathiafulvalene dithiolates with bis-bromomethylazobenzenes (Ab) under high dilution conditions (Fig. 99). The reaction afforded [1 + 1] cyclization product **215** with *m*-Ab and [2 + 2] cyclization product **216** with *p*-Ab in good yields (above 66%). Irradiation of *p*-Ab with UV light (365 nm, 0 °C) before reaction results in obtainment of *cis*-azobenzene bearing product **217** (1 + 1 cyclization type). Analysis of cyclic voltammograms registered in dichloromethane/0.1 M Bu_4_NClO_4_ showed that the electrochemical properties of tetrathiafulvalene moiety strongly depend on configuration (*trans* or *cis*) of azobenzene unit.

**Fig. 100:**
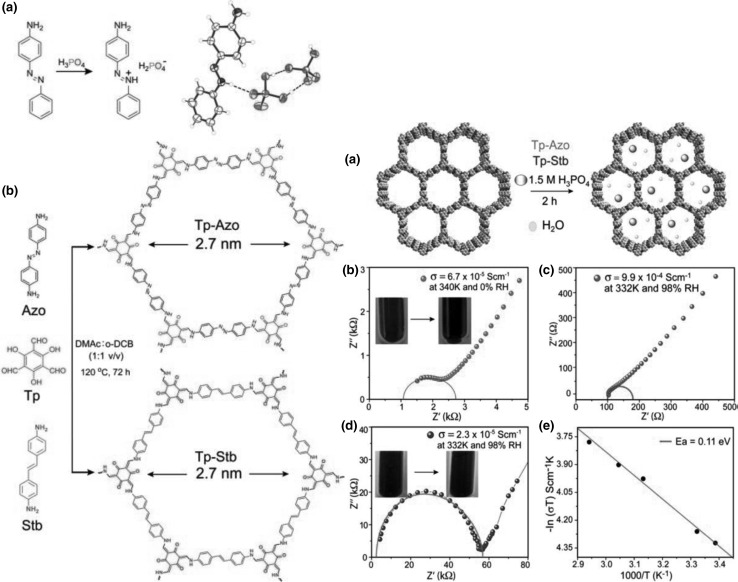
Left: **a** crystal structure of 4-[(*E*)-phenyl-diazenyl]anilinium dihydrogen phosphate. **b** schematic of Tp-Azo and Tp-Stb synthesis. Right: **a** schematic of H_3_PO_4_ doping of COFs. Proton conductivity of PA@Tp-Azo in **b** anhydrous and **c** wet conditions. **d** Proton conductivity of PA@Tp-Stb in wet conditions. **e** Arrhenius plot for PA@Tp-Azo in hydrous conditions. Reprinted with permission from [277]. Copyright 2014 American Chemical Society

Banerjee and co-workers [277] synthesized and compared properties of two covalent organic frameworks (COFs) (Fig. 100) being derivatives of triformylphloroglucinol and 4,4′-azodianiline (**Tp-Azo**) or 4,4′-diaminostilbene (**Tp-Stb**). Azo-functionalized COF **Tp-Azo** exhibits better stability, porosity and crystallinity than stilbene-bearing analogoue **Tp-Stb**. The analysis of N_2_ absorption isotherm of **Tp-Azo** treated with 9 M HCl indicates the retention of intrinsic porosity of the azo-functionalized COF, whereas in the case of **Tp-Stb** decrease of porosity after the acid treatment was observed. According to TGA experiments it was stated that **Tp-Azo** possesses higher acid loading (5.4 wt%) than **Tp-Stb** (2.8 wt%). Doping of H_3_PO_4_ to the azo-functionalized COF leads to immobilization of the acid inside the framework pores, what enables proton transfer in both the anhydrous (σ = 6.7 × 10^−5^ S cm^−1^ at 340 K) and hydrated state (σ = 9.9 × 10^−4^ S cm^−1^ at 332 K under 98% relative humidity). Stilbene-bearing COF shows almost zero proton conductivity in anhydrous milieu and a poor proton conductivity value (σ = 2.3 × 10^−5^ S cm^−1^) at 332 K under 98% relative humidity.

**Fig. 101:**
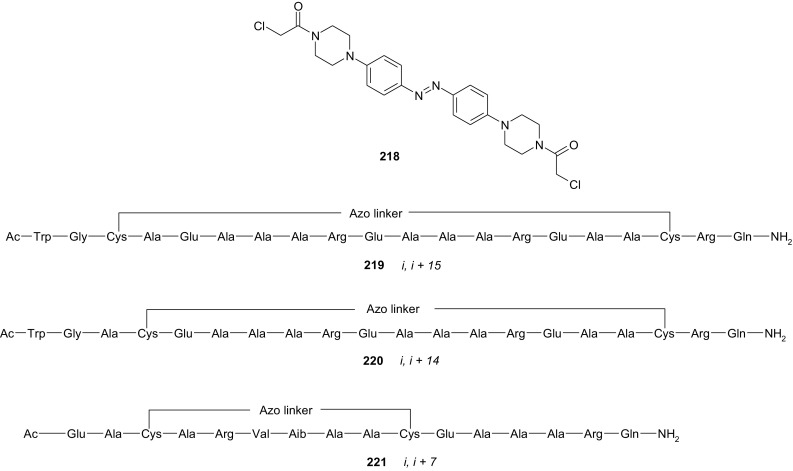
Azobenzene cross linker **218** and primary sequence of the cross linked peptides **219–221** described by Woolley and co-workers [282]

Azobenzene isomerization has been also utilized to drive functional changes in biomolecules such as: peptides, proteins, lipids, nucleic acids and carbohydrates. Comprehensive review of such applications can be found in the work of Beharry and Woolley from 2011 [72]. To apply azobenzene to direct protein conformational change in biological systems several requirements need to be fulfilled such as: (i) substantial structural change of azo bearing unit upon isomerization that can be coupled to protein conformational change, (ii) stability of the azo unit in a cellular environment, (iii) a suitable for cells and tissues irradiation wavelength and rate of thermal relaxation. Photocontrol of cyclic peptides was investigated among the others by Schutt et al. [278]. The authors described cyclization of a heptapeptide containing the Arg-Gly-Asp (RGD) sequence with 4-aminomethylphenylazobenzoic acid (AMPB). Studies of the cyclic peptide affinity to the cell surface receptor αVβ3 integrin show that RGD binds target protein stronger when azo unit is in its *trans* form. The cell adhesion can be also controlled by tethering of RGD peptide to a surface via azobenzene linker. 3-((4′-aminomethyl)phenylazo)benzoic acid was used to control the conformation of a cyclic peptide based on nNOS β-finger [279]. The *trans* isomer shows binding affinity towards target protein—α-1-syntrophin. Irradiation of the system with light at 330 nm enables the protein recognition. According to FTIR and NMR experiments, the isomerization azobenzene unit induces the formation of secondary, antiparallel β-type structure of the peptide ensuring the efficient interactions with α-1-syntrophin. Incorporation of azobenzene unit into protein disulfide isomerase *via* bis-cysteinyl active site was used to the obtainment of a simple model for allosteric conformational rearrangements [280]. It was stated, that the geometric changes accompanying isomerization of the azo group induce a rearrangement of peptide sequence changing energy landscape of the peptide and both isomers *trans* and *cis* exist in defined conformational states stabilized by disulfide bridge. Derda et al. [281] proposed bis(allenamide) functionalized azobenzene reagents for conversion of cysteine containing peptides to light responsive macrocycles. In comparison with typically used bis-alkyl halides containing azobenzene the allenyl amide derivatives ensure 2–3 order of magnitude faster macrocyclization by cysteine ligation in model peptide and those displayed on M13 phage. Woolley and co-workers [282] incorporated a thiol reactive azobenzene cross linker **218** into peptide backbone receiving cyclic azopeptides **219–221** (Fig. 101). Upon irradiation of the peptides in aqueous solution with blue light at 400–450 nm *trans* to *cis* isomerization occurs. Obtained *cis* isomers relax thermally with a half-life of about 1 s. It was stated, that azobenene linker **218** can be used to control of helical content of attached peptide, as in its *trans* form the linker bridges Cys residues spaced *i, i* + *15* (peptide **219**) in an α-helix. Switching **218** to its *cis* isomer causes the decrease of the helix content of **219** and the increase of the helix content of **221**. After photoisomerization no helix content of **220** is detected.

Jaeschke and co-workers [283] described carbohydrate-based macrocycles obtained from isothiocyanate-armed bis-azobenzene glycosides and piperazine. Isomerization of glycoazobenzene precursor molecules before the reaction ensured more efficient macrocyclization (yields: 48–65%). Obtained *trans* macrocycles isomerize into their *cis* forms upon UV-light irradiation what results in tremendous change of chirality with a strong helical induction in the *cis* state. The isomerization process is fully reversible by thermal relaxation, whereas upon irradiation with blue light only partially recovery of *trans* isomer is obtained.

## Summary

The above review article is a subjective point of view on the current state of art in the synthesis and properties of selected azomacrocyclic compounds. It covers mainly the last 10 years, however, many of the previous works were also cited, to give more comprehensive background of the subject. Our intention was to underline the importance of very simple, seemingly tiny, functional –N=N– group, which can be incorporated into almost any molecule (material) giving extraordinary properties, especially when macrocyclic compounds are regarded. The presence of macrocyclic scaffold can have an enormous influence on switching properties of azo group due to ring strain and substituent effects. Photochemical characteristic of cyclic azobenzenes depends also on other factors, such as the number of azo units in the macrocycle, the symmetry of total molecule and the degree of conjugation, what makes the design of azomacrocyclic compounds a challenging task. Reversible *trans–cis* isomerization gives an opportunity to control the macrocycles structures at the molecular level what can be utilized for instance in the development of light-induced assembly/disassembly processes of supramolecular systems or in morphological transformation of assemblies. Binding properties of macrocyclic hosts e.g. crown ethers or cyclodextrins can be regulated by photoswitching of azo moiety, what finds applications among the others in ion transport through membranes and controlled drug release systems. Chromogenic and electroactive properties of azo group enable effective macrocycle use in optical and electrochemical sensors development. In the above manuscript we wanted to signalize the multifarious areas of science, technology and medicine where macrocyclic azo compounds can find applications. We believe the review will be helpful for readers interested in organic, analytical and practical aspects of supramolecular chemistry.

## References

[1] Izatt RM (2017). Charles J. Pedersen’s legacy to chemistry. Chem. Soc. Rev.

[2] Pedersen CJ (1967). Cyclic polyethers and their complexes with metal salts. J. Am. Chem. Soc..

[3] Pedersen CJ (1967). Cyclic polyethers and their complexes with metal salts. J. Am. Chem. Soc..

[4] Hyun MH (2016). Liquid chromatographic enantioseparations on crown ether-based chiral stationary phases. J. Chromatogr. A.

[5] Scriba GKE (2016). Chiral recognition in separation science—an update. J. Chromatogr. A.

[6] Kakhki RM (2013). Recent developments in microextraction techniques based on crown ethers. J. Incl. Phenom. Macrocyc. Chem.

[7] Della Sala G, Sicignano M, Schettini R, De Riccardis F, Cavallo L, Minenkov Y, Batisse C, Hanquet G, Leroux F, Izzo I (2017). Switchable diastereoselectivity in the fluoride-promoted vinylogous Mukaiyama-Michael reaction of 2-[(trimethylsilyl)oxy]furan catalyzed by crown ethers. J. Org. Chem.

[8] Liang Y-R, Wu Q, Lin X-F (2017). Effect of additives on the selectivity and reactivity of enzymes. Chem. Rec.

[9] Bako P, Keglevich G, Rapi Z (2010). Asymmetric phase transfer reactions catalyzed by chiral crown ethers derived from monosaccharides. Lett. Org. Chem.

[10] Li J, Yim D, Jang WD, Yoon J (2017). Recent progress in the design and applications of fluorescence probes containing crown ethers. Chem. Soc. Rev.

[11] Dietrich B, Lehn J-M, Sauvage J-P (1969). Diaza-polyoxa-macrocycles et macrobicycles. Tetrahedron Lett.

[12] Dietrich B, Lehn J-M, Sauvage J-P (1969). Les cryptates. Tetrahedron Lett.

[13] Cram DJ, Kaneda T, Lein GM, Helgeson RC (1979). Spherand containing an enforced cavity that selectively binds lithium and sodium-ions. J. Am. Chem. Soc..

[14] Cram DJ, Dicker IB, Knobler CB, Trueblood KN (1982). Spherand hosts containing cyclic urea units. J. Am. Chem. Soc..

[15] Lein GM, Cram DJ (1982). Spherand complexation and decomplexation rates with sodium and lithium picrates, and activation parameters for decomplexation. J. Chem. Soc. Chem. Commun.

[16] Cram DJ, Lein GM (1985). Host guest complexation. 36. Spherand and lithium and sodium-ion complexation rates and equilibria. J. Am. Chem. Soc..

[17] Cram DJ, Cram JM (1974). Host-guest chemistry. Science.

[18] Cram DJ (1988). The design of molecular hosts, guests, and their complexes (Nobel lecture). Angew. Chem. Int. Ed..

[19] Lehn J-M (1985). From molecular to supramolecular chemistry—science, art and industry. Int. Sci. Rev.

[20] Lehn J-M (1988). Supramolecular chemistry—scope and perspectives molecules, supermolecules, and molecular devices (Nobel lecture). Angew. Chem. Int. Ed..

[21] Lehn J-M (1990). Perspectives in supramolecular chemistry - from molecular recognition towards molecular information-processing and self-organization. Angew. Chem. Int. Ed.

[22] Lehn J-M (1993). Supramolecular chemistry. Science.

[23] Pedersen CJ (1988). The discovery of crown ethers (Noble lecture). Angew. Chem. Int. Ed..

[24] https://www.nobelprize.org/nobel_prizes/chemistry/laureates/. Accessed: 23 Aug 2017

[25] Astumian RD (2017). How molecular motors work—insights from the molecular machinist’s toolbox: the Nobel prize in Chemistry 2016. Chem. Sci.

[26] Burrows H, Weir R, Stohner J (2016). 2016 Nobel prize in chemistry. Pure. Appl. Chem.

[27] Simmons HE, Park CH (1968). Macrobicyclic amines. I. Out-in isomerism of 1,(k + 2)-diazabicyclo[k.l.m]alkanes. J. Am. Chem. Soc..

[28] Simmons HE, Park CH (1968). Macrobicyclic amines. II. out-out in-in prototropy in 1,(k + 2)-diazabicyclo[k.l.m] alkaneammonium ions. J. Am. Chem. Soc..

[29] Simmons HE, Park CH (1968). Macrobicyclic amines. III. Encapsulation of halide ions by in,in-1,(k + 2)-diazabicyclo[k.l.m.]alkane ammonium ions. J. Am. Chem. Soc..

[30] Lim JYC, Marques I, Thompson AL, Christensen KE, Felix V, Beer PD (2017). Chalcogen bonding macrocycles and [2]rotaxanes for anion recognition. J. Am. Chem. Soc..

[31] Kaabel S, Adamson J, Topic F, Kiesila A, Kalenius E, Oeren M, Reimund M, Prigorchenko E, Lookene A, Reich H, Rissanen K, Aav R (2017). Chiral hemicucurbit[8]uril as an anion receptor: selectivity to size, shape and charge distribution. Chem. Sci.

[32] Gale PA, Howe ENW, Wu X (2016). Anion receptor chemistry. Chem.

[33] Langton MJ, Serpell ChJ, Beer PD (2016). Anion recognition in water: recent advances from a supramolecular and macromolecular perspective. Angew. Chem. Int. Ed.

[34] Liu Z, Nalluri SKM, Stoddart JF (2017). Surveying macrocyclic chemistry: from flexible crown ethers to rigid cyclophanes. Chem. Soc. Rev.

[35] Kolesnichenko IV, Anslyn EV (2017). Practical applications of supramolecular chemistry. Chem. Soc. Rev.

[36] Wong JK-H, Todd MH, Rutledge PJ (2017). Recent advances in macrocyclic fluorescent probes for ion sensing. Molecules.

[37] Rodrigo F, Gamez F, Aviles-Moreno JR, Pedrosa JM, Martinez-Haya B (2016). Enhanced cation recognition by a macrocyclic ionophore at the air-solution interface probed by mass spectrometry. Phys. Chem. Chem. Phys.

[38] Kataev EA, Backmann N, Shumilova TA, Rueffer I, Lang H (2016). Calix[4]pyrroles bearing quinolinium moiety for halide sensing in aqueous solution. Supramol. Chem.

[39] You L, Zha D, Anslyn EV (2015). Recent advances in supramolecular analytical chemistry using optical sensing. Chem. Rev.

[40] Bistri O, Reinaud O (2015). Supramolecular control of transition metal complexes in water by a hydrophobic cavity: a bio-inspired strategy. Org. Biomol. Chem.

[41] Anslyn EV (2007). Supramolecular analytical chemistry. J. Org. Chem.

[42] Sathiyajith C, Shaikh RR, Han Q, Zhang Y, Meguellati K, Yang YW (2017). Biological and related applications of pillar[n]arenes. Chem. Commun.

[43] Naseer MM, Ahmed M, Hameed S (2017). Functionalized calix[4]arenes as potential therapeutic agents. Chem. Biol. Drug Des.

[44] Dong YH, Cao LP (2016). Functionalization of cucurbit[n]uril. Prog. Chem.

[45] Bey A, Dreyer O, Abetz V (2017). Thermodynamic analysis of alkali metal complex formation of polymer-bonded crown ether. Phys. Chem. Chem. Phys..

[46] Toeri J, Osorio-Madrazo A, Laborie MP (2017). Preparation and chemical/microstructural characterization of azacrown ether-crosslinked chitosan films. Materials.

[47] Sawada J, Aoki D, Kuzume M, Nakazono K, Otsuka H, Takata T (2017). A vinylic rotaxane cross-linker for toughened network polymers from the radical polymerization of vinyl monomers. Polym. Chem.

[48] Rodell ChB, Mealy JE, Burdick JA (2015). Supramolecular guest-host interactions for the preparation of biomedical materials. Bioconjug. Chem.

[49] Zerkoune L, Angelova A, Lesieur S (2014). Nano-assemblies of modified cyclodextrins and their complexes with guest molecules: incorporation in nanostructured membranes and amphiphile nanoarchitectonics design. Nanomaterials.

[50] An Q, Dong C, Zhu W, Tao CA, Yang H, Wang Y, Li G (2012). Cucurbit[8]uril as building block for facile fabrication of well-defined organic crystalline nano-objects with multiple morphologies and compositions. Small.

[51] Alexandratos SD, Stine CL (2004). Synthesis of ion-selective polymer-supported crown ethers: a review. React. Funct. Polym.

[52] van Leeuwen T, Gan J, Kistemaker JCM, Pizzolato SF, Chang M-C, Feringa BL (2016). Enantiopure functional molecular motors obtained by a switchable chiral-resolution process. Chem. Eur. J.

[53] Sun J, Wu Y, Wang Y, Liu Z, Cheng Ch, Hartlieb KJ, Wasilewski MR, Stoddart JF (2015). An electrochromic tristable molecular switch. J. Am. Chem. Soc..

[54] Niess F, Duplan V, Sauvage J-P (2014). Molecular muscles: from species in solution to materials and devices. Chem. Lett.

[55] Witus LS, Hartlieb KJ, Wang Y, Prokofjevs A, Frasconi M, Barnes JC, Dale EJ, Fahrenbach AC, Stoddart JF (2014). Relative contractile motion of the rings in a switchable palindromic [3]rotaxane in aqueous solution driven by radical-pairing interactions. Org. Biomol. Chem.

[56] Qu DH, Feringa BL (2010). Controlling molecular rotary motion with a self-complexing lock. Angew. Chem. Int. Ed.

[57] Durot S, Reviriego F, Sauvage J-P (2010). Copper-complexed catenanes and rotaxanes in motion: 15 years of molecular machines. Dalton Trans.

[58] Zollinger H (2003). Color Chemistry. Syntheses, Properties and Applications of Organic Dyes and Pigments.

[59] Esguerra KVN, Lumb J-P (2017). Synthesis of ortho-azophenols by formal dehydrogenative coupling of phenols and hydrazines or hydrazides. Chem. Eur. J.

[60] Fu XP, Wei ZJ, Xia CC, Shen C, Xu J, Yang Y, Wang K, Zhang PF (2017). Palladium-catalyzed direct ortho C-O bond construction of azobenzenes with iodobenzene diacetate via C-H activation. Catal. Lett.

[61] Androvic L, Bartacek J, Sedlak M (2016). Recent advances in the synthesis and applications of azo initiators. Res. Chem. Intermed.

[62] Léonard E, Mangin F, Villette C, Billamboz M, Len C (2016). Azobenzene and catalysis. Catal. Sci. Technol.

[63] Combita D, Concepcion P, Corma A (2014). Gold catalysts for the synthesis of aromatic azocompounds from nitroaromatics in one step. J. Catal.

[64] Qin CG, Li Y, Li HL, Li DW, Niu WW, Shang XY, Xu CL (2013). Novel progresses in synthesis strategies of aromatic azo derivatives. Chin. J. Org. Chem.

[65] Merino E (2011). Synthesis of azobenzenes: the coloured pieces of molecular materials. Chem. Soc. Rev.

[66] Hamon F, Djedaini-Pilard F, Barbot F, Len Ch (2009). Azobenzenes-synthesis and carbohydrate applications. Tetrahedron.

[67] Wu S, Wang J, Song P, Xia L (2017). New insight into the synthesis of aromatic azo compounds assisted by surface plasmon resonance. Plasmonics.

[68] Khaligh NG (2017). Telescopic synthesis of azo compounds via stable arenediazonium bis(trifluoromethane)sulfonimide salts by using tert-butyl nitrite. Dyes Pigments.

[69] Hofmann D, Gans E, Kruell J, Heinrich MR (2017). Sustainable synthesis of balsalazide and sulfasalazine based on diazotization with low concentrations of nitrogen dioxide in air. Chem. Eur. J.

[70] Merino E, Ribagorda M (2012). Control over molecular motion using the cis–trans photoisomerization of the azo group. Beilstein J. Org. Chem.

[71] Hartley GS (1937). The cis-form of azobenzene. Nature.

[72] Beharry AA, Woolley GA (2011). Azobenzene photoswitches for biomolecules. Chem. Soc. Rev.

[73] Beharry AA, Sadowski O, Woolley GA (2011). Azobenzene photoswitching without ultraviolet light. J. Am. Chem. Soc..

[74] Beharry AA, Wong L, Tropepe V, Woolley GA (2011). Fluorescence imaging of azobenzene photoswitching in vivo. Angew. Chem. Int. Ed.

[75] Henkenfeld J, Drzaic P, Yeo JS, Koch T (2011). Review paper: a critical review of the present and future prospects for electronic paper. J. Soc. Inf. Disp.

[76] Runnerstrom EL, Llordes A, Lounis SD, Milliron DJ (2014). Nanostructured electrochromic smart windows: traditional materials and NIR-selective plasmonic nanocrystals. Chem. Commun.

[77] Liu DY, Chilton AD, Shi PJ, Craig MR, Miles SD, Dyer AL, Ballarotto VW, Reynolds JR (2011). In situ spectroscopic analysis of sub-second switching polymer electrochromes. Adv. Funct. Mater.

[78] Fernandes M, Freitas VT, Pereira S, Fortunato E, Ferreira RAS, Carlos LD, Rego R, Bermudez VDZ (2014). Green Li^+^ and Er^3+^-doped poly(ε-caprolactone)/siloxane biohybrid electrolytes for smart electrochromic windows. Sol. Energy Mater. Sol. Cells.

[79] Sun R, Bisoyi HK, Xie M, Li Q (2016). Photo and redox dual-stimuli-directed reversible disassembly and reassembly of linear supramolecular polymer formed by orthogonal host-guest molecular recognition. Dyes Pigments.

[80] Browne WR, Feringa BL (2010). Light and redox switchable molecular components for molecular electronics. Chimia.

[81] Sadler JL, Bard AJ (1968). Electrochemical reduction of aromatic azo compounds. J. Am. Chem. Soc..

[82] Neta P, Levanon H (1977). Spectrophotometric study of the radicals produced by the reduction of syn- and anti-azobenzene. J. Phys. Chem.

[83] Laviron E, Mugnier Y (1980). A study of the surface and volume electroreduction of cis- and trans-azobenzene in protic media. J. Electroanal. Chem. Interfacial Electrochem.

[84] Grampp G, Mureşanu C, Landgraf S (2005). Solvent influence on the electrochemical reduction of photochemically generated cis-azobenzene. J. Electroanal. Chem.

[85] Goulet-Hanssens A, Utecht M, Mutruc D, Titov E, Schwarz J, Grubert L, Bleger D, Saalfrank P, Hecht S (2017). Electrocatalytic Z → E isomerization of azobenzenes. J. Am. Chem. Soc..

[86] Liu ZF, Loo BH, Hashimoto K, Fujishima AJ (1991). A novel photoelectrochemical hybrid “one-way” process observed in the azobenzene system. Electroanal. Chem. Interfacial Electrochem.

[87] Zawisza I, Bilewicz R, Luboch E, Biernat JF (2000). Electrochemistry of azocrown ethers in Langmuir-Blodgett monolayers. Supramol. Chem.

[88] Jung U, Baisch B, Kaminski D, Krug K, Elsen A, Weineisen T, Raffa D, Stettner J, Bornholdt C, Herges R, Magnussen O (2008). Structure and redox behavior of azobenzene-containing monolayers on Au(111): a combined STM, X-ray reflectivity, and voltammetry study. J. Electroanal. Chem.

[89] Jung U, Müller M, Fujimoto N, Ikeda K, Uosaki K, Cornelissen U, Tuczek F, Bornholdt C, Zargarani D, Herges R, Magnussen O (2010). Gap-mode SERS studies of azobenzene-containing self-assembled monolayers on Au(111). J. Colloid Interface Sci.

[90] Kibena E, Marandi M, Maeeorg U, Venarusso LB, Maia G, Matisen L, Kasikov A, Sammelselg V, Tammeveski K (2013). Electrochemical modification of gold electrodes with azobenzene derivatives by diazonium reduction. Chem. Phys. Chem.

[91] Natsui K, Yamamoto T, Akahori M, Einaga Y (2015). Photochromism-induced amplification of critical current density in superconducting boron-doped diamond with an azobenzene molecular layer. ACS Appl. Mater. Interfaces.

[92] Chiu KY, Thai THT, Wu CG, Chang SH, Yang TF, Su YO (2017). Electrochemical studies on triarylamines featuring an azobenzene substituent and new application for small-molecule organic photovoltaics. J. Electroanal. Chem.

[93] Minagawa N, Kaneko K, Sakajo S, Yoshimoto A (1993). Effects of nitrogen bases on cyanide-resistant respiration of mitochondria isolated from *Hansenula anomala*. Biosci. Biotechnol. Biochem.

[94] Eicher T, Hauptmann S (2003). The Chemistry of Heterocycles.

[95] Yu Y, Singh SK, Liu A, Li T-K, Liu LF, LaVoie EJ (2003). Substituted dibenzo[c,h]cinnolines: topoisomerase I-targeting anticancer agents. Bioorg. Med. Chem.

[96] Barraja P, Diana P, Lauria A, Passannanti A, Almerico AM, Minnei C, Longu S, Congiu D, Musiu C, La Colla P (1999). Indolo[3,2-c]cinnolines with antiproliferative, antifungal, and antibacterial activity. Bioorg. Med. Chem.

[97] Wohlfart T (1902). Über die electrochemische Reduktion von 2,2-dinitrodiphenyl zu Phenazon und einige Derivate des Phenazons. J. Prakt. Chem.

[98] Ullmann F, Dieterle P (1904). Studien in der Diphenazonreihe. Chem. Ber.

[99] King, F.E., King, T.J.: Novel potential chemotherapeutic agents: 2. Derivatives of 2-aminobenzocinnoline. J. Chem. Soc. 824–826 (1945) 21008357

[100] Badger GM, Seidler JH, Thomson B (1951). Polynuclear heterocyclic systems. Part III. The 3:4-benzacridine-5:10-dihydro-3:4-benzacridine complex. J. Chem. Soc.

[101] Bjørsvik H-R, Gonzalez RR, Liguori L (2004). Investigation of novel process to the framework of benzo[c]cinnoline. J. Org. Chem.

[102] Slevin, Å, Koolmeister, T., Scobie, M.: A versatile synthesis of diverse 3,4-fused cinnolines via the base-catalysed condensation of 2-amino-2′-nitrobiaryls. Chem. Commun. **0** 2506–2508 (2007) 10.1039/b618318b 17563811

[103] Reddy BVS, Reddy CR, Reddy MR, Yarlagadda S, Sridhar B (2015). Substrate directed C-H activation for the synthesis of benzo[c]cinnolines through a sequential C-C and C-N bond formation. Org. Lett.

[104] Tung CH, Guan JQ (1996). Modification of photochemical reactivity of nafion. Photocyclization and photochemical cis-trans isomerization of azobenzene. J. Org. Chem.

[105] Lei Z, Vaidyalingam A, Dutta PK (1998). Photochemistry of azobenzene in microporous aluminophosphate AlPO4–5. J. Phys. Chem. B.

[106] Ide T, Ozama Y, Matusi K (2011). Photochemistry of azobenzene in sol-gel systems. J. Non-Cryst. Solids.

[107] Kaur J, Pal B (2015). Selective formation of benzo[c]cinnoline by photocatalytic reduction of 2,2′-dinitrobiphenyl using TiO_2_ and under UV light irradiation. Chem. Commun.

[108] Nakayama Y, Nakamura A, Mashima K (1997). Lanthanoid complexes of azobenzene and benzo[c]cinnoline derived from metallic lanthanoids: crystal structures of binuclear [SmI(thf)(3)(mu-eta(2):eta(2)-trans-PhNNPh)SmI(thf)(3)] and mononuclear [Yb(benzo[c]cinnoline)(3)(thf)(2)]. Chem. Lett.

[109] Volkers PI, Rauchfuss TB (2007). Extending the motif of the [FeFe]-hydrogenase active site models: protonation of Fe-2(NR)(2)(CO)(6-x)L-x species. J. Inorg. Biochem.

[110] Orain PY, Capon JF, Gloaguen F, Schollhammer P, Talarmin J (2010). Tuning of electron transfer in diiron azo-bridged complexes relevant to hydrogenases. Int. J. Hydrog. Energy.

[111] Turan AAI, Ustudag Z, Solak AO, Kilic E, Avseven A (2008). Characterization of a 2-benzo[c]cinnoline modified glassy carbon electrode by Raman spectroscopy, electrochemical impedance spectroscopy, and ellipsometry. Electroanalysis.

[112] İsbir-Turan AA, Üstündağ Z, Kılıç E, Güzel R, Uçkan Ö, Solak AO (2011). 2-Benzo[c]cinnoline and 2-benzo[c]cinnoline 6-oxide modified glassy carbon electrodes: electrocatalytic reduction of dioxygen in aqueous media. Instrum. Sci. Technol.

[113] Üstündağ Z, İsbir-Turan AA, Solak AO, Kılıç E, Avseven A (2009). Analysis of 2-benzo[c]cinnoline. nanofilm at the gold surface. Instrum. Sci. Technol.

[114] İsbir-Turan AA, Üstündağ Z, Solak AO, Kılıç E, Avseven A (2009). Electrochemical and spectroscopic characterization of a benzo[c]cinnoline electrografted platinum surface. Thin Solid Films.

[115] Öztürk F, Yazan Z, Ölmez Ö, Kılıç E, Kılıç E (2016). Electrochemical investigation of 2-[8-hydroxyquinoline-5-yl)azo]benzo[c]cinnoline on a platinum electrode in dimethysulfoxide. Turk. J. Chem.

[116] Çelik AC, Öztürk F, Erden PE, Kaçar C, Kılıç E (2015). Amperometric lactate biosensor based on carbon paste electrode modified with benzo[c]cinnoline and multiwalled carbon nanotubes. Electroanalysis.

[117] Chen JC, Wu HC, Chiang CJ, Chen T, Xing L (2014). Synthesis and properties of air-stable n-channel semiconductors based on MEH-PPV derivatives containing benzo[c]cinnoline moieties. J. Mater. Chem. C.

[118] Carstensen, O., Sielk, J., Schönborn, J.B., Granucci, G., Hartke, B.: Unusual photochemical dynamics of a bridged azobenzene derivative. J. Chem. Phys. **131**, 124305–124312 (2010) 10.1063/1.3479397 20886930

[119] Siewertsen R, Neumann H, Buchheim-Stehn B, Herges R, Näther Ch, Renth F, Temps F (2009). Highly efficient reversible Z-E photoisomerization of a bridged azobenzene with visible light through resolved S1(nπ*) absorption bands. J. Am. Chem. Soc..

[120] Siewertsen R, Schönborn JB, Hartke B, Renth F, Temps F (2011). Superior Z→E and E→Z photoswitching dynamics of dihydrodibenzodiazocine, a bridged azobenzene, by S1(nπ*) excitation at λ = 387 and 490 nm. Phys. Chem. Chem. Phys..

[121] Reuter R, Wegner HA (2011). Oligoazobenzenophanes - synthesis, photochemistry and properties. Chem. Commun.

[122] Slavov Ch, Yang Ch, Schweighauser L, Wegner HA, Dreuw A, Wachtveitl J (2017). Ultrafast excited-state deactivation dynamics of cyclotrisazobenzene—a novel type of UV-B absorber. Chem.Phys. Chem.

[123] Norikane Y, Kitamoto K, Tamaoki N (2003). Novel crystal structure, cis/trans isomerization, and host property of meta-substituted macrocyclic azobenzenes with the shortest linkers. J. Org. Chem.

[124] Norikane, Y., Tamaoki, N.: Photochemical and thermal cis/trans isomerization of cyclic and noncyclic azobenzene dimers: effect of a cyclic structure on isomerization. Eur. J. Org. Chem. **2006**, 1296–1302 (2006)

[125] Norikane Y, Hirai Y, Yoshida M (2011). Photoinduced isothermal phase transitions of liquid crystalline macrocyclic azobenzenes. Chem. Commun.

[126] Müri, M., Schuermann, K.C., De Cola, L., Mayor, M.: Shape-switchable azo-macrocycles. Eur. J. Org. Chem. **2009**, 2562–2575 (2009)

[127] Reuter, R., Hostettler, N., Neuburger, M., Wegner, H.A.: Synthesis and property studies of cyclotrisazobenzenes. Eur. J. Org. Chem. **2009**, 5647–5652 (2009)

[128] Reuter R, Wegner HA (2013). Switchable 3D networks by light controlled π-stacking of azobenzene macrocycles. Chem. Commun.

[129] Schweighauser L, Häussinger D, Neuburger M, Wegner HA (2014). Symmetry as a new element to control molecular switches. Org. Biomol. Chem.

[130] Shen JT, Guan L, Zhu XY, Zeng QD, Wang Ch (2009). Submolecular observation of photosensitive macrocycles and their isomerization effects on host-guest network. J. Am. Chem. Soc..

[131] Reuter R, Wegner HA (2011). A chiral cyclotrisazobiphenyl: synthesis and photochemical properties. Org. Lett.

[132] Shiga, M., Takagi, M., Ueno, K.: Azo-crown ethers: the dyes with azo group directly involved in the crown ether skeleton. Chem. Lett. 1021–1022 (1980)

[133] Shiga M, Nakamura H, Takagi M, Ueno K (1984). Synthesis of azobenzo-crown ethers and their complexation behavior with metal-ions. Bull. Chem. Soc. Jpn.

[134] Biernat JF, Luboch E, Cygan A, Simonov YA, Dvorkin AA, Muszalska E, Bilewicz R (1992). Synthesis, X-ray structure and electrochemical properties of a new crown-ether with a cis azo unit in the macrocycle. Tetrahedron.

[135] Biernat JF, Cygan A, Luboch E, Simonov YA, Dvorkin AA (1993). 13-membered crown-ether with an azoxy subunit in the macrocycle—synthesis and X-ray structure. J. Incl. Phenom.

[136] Luboch E, Biernat JF, Muszalska E, Bilewicz R (1995). 13-Membered crown-ethers with azo or azoxy unit in the macrocycle - synthesis, membrane electrodes, voltammetry and langmuir monolayers. Supramol. Chem.

[137] Luboch E, Biernat JF, Simonov YA, Dvorkin AA (1998). Synthesis and electrode properties of 16-membered azo- and azoxycrown ethers. Structure of tribenzo-16-azocrown-6. Tetrahedron.

[138] Pijanowska DG, Luboch E, Biernat JF, Dawgul M, Torbicz W (1999). Na^+^-selective ChemFETs based on a novel ionophore: bis(phenylbenzo)-13-azocrown-5. Sens. Actuator B.

[139] Zawisza I, Bilewicz R, Luboch E, Biernat JF (1999). Properties of Z and E isomers of azocrown ethers in monolayer assemblies at the air-water interface. Thin Solid Films.

[140] Luboch E, Wagner-Wysiecka E, Kravtsov VC, Kessler V (2003). Characterization of small azocrown ether stereoisomers. Pol. J. Chem.

[141] Shinkai S, Manabe O (1984). Photocontrol of ion extraction and ion-transport by photofunctional crown ethers. Topics Curr. Chem.

[142] Shinkai S, Takeuchi M, Ikeda A, De Rossi DE, Osada Y (2000). Molecular machines useful for the design of chemosensor. In Polymer Sensors and Actuators.

[143] Shinkai S, Minami T, Kusano Y, Manabe O (1983). Photoresponsive crown ethers. 8. Azobenzenophane-type switched-on crown ethers which exhibit an all-or-nothing change in ion-binding ability. J. Am. Chem. Soc..

[144] Miao Y, Wang X, Ouyang D (2012). Theoretical study of crown ethers with incorporated azobenzene moiety. J. Mol. Model..

[145] Li WW, Yu XL, Wang XY (2017). Theoretical design of a new allosteric switch and fluorescence chemosensor double functional devices of aza-crown ether. J. Phys. Chem. C.

[146] Oka Y, Tamaoki N (2010). Structure of silver(I) complex prepared from azobenzenonaphthalenophane, photochemical coordination change of silver(I) and silver(I)-induced acceleration of Z-E thermal isomerization of azobenzene unit. Inorg. Chem.

[147] Lyapunov A, Kirichenko T, Kulygina C, Zubatyuk R, Fonari M, Kyrychenko A, Doroshenko A (2015). New fluorenonocrownophanes containing azobenzene: synthesis, properties and interactions with paraquat. J. Incl. Phenom. Macrocycl. Chem.

[148] Luboch E, Biernat JF, Kravtsov VC, Simonov YA (1998). 13-Membered azocrown ether. Structure of the lithium bromide complex and membrane properties. J. Incl. Phenom.

[149] Simonov YA, Luboch E, Biernat JF, Bolotina NV, Zavodnik VE (1997). Inclusion compounds of NaI with 13-membered azo- and azoxycrown ethers. J. Incl. Phenom.

[150] Luboch E, Biernat JF, Simonov YA, Kravtsov VC, Bel’skii VK (1999). Structures of NaI complexes of 16-membered azo- and azoxycrown ethers. Correlation of crystal structure and carrier-doped membrane electrode selectivity. Supramol. Chem.

[151] Skwierawska A, Luboch E, Biernat JF, Kravtsov VC, Simonov YA, Dvorkin AA, Bel’skii VK (1998). Stereochemistry of 16-membered azo- and azoxycrown ethers. Structures of their sandwich potassium iodide complexes. J. Incl. Phenom.

[152] Biernat JF, Luboch E, Coleman AW (1998). How can X-ray structures be helpful for design of ionophores for ion-selective membrane electrodes?. Molecular Recognition and Inclusion.

[153] Fonari MS, Luboch E, Collas A, Bukrej A, Blockhuys F, Biernat JF (2008). Molecular structures of two E-azobenzocrown ethers. J. Mol. Struct.

[154] Shimizu FM, Volpati D, Giacometti JA, Sworakowski J, Janus K, Luboch E (2007). Kinetic of fotoinduced birefringence in the guest-host system of poly(methyl methacrylate) doped with azobenzene-containing crown ethers. J. Appl. Polym. Sci.

[155] Shimizu FM, Giacometti JA, Luboch E, Biernat JF, Ferreira M (2009). Preparation and characterization of Langmuir-Blodgett films of 16-membered azobenzocrown ether with naphthalene residue. Synth. Met..

[156] Shimizu FM, Ferreira M, Skwierawska AM, Biernat JF, Giacometti JA (2012). Spectroscopy and electrochemical characterization of Langmuir–Blodgett and physical vapor thin films of 29-membered diazocrown ether 1 with two n-octyl substituents. Synth. Met.

[157] Kertmen A, Szczygelska-Tao J, Chojnacki J (2013). Azo and azoxythiacrown ethers: synthesis and properties. Tetrahedron.

[158] Luboch E, Wagner-Wysiecka E, Biernat JF (2002). Chromogenic azocrown ethers with peripheral alkyl, alkoxy, hydroxy or dimethylamino group. J. Supramol. Chem.

[159] Luboch E, Wagner-Wysiecka E, Poleska-Muchlado Z, Kravtsov VC (2005). Synthesis and properties of azobenzocrown ethers with π-electron donor, or π-electron donor and π-electron acceptor group(s) on benzene ring(s). Tetrahedron.

[160] Luboch E, Wagner-Wysiecka E, Rzymowski T (2009). 4-Hexylresorcinol-derived hydroxyazobenzocrown ethers as chromoionophores. Tetrahedron.

[161] Luboch E (2008). The Wallach rearrangement as a method for the synthesis of functionalized azobenzocrown ethers. Pol. J. Chem.

[162] Szarmach M, Wagner-Wysiecka E, Fonari MS, Luboch E (2012). Bis(azobenzocrown ether)s—synthesis and ionophoric properties. Tetrahedron.

[163] Luboch E, Jeszke M, Szarmach M, Łukasik N (2016). New bis(azobenzocrown)s with dodecylmethylmalonyl linkers as ionophores for sodium selective potentiometric sensors. J. Incl. Phenom. Macrocycl. Chem.

[164] Luboch E, Szarmach M, Buczkowska A, Wagner-Wysiecka E, Kania M, Danikiewicz W (2015). Synthesis of thiol derivatives of azobenzocrown ethers. The preliminary studies on recognition of alkali metal ions by gold nanoparticles functionalized with azobenzocrown and lipoic acid. J. Incl. Phenom. Macrocycl. Chem.

[165] Szarmach M, Wagner-Wysiecka E, Luboch E (2013). Rearrangement of azoxybenzocrowns into chromophoric hydroxyazobenzocrowns and the use of hydroxyazobenzocrowns for the synthesis of ionophoric biscrown compounds. Tetrahedron.

[166] Tahara R, Morozumi T, Nakamura H, Shimomura M (1997). Photoisomerisation of azobenzocrown ethers. Effect of complexation of alkaline earth metal ions. J. Phys. Chem. B.

[167] Aoki S, Shiga M, Tazaki M, Nakamura H, Takagi M, Ueno K (1981). Ion-dipole association chromatography on ion-exchanger in non-aqueous media. Separation and characterization of crown ethers and related compounds. Chem. Lett.

[168] Cadogan A, Gao Z, Lewenstam A, Ivaska A (1992). All-solid-state sodium-selective electrode based on a calixarene ionophore in a poly(vinyl chloride) membrane with a polypyrrole solid contact. Anal. Chem.

[169] Ammann D, Anker P, Metzger E, Oesch U, Simon W, Kessler M, Harrison DK, Höper J (1985). Ion measurements in physiology and medicine. Proceedings of the International Symposium on the Theory and Application of Ion Selective Electrodes in Physiology and Medicine.

[170] Pang J, Ye Y, Tian Z, Pang X, Wu C (2015). Theoretical insight into azobis-(benzo-18-crown-6) ether combined with the alkaline earth metal cations. Comp. Theor. Chem.

[171] Yang R-Y, Bao C-Y, Lin Q-N, Zhu L-Y (2015). A light-regulated synthetic ion channel constructed by an azobenzene modified hydraphile. Chin. Chem. Lett.

[172] Gromov SP, Vedernikov AI, Ushakov EN, Alfimov MV (2008). Unusual supramolecular donor-acceptor complexes of bis(crown)stilbenes and bis(crown)azobenzene with viologen analogs. Russ. Chem. Bull. Int. Ed.

[173] Antonov LM, Kurteva VB, Simeonov SP, Deneva VV, Crochet A, Fromm KM (2010). Tautocrowns: a concept for a sensing molecule with an active side-arm. Tetrahedron.

[174] Ioannidis M, Gentleman AS, Ho L, Lincoln SF, Sumby ChS (2010). Complexation and structural studies of a sulfonamide aza-15-crown-5 derivative. Inorg. Chem. Commun.

[175] Schultz RA, Dishong DM, Gokel GW (1982). Lariat ethers. 4. Chain length and ring size effects in macrocyclic polyethers having neutral donor groups on flexible arms. J. Am. Chem. Soc..

[176] Schultz RA, White BD, Dishong DM, Arnold KA, Gokel GW (1985). 12-, 15-, and 18-Membered-ring nitrogen-pivot lariat ethers: syntheses, properties, and sodium and ammonium cation binding properties. J. Am. Chem. Soc..

[177] Steed JW (2001). First- and second-sphere coordination chemistry of alkali metal crown ether complexes. Coord. Chem. Rev.

[178] Gokel G (1991). Crown Ethers and Cryptands.

[179] Lee H, Lee SS (2009). Thiaoxaaza-macrocyclic chromoionophores as mercury(II) sensors: synthesis and color modulation. Org. Lett.

[180] Seo J, Park S, Lee SS, Fainerman-Melnikova M, Lindoy LF (2009). Copper(II) interaction with mono-, bis- and tris-ring N3O2 macrocycles: synthetic, X-ray, competitive membrane transport, and hypochromic shift studies. Inorg. Chem.

[181] Lee SJ, Lee JE, Seo J, Jeong IY, Lee SS, Jung JH (2007). Optical sensor based on nanomaterial for the selective detection of toxic metal ions. Adv. Funct. Mater.

[182] Jeon CH, Lee J, Ahn SJ, Ha TH (2013). Solvent effect and amine interference on colorimetric changes of azobenzene-conjugated dithiaazadioxo crown ether mercury sensor. Tetrahedron Lett.

[183] Jeon CH, Ha TH (2015). Surfactant effect upon colorimetric mercury chemosensor based on azobenzene-conjugated dithiaazadioxo crown ether. Bull. Korean. Chem. Soc.

[184] Wagner-Wysiecka E, Rzymowski T, Szarmach M, Fonari MS, Luboch E (2013). Functionalized azobenzocrown ethers as sensor materials—the synthesis and ion binding properties. Sens. Actuator B-Chem.

[185] Bershtein IY, Ginzburg OF (1972). Tautomerism of aromatic azo-compounds. Russ. Chem. Rev.

[186] Luboch E, Kravtsov VC (2004). Molecular structures and supramolecular architectures of two chromogenic 13-membered azobenzocrown ethers with a peripheral hydroxyl group in the benzene ring. J. Mol. Struct.

[187] Shimomura M, Kunitake T (1987). Fluorescence and photoisomerization of azobenzene-containing bilayer membranes. J. Am. Chem. Soc..

[188] Tsuda K, Dol GC, Gensch T, Hofkens J, Latterini L, Weener JW, Meijer EW, De Schryver FC (2000). Fluorescence from azobenzene functionalized poly(propyleneimine) dendrimers in self-assembled supramolecular structures. J. Am. Chem. Soc..

[189] Han M, Hara M (2005). Intense fluorescence from light-driven self-assembled aggregates of nonionic azobenzene derivative. J. Am. Chem. Soc..

[190] Tung C-H, Guan J-Q (1996). Modification of photochemical reactivity by nafion. Photocyclization and photochemical cis–trans isomerization of azobenzene. J. Org. Chem.

[191] Wagner-Wysiecka E, Szarmach M, Chojnacki J, Łukasik N, Luboch E (2017). Cation sensing by diphenyl-azobenzocrowns. J. Photochem. Photobiol. A.

[192] Wagner-Wysiecka E, Luboch E, Kowalczyk M, Biernat JF (2003). Chromogenic macrocyclic derivatives of azoles-synthesis and properties. Tetrahedron.

[193] Luboch E, Wagner-Wysiecka E, Fainerman-Melnikova M, Lindoy LF, Biernat JF (2006). Pyrrole azocrown ethers. Synthesis, complexation, selective lead transport and ion-selective membrane electrode studies. Supramol. Chem.

[194] Wagner-Wysiecka E, Rzymowski T, Fonari MS, Kulmaczewski R, Luboch E (2011). Pyrrole azocrown ethers—synthesis, crystal structures, and fluorescence properties. Tetrahedron.

[195] Wagner-Wysiecka E, Rzymowski T, Luboch E (2008). Metal cation complexation by pyrrole-containing chromogenic macrocycle. Pol. J. Chem.

[196] Wagner-Wysiecka E, Luboch E, Fonari MS (2008). The synthesis, X-ray structure and metal cation complexation properties of colored crown with two heterocyclic residues as a part of macrocycle. Pol. J. Chem.

[197] Wagner-Wysiecka E, Jamrógiewicz M, Fonari MS, Biernat JF (2007). Azomacrocyclic derivatives of imidazole: synthesis, structure, and metal ion complexation properties. Tetrahedron.

[198] Sadowska K, Jamrógiewicz M, Biernat J (2008). Stimulated by cyclodextrins high yield synthesis of azocrown analogues comprising pyrrole or imidazole residue. Supramol. Chem.

[199] Kledzik K, Jamrógiewicz M, Gwiazda M, Wagner-Wysiecka E, Jezierska J, Biernat JF, Kłonkowski AM (2007). Optical recognition elements. Macrocyclic imidazole chromoionophores entrapped in silica xerogel. Mater. Sci. Pol.

[200] Jabłonowska E, Pałys B, Wagner-Wysiecka E, Jamrógiewicz M, Biernat JF, Bilewicz R (2007). pH-tunable equilibria in azocrown ethers with histidine moieties. Bioelectrochemistry.

[201] Yamamoto T, Nakamura D, Liu G, Nishinaka K, Tsuda A (2016). Synthesis and photoisomerization of an azobenzene-containing tetrapyrrolic macrocycle. J. Photochem. Photobiol. A Chem.

[202] Ryan STJ, del Barrio J, Suardíaz R, Ryan DF, Rosta E, Scherman OA (2016). A dynamic and responsive host in action: light-controlled molecular encapsulation. Angew. Chem. Int. Ed.

[203] Osorio-Planes L, Espelt M, Pericàs MA, Ballester P (2014). Reversible photocontrolled disintegration of a dimeric tetraurea-calix[4]pyrrole capsule with all-trans appended azobenzene units. Chem. Sci.

[204] Gokulnath S, Prabhuraja V, Sankar J, Chandrashekar TK (2007). Smaragdyrin-azobenzene conjugates: syntheses, structure, and spectra and electrochemical properties. Eur. J. Org. Chem.

[205] Schuster DI, Li K, Guldi DM, Palkar A, Echegoyen L, Stanisky Ch, Cross RJ, Niemi M, Tkachenko NV, Lemmetyinen H (2007). Azobenzene-linked porphyrin-fullerene dyads. J. Am. Chem. Soc..

[206] Gutsche, C.D., Kung, T.C., Hsu, M.-L.: Abstracts of the 11th Midwest Regional Meeting of the American Chemical Society, Carbondale IL 517 (1975)

[207] Perrin R, Lamartine R, Perrin M (1993). The potential industrial applications of calixarenes. Pure Appl. Chem.

[208] Seiffarth K, Schulz M, Goermar G, Bachmann J (1989). Calix[n]arenes-new light stabilizers for polyolefins. Polym. Degrad. Stabil.

[209] Atanassova M, Kurteva V (2016). Synergism as a phenomenon in solvent extraction of 4f-elements with calixarenes. RSC Adv.

[210] Mokhtari B, Pourabdollah K (2011). Binding abilities and extractive applications of nano-baskets of calixarenes. Asian J. Chem.

[211] Mokhtari B, Pourabdollah K, Dallali N (2011). A review of calixarene applications in nuclear industries. J. Radioanal. Nucl. Chem.

[212] Ludwig R (2000). Calixarenes in analytical and separation chemistry. Fresenius J. Anal. Chem.

[213] Klejch T, Slavicek J, Hudecek O, Eigner V, Gutierrez NA, Curinova P, Lhotak P (2016). Calix[4]arenes containing a ureido functionality on the lower rim as highly efficient receptors for anion recognition. New J. Chem.

[214] Gomez-Machuca H, Quiroga-Campano C, Jullian C, De la Fuente J, Pessoa-Mahana H, Escobar CA, Dobado JA, Saitz C (2014). Study by fluorescence of calix[4]arenes bearing heterocycles with anions: highly selective detection of iodide. J. Incl. Phenom. Macrocycl. Chem.

[215] Tlustý M, Slavík P, Dvořáková H, Eigner V, Lhoták P (2017). Synthesis and study of calix[4]arenes bearing azo moieties at the meta position. Tetrahedron.

[216] Chawla HM, Singh SP, Sahu SN, Upreti S (2006). Shaping the cavity of calixarene architecture for molecular recognition: synthesis and conformational properties of new azocalix[4]arenes. Tetrahedron.

[217] Galán H, Hennrich G, de Mendoza J, Prados P (2010). Synthesis and photoisomerization of azocalixarenes with dendritic structures. Eur. J. Org. Chem.

[218] Bonvallet PA, Mullen MR, Evans PJ, Stoltz KL, Story EN (2011). Improved functionality and control in the isomerization of a calix[4]arene-capped azobenzene. Tetrahedron Lett.

[219] Arduini, A., Pochini, A., Secchi, A.: Rigid calix[4]arene as a building block for the synthesis of new quaternary ammonium cation receptors. Eur. J. Org. Chem.** 2000**, 2325–2334 (2000)

[220] Park SJ, Choe J-I (2008). DFT study for azobenzene crown ether p-tert-butylcalix[4]arene complexed with alkali metal ion. Bull. Korean Chem. Soc.

[221] Lee HG, Seo J, Choi KS (2013). Synthesis and color modulation of an unsymmetrical calix[4]-bis-crown incorporating p-nitroazobenzene as a chromoionophoric mixed receptor. Bull. Korean Chem. Soc.

[222] Jin T (2007). Calixarene–based photoresponsive ion carrier for the control of Na^+^ flux across a lipid bilayer membrane by visible light. Mater. Lett..

[223] Lakomehsari KR, Ganjali ST, Zadmard R, Roshan M (2017). A novel azo-calixaren derivative based on 2,6-diamino pyridine: synthesis, characterization and antibacterial evaluation. Lett. Org. Chem.

[224] Villiers A (1891). Fermentation of starch by the butyric ferment. Compt. Rend.

[225] Rekharsky MV, Inoue Y (1998). Complexation thermodynamics of cyclodextrins. Chem. Rev.

[226] Ma X, Tian H (2014). Stimuli-responsive supramolecular polymers in aqueous solution. Acc. Chem. Res.

[227] Harada A, Takashima Y, Nakahata M (2014). Supramolecular polymeric materials via cyclodextrin–guest interactions. Acc. Chem. Res.

[228] Nakahata M, Takashima Y, Harada A (2017). Supramolecular polymeric materials containing cyclodextrins. Chem. Pharm. Bull.

[229] Tan S, Ladewig K, Fu Q, Blencowe A, Qiao GG (2014). Cyclodextrin-based supramolecular assemblies and hydrogels: recent advances and future perspectives. Macromol. Rapid Commun.

[230] Chen Y, Liu Y (2010). Cyclodextrin-based bioactive supramolecular assemblies. Chem. Soc. Rev.

[231] Zhou J, Ritter H (2010). Cyclodextrin functionalized polymers as drug delivery systems. Polym. Chem.

[232] Yamamura H (2017). Chemical modification of cyclodextrin and amylose by click reaction and its application to the synthesis of poly-alkylamine-modified antibacterial sugars. Chem. Pharm. Bull.

[233] Arima H, Motoyama K, Higashi T (2017). Potential use of cyclodextrins as drug carriers and active pharmaceutical ingredients. Chem. Pharm. Bull.

[234] Wong CE, Dolzhenko AV, Lee SM, Young DJ (2017). Cyclodextrins: a weapon in the fight against antimicrobial resistance. J. Mol. Eng. Mater..

[235] Schmidt BVKJ, Barner-Kowollik C (2017). Dynamic macromolecular material design—the versatility of cyclodextrin-based host-guest chemistry. Angew. Chem. Int. Ed.

[236] Hapiot F, Monflier E (2017). Unconventional approaches involving cyclodextrin-based, self-assembly-driven processes for the conversion of organic substrates in aqueous biphasic catalysis. Catalysts.

[237] Taka AL, Pillay K, Mbianda XY (2017). Nanosponge cyclodextrin polyurethanes and their modification with nanomaterials for the removal of pollutants from waste water: a review. Carbohydr. Polym.

[238] Fanali S (2017). Nano-liquid chromatography applied to enantiomers separation. J. Chromatogr. A.

[239] Kalikova K, Slechtova T, Tesarova E (2017). Cyclic oligosaccharide-based chiral stationary phases applicable to drug purity control: a review. Curr. Med. Chem.

[240] Rezanka M (2016). Monosubstituted cyclodextrins as precursors for further use. Eur. J. Org. Chem.

[241] Lay S, Ni X, Yu H, Shen S (2016). State-of-the-art applications of cyclodextrins as functional monomers in molecular imprinting techniques: a review. J. Sep. Sci.

[242] Liu Y, Yang Z-X, Chen Y (2008). Syntheses and self-assembly behaviors of the azobenzenyl modified β-cyclodextrins isomers. J. Org. Chem.

[243] Ma X, Wang Q, Tian H (2007). Disparate orientation of [1]rotaxanes. Tetrahedron Lett.

[244] Casas-Solvas JM, Martos-Maldonado MC, Vargas-Berenguel A (2008). Synthesis of β-cyclodextrin derivatives functionalized with azobenzene. Tetrahedron.

[245] Casas-Solvas JM, Vargas-Berenguel A (2008). Synthesis of a β-cyclodextrin derivative bearing an azobenzene group on the secondary face. Tetrahedron Lett.

[246] Sun H-L, Chen Y, Zhao J, Liu Y (2015). Photocontrolled reversible conversion of nanotube and nanoparticle mediated by β-cyclodextrin dimers. Angew. Chem. Int. Ed.

[247] Hamon F, Blaszkiewicz C, Buchotte M, Banaszak-Léonard E, Bricout H, Tilloy S, Monflier E, Cézard C, Bouteiller L, Len C, Djedaini-Pilard F (2014). Synthesis and characterization of a new photoinduced switchable β-cyclodextrin dimer. Beilstein J. Org. Chem.

[248] Jog PV, Gin MS (2008). A light-gated synthetic ion channel. Org. Lett.

[249] Jung JH, Lee SJ, Kim JS, Lee WS, Sakata Y, Kanedar T (2006). α-CD/crown-appended diazophenol for selective sensing of amines. Org. Lett.

[250] Fujita K, Fujiwara S, Yamada T, Tsuchido Y, Hashimoto T, Hayashita T (2017). Design and function of supramolecular recognition systems based on guest-targeting probe-modified cyclodextrin receptors for ATP. J. Org. Chem.

[251] Chen X, Liu Z, Parker SG, Zhang X, Gooding JJ, Ru Y, Liu Y, Zhou Y (2016). Light-induced hydrogel based on tumor-targeting mesoporous silica nanoparticles as a theranostic platform for sustained cancer treatment. ACS Appl. Mater. Interfaces.

[252] Anand R, Manoli F, Vargas-Berenguel A, Monti S (2012). Photocontrolled binding of artemisinin to a bis(β-cyclodextrin) bearing azobenzene on the primary face. J. Drug Deliv. Sci. Technol.

[253] Zhao Y, Ikeda T (2009). Smart Light Responsive Materials Azobenzene-Containing Polymers and Liquid Crystals.

[254] Ma H, Wang F, Li W, Ma Y, Yao X, Lu D, Yang Y, Zhang Z, Lei Z (2014). Supramolecular assemblies of azobenzene-β-cyclodextrin dimers and azobenzene modified polycaprolactones. J. Phys. Org. Chem.

[255] Tomatsu I, Hashidzume A, Harada A (2005). Photoresponsive hydrogel system using molecular recognition of α-cyclodextrin. Macromolecules.

[256] Tomatsu I, Hashidzume A, Harada A (2006). Contrast viscosity changes upon photoirradiation for mixtures of poly(acrylic acid)-based α-cyclodextrin and azobenzene polymers. J. Am. Chem. Soc..

[257] Inoue Y, Kuad P, Okumura Y, Takashima Y, Yamaguchi H, Harada A (2007). Thermal and photochemical switching of conformation of poly(ethylene glycol)-substituted cyclodextrin with an azobenzene group at the chain end. J. Am. Chem. Soc..

[258] Tokuhisa H, Kimura K, Yokoyama M, Shinkai S (1995). Ion-conducting behaviour and photoinduced ionic-conductivity switching of composite films containing crowned cholesteric liquid crystals. J. Chem. Soc. Faraday Trans.

[259] Shibaev V, Medvedev A, Bobrovsky A (2008). Photochromic LC copolymers containing azobenzene and crown-ether groups. J. Polym. Sci. A.

[260] Ryabchun A, Bobrovsky A, Medvedev A, Shibaev V (2011). Crown ether and azobenzene-containing liquid crystalline polymers: an influence of macromolecular architecture on optical properties and photo-orientation processes. J. Polym. Sci. A.

[261] Sun Y, Wang Z, Li Y, Zhang Z, Zhang W, Pan X, Zhou N, Zhu X (2015). Photoresponsive amphiphilic macrocycles containing main-chain azobenzene polymers. Macromol. Rapid Commun.

[262] Kolb HC, Finn MG, Sharpless KB (2001). Click chemistry: diverse chemical function from a few good reactions. Angew. Chem. Int. Ed.

[263] Wiktorowicz S, Duchêne R, Tenhu H, Aseyev V (2014). Multi-stimuli responsive poly(azodibenzeno-18-crown-6-ether)s. Polym. Chem.

[264] Iftime M, Ardeleanu R, Fifere N, Airinei A, Cozan V, Bruma M (2014). New copoly(ether sulfone)s containing azobenzene crown-ether and fluorene moieties. Dyes Pigments.

[265] Dong S, Gao L, Li J, Xu D, Zhou Q (2013). Photo-responsive linear and cross-linked supramolecular polymers based on host-guest interactions. Polym. Chem.

[266] Jiang X, Lu J, Zhou F, Zhang Z, Pan X, Zhang W, Wang Y, Zhou N, Zhu X (2016). Moleculary-defined macrocycles containing azobenzene main-chain oligomers: modular stepwise synthesis, chain-length and topology-dependent properties. Polym. Chem.

[267] Lu J, Zhou F, Li L, Zhang Z, Meng F, Zhou N, Zhu X (2016). Novel cyclic azobenzene-containing vesicles: photo/reductant responsiveness and potential applications in colon disease treatment. RSC Adv.

[268] Qiu X-P, Korchagina EV, Rolland J, Winnik FM (2014). Synthesis of a poly(N-isopropylacrylamide) charm bracelet decorated with a photomobile α-cyclodextrin charm. Polym. Chem.

[269] Ogoshi T, Yoshikoshi K, Aoki T, Yamagishi T (2013). Photoreversible switching between assembly and disassembly of a supramolecular polymer involving an azobenzene-bridged pillar[5]arene dimer. Chem. Commun.

[270] Berryman OB, Sather AC, Rebek J (2011). A light controlled cavitand wall regulates guest binding. Chem. Commun.

[271] Malek-Ahmadi S, Abdolmaleki A (2011). Synthesis and characterization of new azo containing Schiff base macrocycle. Chin. J. Chem.

[272] Tanaka K, Fukuoka S, Miyanishi H, Takahashi H (2010). Novel chiral Schiff base macrocycles containing azobenzene chromophore: gelation and guest inclusion. Tetrahedron Lett.

[273] Benniston AC, Harriman AA, Yang S, Harrington RW (2011). Highly-strained cyclophanes bearing both photo- and electro-active constituents. Tetrahedron Lett.

[274] Tang H-S, Zhu N, Yam VW-W (2007). Tetranuclear macrocyclic gold(I) alkynyl phosphine complex containing azobenzene functionalities: a dual-input molecular logic with photoswitching behavior controllable via silver(I) coordination/decoordination. Organometallics.

[275] Kinbara K, Muraoka T, Aida T (2008). Chiral ferrocenes as novel rotary modules for molecular machines. Org. Biomol. Chem.

[276] Azov VA, Cordes J, Schlüter D, Dülcks T, Böckmann M, Doltsinis NL (2014). Light-controlled macrocyclization of tetrathiafulvalene with azobenzene: designing an optoelectronic molecular switch. J. Org. Chem.

[277] Chandra S, Kundu T, Kandambeth S, BabaRao R, Marathe Y, Kunjir SM, Banerjee R (2014). Phosphoric acid loaded azo (-N = N-) based covalent organic framework for proton conduction. J. Am. Chem. Soc..

[278] Schutt M, Krupka SS, Milbradt AG, Deindl S, Sinner EK, Oesterhelt D, Renner C, Moroder L (2003). Photocontrol of cell adhesion processes: model studies with cyclic azobenzene-RDG peptides. Chem. Biol.

[279] Hoppmann C, Seedorff S, Richter A, Fabian H, Schmieder P, Rück-Braun K, Beyermann M (2009). Light-directed protein binding of a biologically relevant beta-sheet. Angew. Chem. Int. Ed. Engl.

[280] Löweneck M, Milbrandt AG, Root C, Satzger H, Zinth W, Moroder L, Renner C (2006). A conformational two-state peptide model system containing an ultrafast but soft light switch. Biophys. J.

[281] Jafari MR, Lakusta J, Lundgren RJ, Derda R (2016). Allene functionalized azobenzene linker enables rapid and light-responsive peptide macrocylization. Bioconjugate Chem.

[282] Beharry AA, Sadovski O, Woolley GA (2008). Photo-control of peptide conformation on a timescale of seconds with conformationally constrained, blue-absorbing, photo-switchalble linker. Org. Biomol. Chem.

[283] Despras G, Hain J, Jaeschke SO (2017). Photocontrol over molecular shape: synthesis and photochemical evaluation of glycoazobenzene macrocyles. Chem. Eur. J.

