# Correction to: Azo group(s) in selected macrocyclic compounds

**DOI:** 10.1007/s10847-018-0788-y

**Published:** 2018-02-24

**Authors:** Ewa Wagner-Wysiecka, Natalia Łukasik, Jan F. Biernat, Elżbieta Luboch

**Affiliations:** Department of Chemistry and Technology of Functional Materials, Faculty of Chemistry, Gdańsk University of Technology, Narutowicza Street 11/12, 80-233 Gdańsk, Poland

## Correction to: Journal of Inclusion Phenomena and Macrocyclic Chemistry 10.1007/s10847-017-0779-4

In the original publication of the article, a part of Scheme 12 was missed. The correct version of Scheme 12 was provided in this correction article. The original article has also been corrected.

**Scheme 12:**
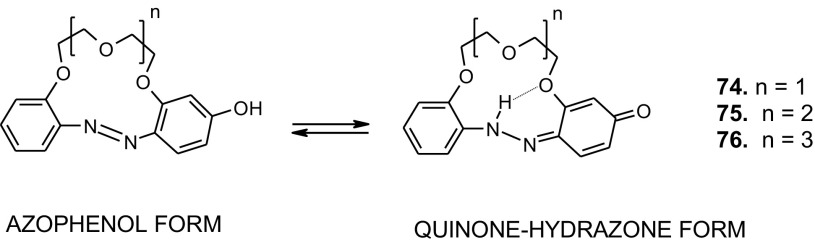
Tautomeric equilibrium for hydroxyazobenzocrown ethers **74–76** showing hydrogen bond inside the cavity of quinone-hydrazone form [158]

